# Control of alluvial aquifer architecture on reductive and oxidative dechlorination of chloroethenes

**DOI:** 10.1007/s11356-026-37894-7

**Published:** 2026-06-20

**Authors:** Diana Puigserver, Jofre Herrero, Amparo Cortés, Alberto Millán, Beth L. Parker, José María Carmona

**Affiliations:** 1 Department of Mineralogy, Petrology and Applied Geology, Faculty of Earth Sciences, University of Barcelona (UB), C/Martí I Franquès, S/N, 08028 Barcelona, Spain; 2 Department of Biology, Healthcare and the Environment, Faculty of Pharmacy and Food Sciences, University of Barcelona (UB), C/Joan XXIII, 27-31, 08028 Barcelona, Spain; 3 School of Engineering, University of Guelph, 50, Stone Road East, Guelph, N1G 2W1 ON Canada

**Keywords:** Aquifer-hyporheic zone interaction, Subsurface geological structure, Hydrogeological heterogeneity, Subsurface redox conditions, Dechlorinating microbial communities, Chloroethene dechlorination

## Abstract

Groundwater quality and ecosystem function are significantly influenced by aquifer-hyporheic zone systems, particularly in cases where such systems interact with influent rivers. This study investigates the impact of the geological structure on natural attenuation of chloroethenes in such connected systems. Our research focuses on understanding the complex interactions between geological features and microbial dynamics, shedding light on perchloroethylene transformation from DNAPL sources. Field investigations were conducted in an alluvial aquifer with fluvial paleochannels dominated by gravels and sands separated by silts and clays in floodplains. A multidisciplinary approach was employed, combining sediment geochemistry, chloroethene isotopic fractionation (δ^13^C), and microbial community characterization. The results revealed distinct microbial assemblages associated with different geological structures, highlighting hydrogeological heterogeneity’s role in shaping microbial diversity and activity. Specific microbial genera involved in chloroethene dechlorination across paleochannels, interchannel areas, and the hyporheic zone on the bank of an influent river were identified, illustrating differential microbial contributions to contaminant degradation. Furthermore, the study elucidated medium factors such as hydraulic conductivity and organic carbon availability that modulate microbial community structure and function. These findings enhance understanding of microbial-mediated contaminant attenuation in heterogeneous subsurface environments, informing groundwater remediation strategies and supporting sustainable groundwater management and environmental protection.

**Supplementary Information:**

The online version contains supplementary material available at 10.1007/s11356-026-37894-7.

## Introduction

The contamination of groundwater and surface water bodies represents a growing issue (Zanotti et al. 2019). This problem is exacerbated when these water bodies are interconnected, leading to contamination affecting both systems (Epting et al. 2018) and thereby posing a significant risk to aquatic ecosystems and human health (Conant et al. 2019). Among the most common forms of contamination are diffuse pollution, especially by nitrogenous species (Pinardi et al. 2022), and to a lesser extent, although it is no less important, point-source contamination (Mojarrad et al. 2019). Within this latter category, episodes of contamination by chloroethenes are notable, as described by Weatherill et al. (2019), Rivett et al. (2014), Rowe et al. (2007), and Burston et al. (1993).

The presence of chloroethenes is not only limited to detection in groundwater; they are also increasingly recorded in surface water bodies (as indicated, for example, by Ottosen et al. 2020; Wittlingerová et al. 2016; Ellis and Rivett 2007; McGuire et al. 2004; Christof et al. 2002). This widespread contamination is attributed to groundwater discharges resulting from the river baseflow, which is considered one of the main causes contributing significantly to the global degradation of the ecological quality of surface waters, a phenomenon known as urban stream syndrome (Roy et al. 2016; Roy and Bickerton 2012; Meyer et al. 2005).

Chloroethenes, consisting of perchloroethene (PCE), trichloroethene (TCE), cis-dichloroethene (cDCE), trans-dichloroethene (tDCE), and 1,1-dichloroethene (1,1-DCE), as well as vinyl chloride (VC), form a group of volatile organic compounds (VOCs) that have traditionally been used as solvents in various industrial applications (Pankow and Cherry 1996). Primarily, they have been used as degreasers in cleaning and degreasing applications (Rivett et al. 2014; Pankow and Cherry 1996). These are carcinogenic compounds, whose toxicity has been extensively studied by Hsu (2018), Guha et al. (2012), and Huijbregts et al. (2005), among others. Their presence in subsurface materials and in groundwater and surface water represents a significant risk to human health and ecosystems (Chen et al. 2017; Hartwell, 2000).

Their behavior in the medium is governed by their physical and chemical characteristics, especially their higher density in the pure state compared to freshwater (Moran et al. 2007), with the exception of VC (Seddon et al. 2000). These compounds are therefore classified as dense non-aqueous phase liquids (DNAPLs). Their migration as a free phase through the subsurface is predominantly regulated by the force of gravity and capillarity (Zheng et al. 2015), and the relative mobility of this free phase with respect to freshwater is determined by the density/viscosity ratio of the DNAPL and water (Nsir et al. 2018; Pan et al. 2016; Zheng et al. 2015). Chloroethenes, as hydrophobic compounds, exhibit varying degrees of sorption by the soil or sediment organic carbon fraction (*f*_oc_), exerting control over the mass flux transferred from the aqueous phase to the solid phase (Morrison and Murphy 2015). To this, the mass flux due to molecular diffusion must be added, which results in the penetration of these contaminants into the low-hydraulic-conductivity layers. Both mechanisms are responsible for the total contaminant mass flow from groundwater flowing through high-hydraulic-conductivity levels to low-hydraulic-conductivity levels, such as silts and clays characterized by high organic matter (OM) contents. These levels can become real reservoirs in which chloroethenes accumulate significantly, either by sorption and molecular diffusion mechanisms or by being deposited as free phase pools on these low-conductivity levels.

These reservoirs are sources of groundwater contamination by chloroethenes; they may emit these contaminants from the free phase in the early stages of pollution episodes, desorb the mass retained in the OM, or back-diffuse the mass stored in low-conductivity layers when, in the final stages of the episode, the pools are already in a residual form or even in more advanced stages. This explains to a large extent the recalcitrance of these pollution sources, especially in mature sites, where the back-diffusive flux maintains the concentrations of these compounds above the reference values established in the different regulations (Chapman et al. 2005). Pankow and Cherry (1996) describe the main physicochemical characteristics of these compounds and their behavior in different hydrogeological environments. The existence of important heterogeneities in the subsurface materials significantly controls their distribution as free phase and as dissolved phase (Puigserver et al. 2016; Wu et al. 2017; Rivett et al. 2014; Erning et al. 2012).

The hyporheic zone (HZ) of a river comprises the shallow benthic sediments of the riverbed, the most superficial segment of the aquifer hydraulically connected to the mentioned sediments, and the portion of the banks closest to the river (Cárdenas 2015; Boano et al. 2014; Krause et al. 2011; Woessner 2000; Boulton et al. 1998). The transition between the HZ and the aquifer is, in many cases, heterogeneous, showing a wide range of hydraulic conductivities and different water residence times (Gómez-Vélez et al. 2014; Sawyer and Cárdenas, 2009; Haggerty et al. 2002). The remarkable heterogeneity of the HZ, with frequent textural changes, favors the occurrence of alternating fine and coarse materials, making this zone an important ecotone. In addition, the fluvial sediments of this zone are characterized by a significant OM content, propitiating optimal microbial development (Wehncke and Mariano 2021).

The OM input to the hyporheic environment is mostly allochthonous, mainly coming from the ground surface (tree leaves and woody debris) and from soil washing (especially from the floodplain). OM can also derive from river water in the downwelling parts of the HZ, where it can accumulate in the sediment. To a lesser extent, OM may be contributed by groundwater reaching the hyporheic environment in places where vertical upwelling occurs. The upwelling and downwelling fluxes, in turn, regulate the vertical fluctuation of the oxic-anoxic interface in the HZ (Hampton et al. 2020), favoring the presence of facultative microorganisms capable of degrading multiple contaminants (Freixa et al. 2016). In fact, this interface controls the discharge of pollution plumes between groundwater and surface water bodies (Weatherill et al. 2018; Simsir et al. 2017; Sonne et al. 2018; Puigserver et al. 2014; McKnight et al. 2012; Conant J.B. 2004). This interface is characterized by being located in a heterogeneous and dynamic zone that is biochemically very active, where groundwater and surface water meet, exchange, and mix, even in the case in which the riverbank is influent (Engelhardt et al. 2011). This generates important inputs of OM (as a source of carbon and energy) and oxygen, creating strong gradients in the oxidation–reduction potential (ORP) (Larned et al. 2015). This favors the existence of redox zonation spatially and in depth following the model described by the electron tower (Wagner et al. 2019). A distinctive feature of the HZ is the presence of zones under methanogenic conditions driven by the occurrence of archaea. This promotes the formation of anoxic microzones with ORP values lower than those of the redox zone corresponding to sulfate reduction, even in oxygenated sediments. In these anoxic microzones, it is common to find microorganisms capable of utilizing Fe^2+^ and Mn^2+^, H_2_, or reduced sulfur compounds, which act as electron donors under anoxic conditions. In addition, since iron and manganese oxidation produce little energy and are performed primarily by heterotrophic bacteria, these ions contribute minimally to hyporheic productivity, except when OM is at extremely low concentrations and those of the metals are high (Storey et al. 1999).

The HZ is of great environmental interest, as it behaves as a natural bioreactor (Boano et al. 2014) that should not be saturated, since it is home to microorganisms capable of biodegrading a large number of pollutants of diffuse origin (such as nitrogen species) or emerging pollutants resulting from agricultural and livestock activities, as well as industrial and urban activities (Höhne et al. 2022). In recent years, much attention has been focused on the potential of the HZ as a mitigator of chlorinated solvent pollution (Simsir et al. 2017; Atashgahi et al. 2016; Freitas et al. 2015; Weatherill et al. 2014).

Reductive dechlorination (the process by which chlorine atoms in chloroethenes are sequentially replaced by hydrogen atoms) is a key anaerobic degradation pathway occurring across a range of redox conditions in contaminated aquifers. This process represents a form of anaerobic respiration primarily mediated by organohalide-respiring bacteria (OHRB), in which PCE is transformed stepwise into progressively less chlorinated compounds, ultimately yielding ethene or ethane (Jugder et al. 2016).

However, complete mineralization is rarely achieved under environmental conditions, and partial reductive dechlorination commonly dominates. This limitation is closely associated with the decreasing energy yield as chlorination decreases, together with constraints in electron donor availability and incomplete or insufficiently specialized microbial communities (Vogel and McCarty 1985; Bouwer, 1994; Wei and Finneran 2011). As a consequence, intermediate products such as cDCE and VC frequently accumulate in aquifers, where their degradation is kinetically and microbiologically more constrained (Haston and Mccarty 1999; Puigserver et al. 2016). Given that both compounds exhibit higher toxicity than their parent compounds, their accumulation represents a significant risk to human health and ecosystems (Dolinová et al. 2017). In addition, the persistence of these intermediates reflects a major limitation of natural attenuation based solely on reductive dechlorination (Maymó-Gatell et al. 1997).

Chloroethene degradation in subsurface environments is strongly controlled by redox zonation, which governs both the thermodynamic feasibility and the dominant microbial processes. In natural systems, spatial and temporal heterogeneity in redox conditions often limits complete dechlorination and promotes the accumulation of intermediate products. Reductive dechlorination proceeds along a typical redox cascade, with PCE transformation to TCE occurring under denitrifying conditions, followed by conversion to cDCE under Mn- and Fe-reducing conditions, further reduction to VC under sulfate-reducing conditions, and final conversion to ethene or ethane under methanogenic conditions (Bradley 2003; Chapelle 1996; Amaral et al. 2011; Antoniou et al. 2019; Aulenta et al. 2007; Němeček et al. 2020; Weatherill et al. 2019). However, in most field settings, redox conditions are not stable or sufficiently reducing, which restricts the progression to complete mineralization and favors partial reductive dechlorination (Bradley and Chapelle 2011; Maymó-Gatell et al. 2001).

A further limitation arises from the availability of electron donors, particularly molecular hydrogen (H₂), which is the main energy source for OHRB. Competition for H₂ with other anaerobic microbial groups, including nitrate-, manganese-, iron-, and sulfate-reducing bacteria, methanogens, and homoacetogens, can significantly constrain reductive dechlorination efficiency (Wei and Finneran 2011). Moreover, elevated chloroethene concentrations may inhibit microbial activity and reduce community diversity, thereby promoting specialization within the microbial community (National Research Council 1999).

Microbial reductive dechlorination is mediated by a broad range of genera, including *Desulfitobacterium*,* Clostridium*,* Dehalobacter*,* Sulfurospirillum*, *Geobacter*,* Desulfomonile*,* Desulfovibrio*, and* Dehalogenimonas* (Kim et al. 2006; Atashgahi et al. 2016; Nijenhuis and Kuntze 2016). However, only specific strains within the genus *Dehalococcoides*, particularly *Dehalococcoides mccartyi*, are capable of complete dechlorination of PCE to ethene (Maymó-Gatell et al. 1997; Löffler et al. 2013). These organisms are therefore widely used as biomarkers of active organohalide respiration. In contrast, many other OHRB are unable to dechlorinate beyond cDCE or VC, leading to accumulation of these toxic intermediates (Sung et al., 2003). At the enzymatic level, reductive dechlorination is catalyzed by reductive dehalogenases (RDases), encoded by genes such as rdhA, tceA, vcrA, and bvcA, which exhibit strong substrate specificity and differing activity across dechlorination steps (Hug et al. 2013; Payne et al. 2014; Lihl et al. 2019). This enzymatic constraint further contributes to the prevalence of partial reductive dechlorination in environmental systems.

Under oxic or mildly oxidizing conditions, chloroethene degradation follows alternative oxidative pathways, which are particularly relevant for the transformation of lower chlorinated intermediates such as cDCE and VC (Findlay et al. 2016; Field and Sierra-Alvarez, 2001; Lohner and Tiehm 2009). Oxidative degradation may proceed through two main mechanisms: electron transfer-driven processes and hydroxyl radical-mediated reactions. In electron transfer-based systems, the simultaneous availability of hydrogen and oxygen can stimulate coupled reductive and oxidative transformations, enhancing overall chloroethene removal efficiency (Li et al. 2023). This coupling has been demonstrated in engineered and in situ systems, where increased oxygen availability enhances degradation rates and promotes sequential removal of chlorinated ethenes (Lohner et al., 2011; Hertle et al. 2023).

In parallel, hydroxyl radical-driven oxidation plays a significant role under aerobic or redox-fluctuating conditions. The generation of hydroxyl radicals is strongly dependent on dissolved oxygen availability, and their formation has been shown to directly control chloroethene oxidation rates (Pham et al. 2009; Schaefer et al. 2018; Li et al. 2023). Coupled biotic–abiotic systems, such as those involving iron-cycling bacteria (e.g., *Shewanella oneidensis*), can further enhance oxidative degradation through Fenton-like reactions, where biogenic Fe(II) and hydrogen peroxide react to produce highly reactive hydroxyl radicals (Sekar et al. 2016; You et al. 2023).

At the microbial level, aerobic degradation of cDCE and VC occurs via both co-metabolic and direct metabolic pathways. Co-metabolic oxidation involves non-specific oxygenases, such as monooxygenases and dioxygenases, which are expressed during growth on primary substrates (e.g., methane, ethane, propane, toluene, or phenol) and fortuitously oxidize chloroethenes into reactive intermediates such as epoxides (Bradley 2003; Mattes et al. 2010; Chi et al. 2023). Methanotrophic bacteria play a central role in this process through methane monooxygenases (MMOs), with soluble MMOs generally exhibiting higher co-metabolic efficiency than particulate forms (Anderson and Mccarty 1997). These processes typically occur at plume fringes, where oxygen and co-substrates coexist (Bradley and Chapelle 2000; Broholm et al. 2005).

In addition, several aerobic microorganisms are capable of direct oxidation of cDCE and VC as sole carbon and energy sources. Reported genera include *Mycobacterium*,* Nocardioides*,* Pseudomonas*,* Ralstonia*, and *Polaromonas* (Coleman et al. 2002; Danko et al. 2004; Taylor et al. 2007). In the case of VC, initial oxidation is catalyzed by alkene monooxygenase (AkMO), producing chlorooxirane, which is subsequently detoxified by epoxyalkane: coenzyme M transferase (EaCoMT) and metabolized into central metabolic pathways (Coleman and Spain 2003; Mattes et al. 2010). For cDCE, degradation is less common, but has been observed in strains such as *Polaromonas* sp. JS666, likely involving epoxidation and glutathione S-transferase-mediated pathways (Coleman et al. 2002; Jennings et al. 2009).

Overall, oxidative dechlorination provides a crucial complementary pathway to reductive processes, particularly in redox transition zones. In these environments, oxidative mechanisms contribute to the removal of toxic intermediates such as cDCE and VC that accumulate during incomplete reductive dechlorination, thereby enhancing the overall efficiency of chloroethene natural attenuation.

The abiotic degradation of chloroethenes has been described in natural contexts, especially in the presence of Fe minerals such as pyrite, troilite, mackinawite, vivianite (Bae and Lee 2012), magnetite (Culpepper et al. 2018), Fe^2+^ sorbed to iron oxides, Fe^2+^-containing clay minerals, and even biotite (He et al. 2015) and green rust (Fan et al. 2023; He et al. 2015). The interdependence between abiotic and biotic processes may favor a coupling between the two processes and can lead to an increased rate of degradation of chlorinated solvents, including chloroethenes (Berns et al. 2019). In addition, metabolic products resulting from degradation caused by biotic reactions can facilitate abiotic reactions at the field scale, resulting in coupled biotic and abiotic processes. For example, acetylene can generate acetate, ethanol, and ethane through microbial fermentation (Miller et al. 2013) and can also be used as an electron donor in microbial sulfate reduction, leading to FeS precipitation (Butler et al. 2013). Research by authors such as Tobiszewski and Namiesnik (2012) suggests that abiotic dechlorination is generally slower than microbial degradation. Despite this, the interdependence of coupled abiotic and biotic processes can generate synergies between these processes, which may favor a higher degradation rate of chlorinated solvents, including chloroethenes (Berns et al. 2019; Patterson et al. 2016; Bradley and Chapelle 2010). However, the relevance of the degradation rate in these cases depends on the abundance and nature of the microorganisms at the contaminated site and the type of minerals involved, e.g., state II iron minerals tend to favor higher degradation rates (Butler et al. 2013). Microbial activity depends on the access of microorganisms to electron donors, i.e., the availability of electrons, so that they can obtain the energy they need (Scheutz et al. 2010). The predominant electron donor in reductive dechlorination is molecular H_2_ (Holliger and Schumacher 1994). In natural attenuation, hydrogen formation is attributed to the fermenting community in the subsurface, which uses natural OM for energy (McCarty and Smith 1986), so this OM controls reductive activity in aquifers. Isotopic enrichment varies depending on the compound, the enzymatic biodegradation pathway (Chartrand et al. 2005), geochemical parameters (such as redox conditions), and the development of microbial populations or abiotic reactions. This progressive enrichment is expressed by the Rayleigh equation (Steinbach et al. 2004; Mariotti et al. 1981).

The main objectives of this work are the following: (i) to define the control exerted by the geological characteristics of the subsurface on the distribution of dechlorinating microbial communities in the HZ; (ii) to study the preferential flow and transport pathways of chloroethenes in this zone; (iii) to analyze the coupling between biotic oxidative and reductive dechlorinating processes; and (iv) to investigate the isotopic fractionation of chloroethenes as a function of the geological characteristics of the subsurface.

The working hypothesis postulates that the geological structure of fluvial systems and their HZ control the preferential flow and degradation pathways of chloroethenes, favoring the complete mineralization of these pollutants.

**Fig. 1:**
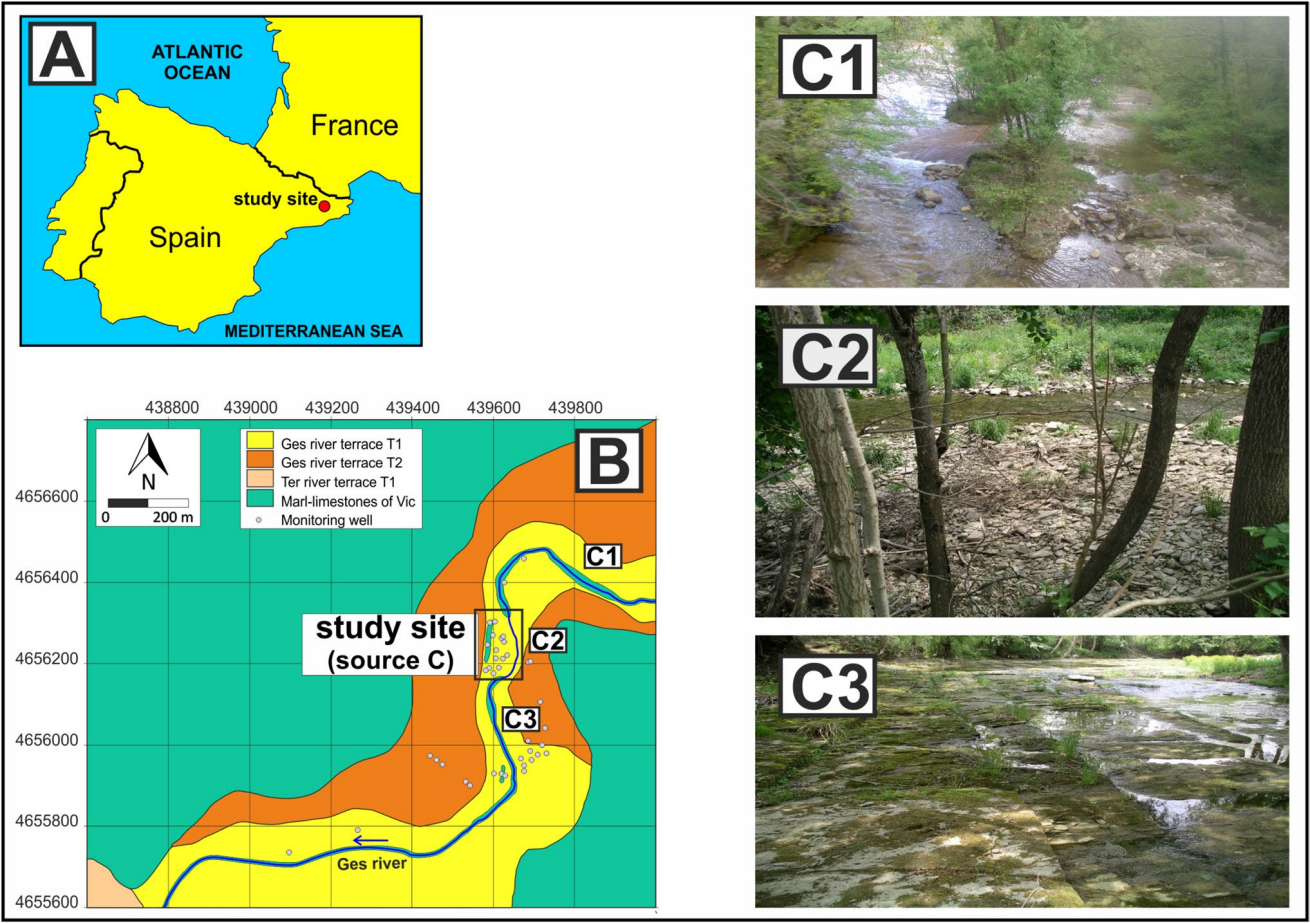
(A) Geographical situation of the site where sediments and groundwater were collected for the treatability test. (B) Geological context of the study site (source C). (C) Pictures upstream (C1), in the source C (C2) and downstream (C3) in the study site

To test the validity of this hypothesis, a field site was selected for the research to be conducted in. The site is located approximately 85 km northwest of Barcelona (see Fig. 1 A). It is contaminated by PCE (DNAPL source C in Fig. 1B), and this contamination affects a Quaternary alluvial aquifer hydraulically connected to a river and a fractured marlstone aquifer of Eocene age. These rocks are fractured by joints, which, along with predominantly horizontal stratification, form a highly penetrating orthogonal system of fractures. The geological and hydrogeological characteristics of the aquifers affected by the contamination are described in Puigserver et al. (2014).

## Materials and methods

### Groundwater monitoring networks

For the purpose of monitoring groundwater chloroethene concentrations and isotope fractionation (as well as redox-sensitive compounds in groundwater: nitrate, manganese, iron, and sulfate) at this contaminated site, 14 of the monitoring piezometers from the control network of the Catalan Water Agency (ACA) were utilized. These are conventional piezometers screened from the geological contact of the Quaternary detritic materials at the base of the alluvial aquifer to the phreatic surface. The saturated thickness (*H*_0_) normally varies little (between 10 and 12 m). These conventional piezometers were designed specifically for sampling groundwater from different aquifer units and for measuring piezometric levels (refer to the locations of these piezometers in Figs. 1 and 5, in which pie charts of chloroethenes molar fractions and total concentration are centered on these piezometers).

### Sampling and analyses of water and sediments

Soil and sediment samples from research boreholes S1, S2, S3, S5, S6, S9, S10, and S11 (drilled by our research group), as well as groundwater from these boreholes (once they were equipped as monitoring piezometers), along with groundwater from the monitoring network of the ACA and surface water from the Ges River, were collected during the spring 2022 sampling survey. Table 1 compiles the key parameters measured in the water, soil, and sediment samples and the analyses performed.

**Table 1:** Parameters measured and analyses performed. *EC*, electrical conductivity; *DO*, dissolved oxygen; *ORP*, oxidation–reduction potential; *OC*, organic carbon; *TOC*, total organic carbon

Sample type				Sample type			
	**Groundwater**	**Surface water**	**Soil and sediment**		**Groundwater**	**Surface water**	**Soil and sediment**
**Grain size analysis**	**-**	**-**	**X**	**TOC in water (mg/L)**	**X**	**X**	**-**
**Water depth (m)**	**X**	**-**	**-**	**Redox-sensitive species in water (mg/L)**	**X**	**X**	**-**
**T (°C)**	**X**	**X**	**-**	**Total Fe in soil and sediments (mg/kg)**	**-**	**-**	**X**
**EC (µS/cm)**	**X**	**X**	**X**	**Total Mn in soil and sediments (mg/kg)**	**-**	**-**	**X**
**DO (mg/L)**	**X**	**X**	**-**	**Chloroethenes in water (µg/L)**	**X**	**X**	**-**
**ORP (mV)**	**X**	**X**	**X**	**δ**^**13**^**C‰ of chloroethenes in water**	**X**	**X**	**-**
**pH**	**X**	**X**	**X**	**Microorganisms in water**	**X**	**X**	**-**
**OC in soil and sediment (mg/kg)**	**-**	**-**	**X**				

The sampling protocols for the hydrochemical, isotopic, and microbial characterization (phyla and genera) of the surface water and groundwater, as well as soils and sediments, followed the procedures described by Puigserver et al. (2023, 2014).

All samples were analyzed by the Environmental Hydrogeology and Global Change Group (affiliated with the Sustainable Biotechnology and Bioremediation Group of the University of Barcelona, UB) in the laboratories of the Scientific and Technological Centers of the UB (CCiTUB), which adhere to ISO 9001:2000. The main analytical techniques used are listed in Table 2.

**Table 2:** Main analytical techniques and protocols applied

Analysis	Protocol	Technique
Grain size analysis		
OC in soil and sediment (mg/kg)	ISO 10694:1995	Gas chromatography with thermal conductivity detector (TCD)
TOC in water (mg/L)		Using a TOC-5000 analyzer (Shimadzu)
Redox-sensitive species in water (mg/L)	EPA 9056 and EPA Method 200.8	High-performance liquid chromatography (HPLC) and inductively coupled plasma-mass spectrometry (ICP-MS), Elan 6000, Perkin Elmer, Waltham, MA, USA
Total Fe in soil and sediments (mg/kg)	Pretreatment to determine metals consisted in extracting them from a selected fine fraction of the sample with *aqua regia.* ISO/DIS 11466:1995 protocol	Inductively coupled plasma optical emission spectroscopy (ICP-OES)
Total Mn in soil and sediments (mg/kg)		
Chloroethenes in water (µg/L)	Puigserver et al. (2016)	Gas chromatography (GC, Carlo-Erba GC8000-Top) coupled to a mass spectrometer (GC–MS, Thermo Finnigan Fisons MD800)
δ^13^C‰ of chloroethenes in water	Puigserver et al. (2016)	Gas chromatography combustion isotope ratio mass spectrometry (GC–C–IRMS)
Microorganisms in water	Willis et al. (2018) and Puigserver et al. (2023)	16S rRNA gene sequencing by Illumina MiSeq with 2 × 300 bp reads

#### Processing of analytical data obtained

The granulometric and geochemical data of the total organic carbon (TOC), Fe, and Mn in soil and sediments were analyzed in conjunction with physicochemical parameters to characterize redox zones in depth, similar to the approach employed by Puigserver et al. (2022).

From the analytical results of the water, spatial distribution maps of redox-sensitive species were generated along the HZ and in the adjacent part of the aquifer. Redox zones were defined, and distribution maps of chloroethenes and their isotopic composition were generated. Their evolution was assessed along a profile parallel to the river and along transects in the direction of flow. Additionally, distribution maps of microbial genera associated with redox processes and chloroethene degradation were developed, together with their evolution along the mentioned profile and transects.

In the case of microbial data, a bioinformatic analysis was performed on raw forward and reverse sequences, which underwent quality filtering using FastQC (r). Subsequently, reads were pre-processed using Prinseq (Schmieder and Edwards 2011), following the specifications outlined by Piazzon et al. (2019).

Taxonomy assignment utilized the Ribosomal Database Project (RDP) release 11, encompassing both Archaea and Bacteria, as the reference database (Cole et al. 2014). Alignment and taxonomy assignment were conducted using the GPRO Suite software (Futami et al. 2011), following the methodology described by Piazzon et al. (2019).

## Results and discussion

### Geological and hydrogeological control of the aquifer-hyporheic zone system on the distribution of contaminants and their biodegrading microorganisms

The geological and hydrogeological structure of the HZ and the hydraulically connected aquifer system exert a primary control on the distribution of contaminants and their biodegrading microorganisms. In particular, the attenuation and degradation potential of chloroethenes is governed by a combination of key site-specific characteristics, including (i) sedimentological heterogeneity (paleochannels versus interchannel deposits); (ii) organic matter content and associated sorption capacity; (iii) groundwater flow patterns and residence times; and (iv) the coupling between river–aquifer interactions and redox zonation. These factors are described below based on field observations and measurements.

The geological and hydrogeological structure of the HZ and the aquifer system hydraulically connected to it significantly influence the distribution of contaminants and microorganisms capable of degrading them in the study site. In the map in Fig. 1B (at the point labeled C1 and downstream of point C2), it can be observed that the main channel of the Ges River flows over fractured Eocene marlstones (see photos C1 and C3 in Fig. 1). The orthogonal fracture system affecting these marlstones (see photo C3 in Fig. 1) controls the discharge from the aquifer associated with these marlstones to the river, as the piezometric level of this aquifer unit was situated above the water level of the river. In these reaches, where the riverbed is formed by Eocene marlstones, currently sedimentation is practically absent, although the presence of transported blocks deposited during extreme flood events can be observed.

**Fig. 2:**
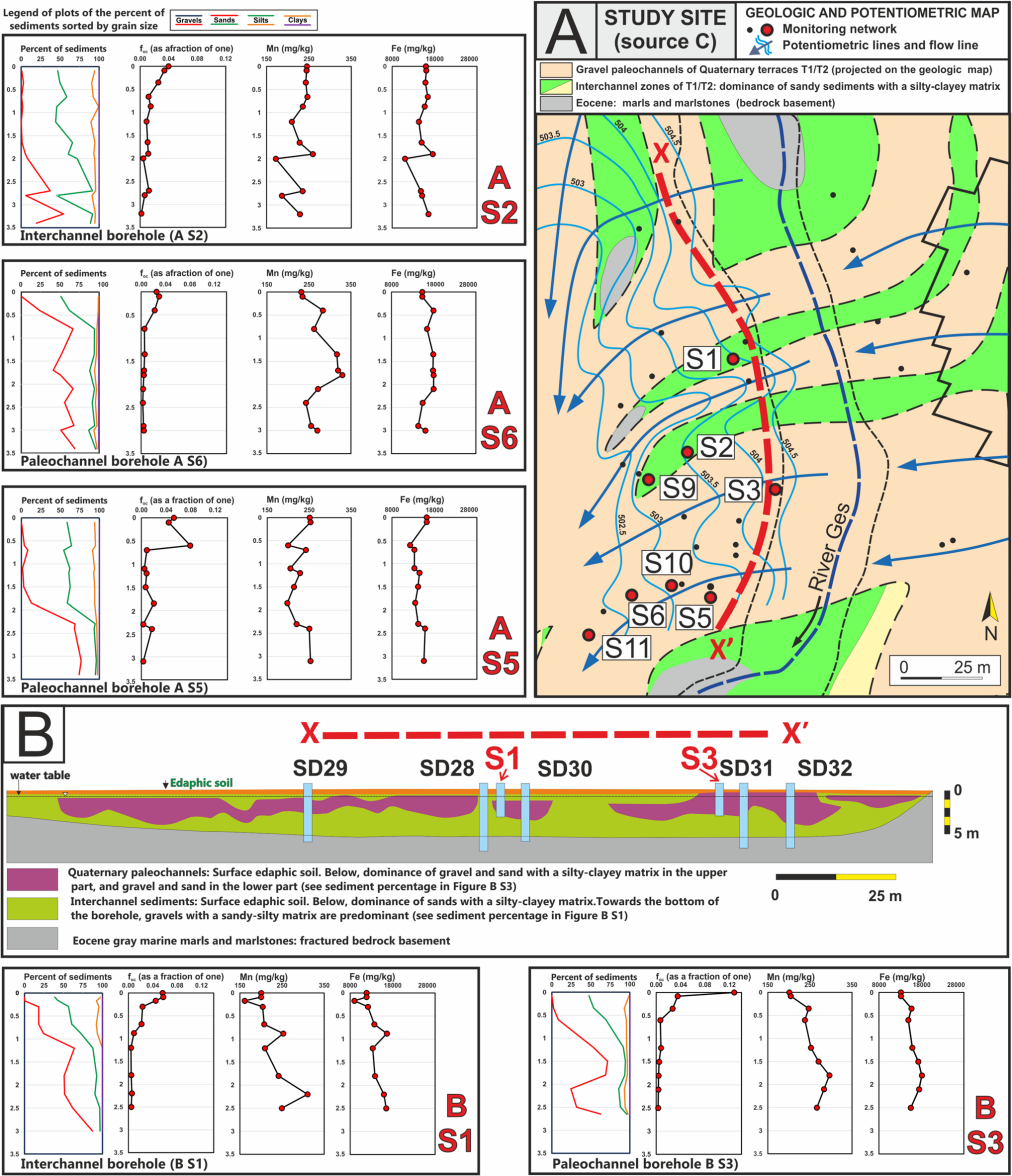
Geological, geochemical, and hydrogeological characteristics of the study site. Spring 2022 sampling survey. (A) Geological and potentiometric map displaying indicative groundwater flow lines: S2, S6, and S5 represent three exploration boreholes to determine variations, both in depth and along the flow, of the following parameters: sediment percentage by grain size (granulometry), organic carbon fraction (*f*_oc_, as a fraction of one), concentration of Mn and Fe (both in mg/kg). (B) Graphical representation of the vertical section of the profile indicated as X-X′ on the geological map (profile conducted parallel to the flow of surface water in the Ges River from upstream to downstream of the river). This vertical section corresponds to the HZ in the right bank, and results from the integration of subsurface data obtained from electrical tomography field surveys, and exploration borehole surveys for sediment sampling (boreholes S1, S3) in which the determined parameters were the same as in the mentioned boreholes (S2, S6, and S5). Stratigraphic descriptions of monitoring piezometers of the ACA and exploration boreholes indicated in the vertical section X-X′ (SD29, SD28, SD30, SD31, SD32, S1 and S3), along with borehole S5, were also used as parametric boreholes for the interpretation of the electrical tomography profile

In contrast, in the stretch facing source C of DNAPL PCE (labeled C2 on the map in Fig. 1B), the river flows over Quaternary material deposits from fluvial terraces, where sedimentation-erosion can also occur in the shallowest part of the HZ. These deposits correspond to paleochannels (see photo C2 in Fig. 1) and interchannel areas, representing floodplain sediments. Most of the paleochannels formed a set of amalgamated channels spreading laterally (Sharma et al. 2023), although some paleochannels were isolated. The thicknesses of these paleochannels (both amalgamated and isolated) ranged from 1 to 4 m (see profile X-X′ and its location below ground in the right natural levee of the Ges River). Gravels and sands dominated in the paleochannels, while interchannel areas were characterized by sands with a silty matrix and silts with a clayey matrix, typical of floodplain deposits. The thalweg lines of the paleochannels were slightly inclined towards the west in the HZ, crossing under the riverbed from the left bank to the right bank, where the river becomes influent (Fig. 2 A). The corresponding input of surface water from the river gave rise, beyond the levee area of the right bank, to a broad propagation of the effect of the HZ to the aquifer below the floodplain on the right bank (similarly to what Peterson et al. (2016) described in a groundwater flow simulation of an influent river). Related to the situation of the propagation of the effect of the HZ, significant variations in the redox conditions of the aquifer occurred in the stretch of paleochannels on the right bank (see the “Groundwater redox zonation of the aquifer-hyporheic zone system” section) and in the occurring biogeochemical reactions, including those involving the natural attenuation of chloroethenes.

In the piezometric map in Fig. 2 A, it is observed that, once on the right bank, the flow turns south. In this stretch, where the river flows over the paleochannels, both on the right bank forming part of the HZ (see profile X-X′ in Fig. 2 A) and in the rest of the HZ (located beneath the riverbed up to the left bank), Quaternary sediments extend to a depth of 5 m. Below this depth, fractured marlstones were found (refer to the location of profile X-X′ in the map of Fig. 2 A and to the vertical section in Fig. 2B). In the vertical section X-X′, the Quaternary sediments corresponding to the paleochannels presented fining-upward sequences in which, although there may be some pebbles and even cobblestones at the bottom, coarse gravels dominated at depth, gradually decreasing to fine sand at the top. Floodplain deposits also existed, formed by interbedded levels of variable thickness, which, although they may contain some intercalation of fine-grained matrix gravels, were primarily composed of fine sediments of a silty-clayey character. These fine sediments exhibited a high particulate OM content, especially in the upper part of the profile, which was more edaphized (up to the first 0.3- or 0.7-m deep). This abundant OM in the more edaphized upper part mainly came from river floods, riparian vegetation, and fauna remnants from the bank. Their maximum concentrations, expressed as organic carbon (OC) in the form of *f*_oc_ as a fraction of one, varied between 0.057 and 0.128 in boreholes S1 and S3, respectively (Fig. 2B). In contrast, below the edaphized soil, in these same boreholes, *f*_oc_ values gradually decreased to values ranging from 0.003 to 0.005 at a depth of 2.5 m, i.e., below the water table on the right bank of the HZ, where, as mentioned, the river was influent (Fig. 2 A, B). The presence of particulate OM associated with the fine fraction not only enhances sorption processes but also promotes the development of microbial communities involved in biodegradation, as coarse grain sizes hinder biofilm formation (Romani et al. 2016). This highlights the indirect but critical role of sediment texture and OM in controlling biodegradation potential.

Downgradient of the vertical profile X-X′ on the right bank, where the river became influent (Fig. 2 A, C), *f*_oc_ values in sediment samples taken below the water table (located approximately 0.8 m below the ground surface) progressively decreased as the distance in the aquifer from the right bank increased, similarly to what was described by Galia et al. (2022). Thus, the average *f*_oc_ values below the water table along the same flow line in an interchannel area varied from 0.0088 in borehole S2 to 0.0085 in borehole S9, or in borehole S1, which, with an *f*_oc_ value of 0.0064, was located in a different interchannel area. The locations of these boreholes are shown on the map in Fig. 2A. Similarly, along a flow line in a paleochannel area, such as the one passing through boreholes S5, S10, S6, and S11, values ranged from 0.0054 to 0.0043 (respectively, in S5 and S11). The locations of all these boreholes are shown on the map in Fig. 2 A, and the *f*_oc_ values in the mentioned boreholes are provided in Table SI–1 in the supporting information document (SI).

Similar to the decrease in *f*_oc_ values just described, the same boreholes show that in the saturated zone, there was an increase in the percentage of fine sediments as the groundwater moved further within the right bank of the river. This occurs because the river is influent on this bank (Fig. 2 A, C). Thus, near the river, the input of surface waters leads to a washing of fine sediments, resulting in an increase in the hydraulic conductivity of the medium close to the river (similar to what was observed by Geng et al. 2021). In this area, hydraulic conductivity values fluctuated between 65 and 315 m/day, with average values of 85 m/day and 305 m/day for silty-dominant materials and gravel-dominant materials, respectively. Table SI–2 in the SI provides the hydraulic conductivity values determined by field slug tests in the monitoring network of the ACA. Hydraulic conductivity values, together with the variability in thicknesses and depths observed in the paleochannel area on the right bank, which is shown in the vertical section X-X′ of Fig. 2 A, B, explain the variations in transmissivity along this section (transmissivity values varied from 600 to 1100 m^2^/day in the gravel materials). Deeper paleochannels presented higher hydraulic conductivity values than shallower ones. Additionally, within the same paleochannel, fining-upward sequences led to a lower effective porosity and hydraulic conductivity towards the top (Bellizia et al. 2021). Additionally, as mentioned earlier, the thalweg lines of the paleochannels were inclined towards the west on the right bank, although they later turn towards the south. The consequence of this was that the main directions of groundwater flow in the aquifer of Quaternary alluvial materials were conditioned by the direction of these thalweg lines (map in Fig. 2 A), so that the paleochannels act as draining lines (Puigserver et al. 2014). Geological and sedimentological characteristics, OM content, sorption properties, and groundwater velocity (with linear average velocities ranging from 3.5 to 50 m/day for paleochannel areas and from 2.5 to 6 m/day for interchannel areas) jointly act as key controlling factors on the residence time and, therefore, on the natural attenuation potencial of chloroethenes at site. For example, for the molar fraction of PCE dissolved in groundwater from pools in the HZ that is still not degraded, the longest residence time occurred in interchannel areas, where the retardation was higher than in paleochannel areas due to higher sorption because of the amount of particulate OM (higher values of *f*_oc_) in the interchannels (similar to what was observed by Bonsor et al. 2017).

Thus, the residence time of PCE from the HZ on the right bank of the river to a location a distance of 35 m downgradient towards the interior of the aquifer along the interchannel area corresponding to transect D-D′ was on average 50 days. In contrast, for the same distance from the HZ along a paleochannel, such as that corresponding to transect F-F′, the residence time for PCE was much shorter (averaging only 12 days). The lower occurrence of particulate OM (see the *f*_oc_ values in Fig. 2 and in Table SI–1 in the SI) and the higher linear average velocity of groundwater account for the shorter residence time of PCE, in a manner consistent with the observations of Dhivert et al. (2015).

### Groundwater redox zonation of the aquifer-hyporheic zone system

The redox zonation observed in the aquifer–HZ system constitutes a key control on the natural attenuation and biodegradation potential of chloroethenes, as it determines the availability of electron acceptors and donors that regulate microbially mediated degradation pathways.

**Fig. 3:**
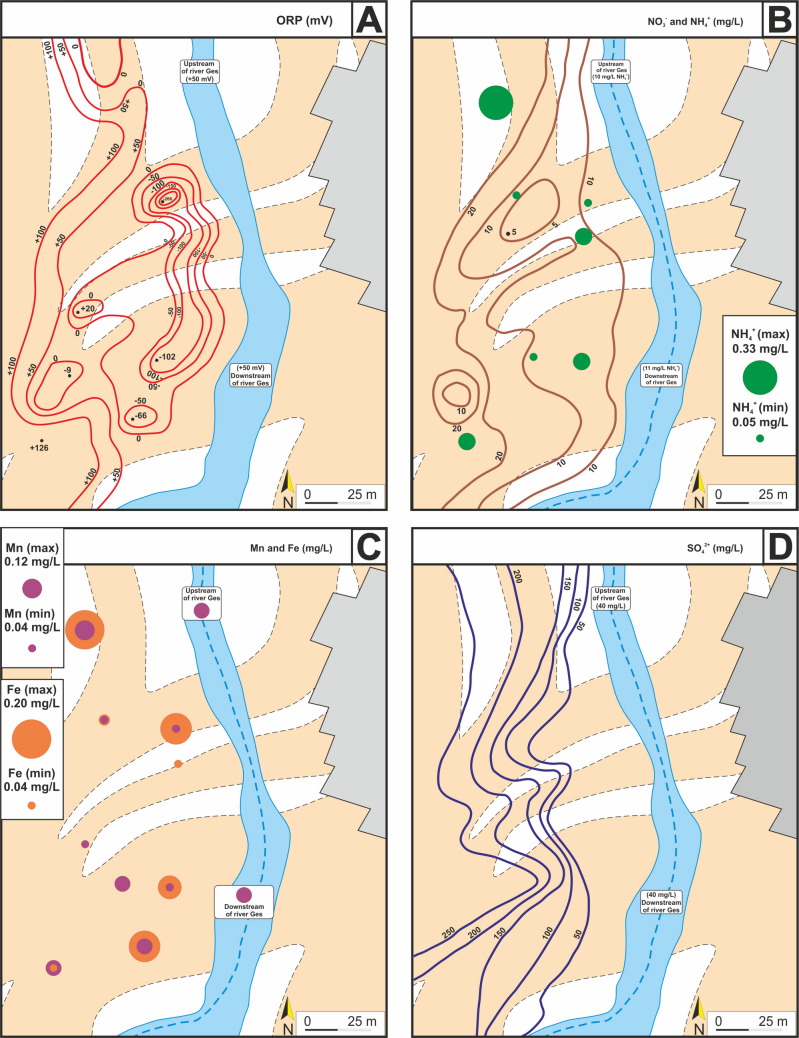
Spring 2022 sampling survey. Maps of ORP values (mV), ammonium, nitrate, manganese, iron, and sulfate (concentrations mg/L) in groundwater. Values of these parameters in the surface water of the Ges River at two points located to the N and S of the study site are also included. Background color codes: beige (paleochannel areas); white (interchannel areas); light blue (Ges riverbed); gray (factory buildings). (A) Contours map of ORP (mV). (B) Contours map of nitrate. Map of ammonium (green circles). (C) Maps of manganese and iron (purple and brown circles, respectively). Iron was not detected in surface water. (D) Contours map of sulfate

On the right bank of the river, the presence of particulate OM (expressed in terms of *f*_oc_), deposited during periods of river flooding and also derived from riparian vegetation in the HZ adjacent to the right bank of the river (see profile X-X′ in Fig. 2 A, B), promoted the rapid consumption of oxygen from the inflow of surface water (similar to observations by Mueller et al. 2021). This resulted in significant gradients of dissolved oxygen (DO) reduction along the flow lines on the right bank. These gradients were more pronounced in the interchannel areas, where the higher presence of fine sediments (silt and clays) accompanied by a large amount of OM (i.e., higher *f*_oc_ values) favored reducing conditions and elevated gradients of DO decrease. Thus, DO concentrations varied from 12.2 mg/L at borehole S2 to 0.22 mg/L at borehole S9 (both boreholes equipped as piezometers). These boreholes were separated by 15 m along the same flow line (Fig. 2B), resulting in a gradient of decreasing DO of 0.80 mg/L per m along the flow line passing through these boreholes. In contrast, in the paleochannel areas, DO decreasing gradients were lower due to the higher flow velocity and penetration of surface water into the right bank along these paleochannels (Puigserver et al. 2014), with a DO decrease gradient of 0.33 mg/L per m along the flow line passing through boreholes S5, S10, S6, and S11 (with a distance of 37 m between S5 and S11; all these boreholes were equipped as piezometers). This distribution of the DO concentration along the alluvial aquifer resulted in a heterogeneous redox zonation. Thus, the most reducing ORP values (Fig. 3 A) on the right bank (− 266 and − 102 mV occurred at S1 and S3, respectively; both boreholes were equipped as piezometers) were found in the area closest to the river, coinciding with high *f*_oc_ values (0.06 at borehole S1 and 0.128 at S3; see Fig. 2B). Close to the river, ammonium concentrations of up to 0.15 mg/L (Fig. 3B) occurred; the oxidation of this ammonium by DO (leading to nitrate) contributed to the consumption of oxygen (similar to observations by Yan et al. 2022).

The redox conditions at the study site were also influenced by point-source contaminations resulting from wastewater discharges originating from poultry farms located in the northwest sector of the study site (Fig. 2 A). In this sector, the ORP values were clearly reducing (between − 50 and − 20 mV) or close to slightly reducing values (between 2 and 0 mV) (Fig. 3 A). The ammonium concentration in this sector was very high, reaching 0.33 mg/L, which is significantly higher than concentrations recorded downgradient and in the rest of the aquifer on the right bank of the river (which do not exceed 0.15 mg/L; see Fig. 3B).

The distribution of redox-sensitive species allowed the identification of dominant redox zones, which are directly linked to specific microbially mediated degradation pathways (e.g., denitrification, Fe- and sulfate-reduction, and methanogenesis), thus defining the spatial variability of biodegradation potential across the site similar to observations by O’Connor et al. (2015).

**Fig. 4:**
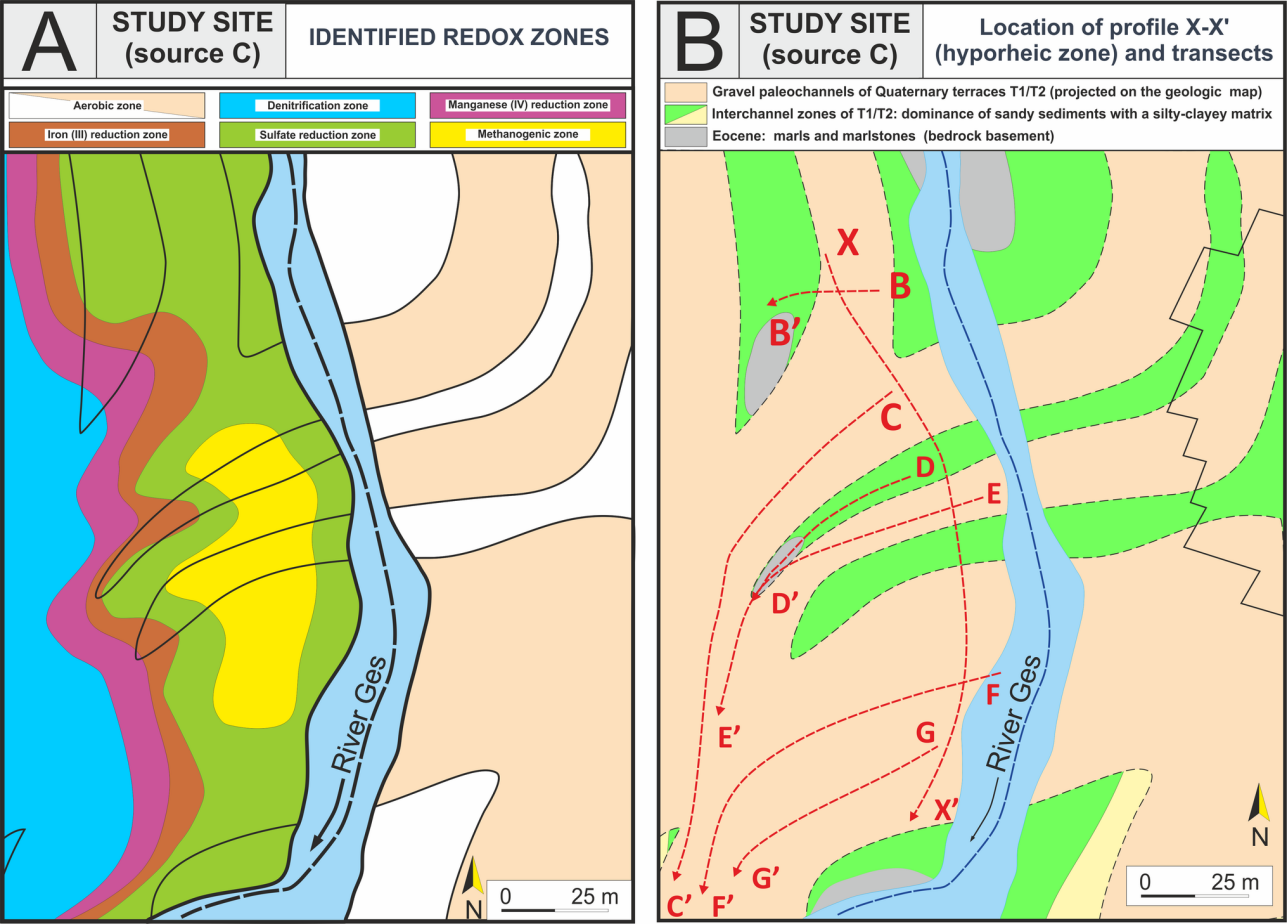
Redox zones identified at the study site. Spring 2022 sampling survey

In the HZ of the right riverbank, the presence of high concentrations of OM favored the development of redox conditions close to those of reduction, explaining the low nitrate concentrations (Fig. 3B), which are even below those recorded in surface waters. These low nitrate concentrations can be explained by the following: the combination of (i) groundwater dilution effects in the aquifer (Puigserver et al. 2014) due to the influent nature of the river (water table map of Fig. 2 A), especially in the paleochannel areas, and (ii) negative ORP values (due to DO consumption), which promote denitrification processes (when nitrate concentrations are high), but especially anaerobic ammonium oxidation (ANAMMOX) processes, according to Aguilar-Rangel et al. (2020). The progressive increase in nitrate concentrations (Fig. 3B) towards the innermost areas of the aquifer and away from the river (map in Fig. 2 A) is a consequence of the continuous input of nitrate derived from diffuse contamination in the region (Gros et al. 2021), caused by the widespread use of manure as fertilizer in agricultural crop fields (Puigserver et al. 2014). Figure 4 shows how the redox zone corresponding to nitrate reduction was situated in the innermost areas of the aquifer, precisely where nitrate concentrations were higher and conditions were less reducing (similar to observations by Tesoriero et al. 2021).

The presence of Fe- and Mn-bearing minerals can also contribute to sorption processes, as well as provide redox-active phases that can act as electron acceptors, indirectly promoting microbial reductive dechlorination processes. The Fe and Mn redox transitions are not only indicators of redox zonation but actively control microbial metabolism, as Fe (III)- and Mn (IV)-reducing conditions are commonly associated with anaerobic degradation pathways, including reductive dechlorination of chloroethenes.

The highest concentrations of Mn^2+^ in groundwater (up to 0.12 mg/L) were mainly found in the innermost areas of the aquifer (Fig. 3 C), where animal manure had been locally stored. As for the maximum concentrations of Fe^2+^ (up to 0.2 mg/L), these were recorded in the zones closest to the river (Fig. 3 C) on the right bank, which is part of the HZ, coinciding with most reducing conditions (with ORP values ranging from − 66 to − 266 mV); this is consistent with redox zones corresponding to sulfate reduction (Fig. 3D) and methanogenesis.

The fact that, in these zones closest to the river, the dominant redox conditions were higher than those of Fe reduction and the Fe^2+^ concentrations were elevated is consistent with the previously mentioned dilution effect due to the influent nature of the river (Puigserver et al. 2014) on the right bank, which is part of the HZ (Fig. 2B). This dilution effect diminished the sulfate concentration in groundwater (Fig. 3D), decreasing its bioavailability and degradation rate through sulfate-reduction processes, accompanied by the precipitation of iron sulfide (pyrite). This led to a decrease in Fe^2+^ in groundwater, similar to observations by Puigserver et al. (2023) and Einsied et al. (2015).

The decrease in Fe^2+^ and sulfate due to sulfide precipitation was also observed in areas where the aforementioned point-source contamination occurred due to wastewater discharges (northwestern of the study site; see Fig. 2 A). In this sector, redox conditions ranged from sulfate-reducing to methanogenic, accompanied by sulfate inputs that enabled sulfate reduction to occur (according to the isotopic fractionation of sulfate in that sector of the study site observed by Puigserver et al. 2014). The coexistence of Fe reduction, sulfate reduction, and methanogenesis suggests overlapping redox niches that favor the development of organohalide-respiring microorganisms.

Figure 4 graphically represents the distribution of the identified redox zones. The figure illustrates how in interchannel areas, Fe-reduction and sulfate-reduction conditions dominated, while in paleochannel areas, Mn-reduction conditions and denitrification prevailed. These redox gradients are initially driven by hydrogeological and geological controls (e.g., surface water inflow, sediment texture, and organic matter distribution), but are progressively enhanced by microbial activity through the consumption of electron acceptors and the production of reduced species.

Overall, the combination of organic matter availability, groundwater flow patterns, and the resulting redox zonation defines the spatial distribution of favorable conditions for chloroethene biodegradation, with the most reducing zones near the river and in fine-grained interchannel deposits showing the highest attenuation potential.

### Chloroethene distribution and ^13^C isotopic fractionation along the aquifer-hyporheic zone system

The spatial distribution of chloroethenes and their carbon isotopic fractionation provide direct evidence of the key factors controlling natural attenuation and biodegradation processes at the study site. These include (i) geological heterogeneity controlling DNAPL distribution; (ii) sorption and organic matter content affecting contaminant retardation and bioavailability; (iii) groundwater flow and mixing processes regulating dilution and electron acceptor availability; and (iv) redox-dependent microbial activity driving reductive dechlorination.

The distribution of chloroethene concentrations on the right bank of the river in the area of paleochannels was the result of the past free-phase migration of DNAPL PCE through the paleochannels and interchannels from an initial free phase source of contamination located on the left bank of the river (primary source). A part of this free phase moved and accumulated as pools on the right bank (secondary source), which constituted the source C in Fig. 1B, especially in the area closest to the river, which is a part of the bank belonging to the HZ (profile X-X′ in Fig. 2 A, B). This is the area with the major pools on clayey and silty layers of low hydraulic conductivity interstratified in paleochannels and especially in the interchannel areas (Puigserver et al. 2014). Similarly, some other pools accumulated further into the bank. The ensemble of all the above-mentioned pools resulted in source C (Fig. 2 A).

Currently, the highest concentrations of chloroethenes are found in this zone, in the interchannel areas along profile X-X′ belonging to the HZ (Fig. 2B). PCE concentrations in groundwater in the source area showed initial concentrations above 10 mg/L (60.3 μmol/L; Puigserver et al. 2014). These high values significantly decreased in the present study (spring 2022 sampling survey) in the paleochannel areas, with concentrations below 0.1003 mg/L (0.60401 μmol/L); this represents a decrease of up to 99.7% with respect to the initial concentrations in groundwater. In contrast, in interchannel areas where residual DNAPL PCE still exists, concentrations of around 1 mg/L (6.03 μmol/L) were detected in groundwater, indicating lower rates of DNAPL dissolution in these areas (similar to observations in transition zones between aquifers and aquitards by Puigserver et al. 2023). Sorption by particulate OM is higher in interchannel areas, leading to increased retardation and reduced contaminant mobility, which in turn affects the bioavailability of PCE for microbial, similarly to what was observed by Puigserver et al. (2016).

**Fig. 5:**
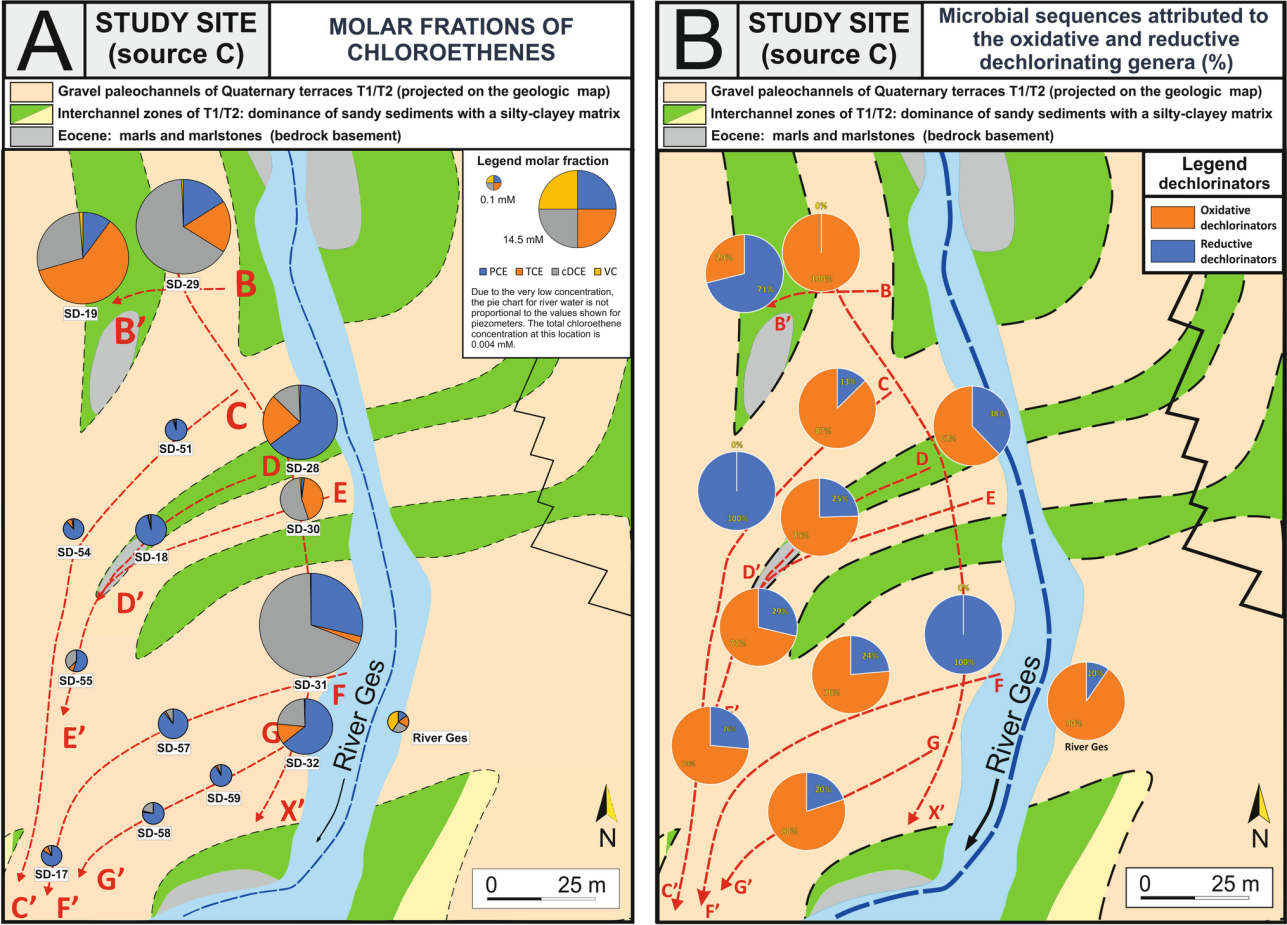
(**A**) Pie charts of molar fractions of chloroethenes in the study site. These molar fractions have been represented along the X-X′ profile on the right bank (as part of the HZ of the Ges River); piezometers involved are SD29, SD28, SD30, SD31, and SD32. Molar fractions have also been represented along the following transects: B-B′ (SD29; SD19, following groundwater flow lines through paleochannel and interchannel areas); C–C′ (SD51; SD54; SD55; SD17, following a flow line along a paleochannel); D-D′ (SD28; SD18, following an interchannel area); E-E′ (SD30; SD55; SD17, following a flow line along a paleochannel); F-F′ (SD31; SD57; SD17, following a flow line along a paleochannel); and G-G′ (SD32; SD59; SD58; SD17, following a flow line along a paleochannel). Spring 2022 sampling survey

**Fig. 6:**
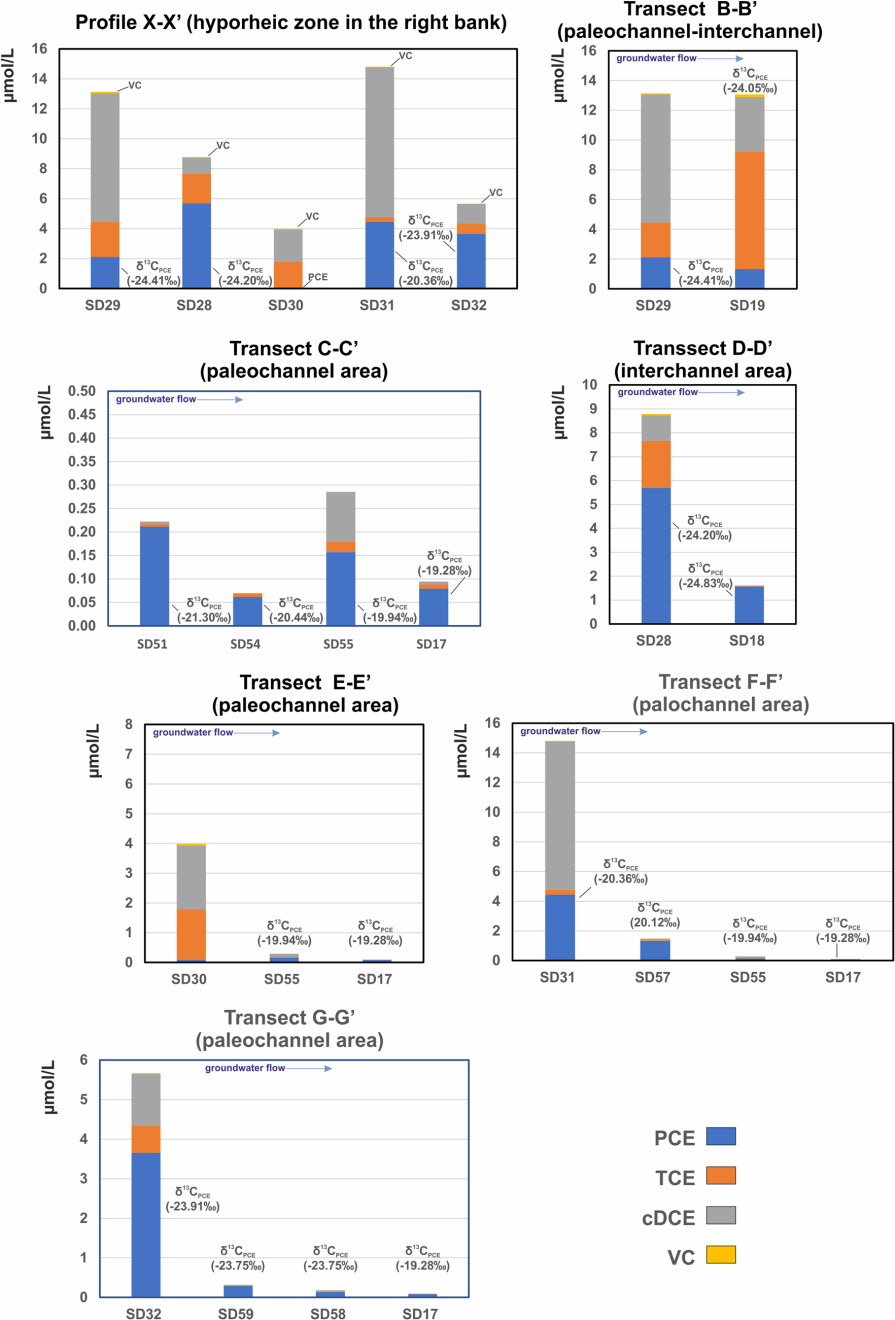
Spatial evolution of molar concentrations and isotopic fractionation of PCE (δ.^13^C values) in profile X-X′ (as part of the HZ of the Ges River); piezometers involved are SD29, SD28, SD30, SD31, and SD32. Also shown are the molar concentrations and isotopic fractionation of PCE in transects: B-B′ (SD29; SD19, following groundwater flow lines through paleochannel and interchannel areas); C–C′ (SD51; SD54; SD 55; SD17, following a flow line along a paleochannel); D-D′ (SD28; SD18, following an interchannel area); E-E′ (SD30; SD55; SD17, following a flow line along a paleochannel); F-F′ (SD31; SD57; SD55; SD17); and G-G′ (SD32; SD59; SD58; SD17, following a flow line along a paleochannel)

The observed ^13^C isotopic enrichment of PCE provides clear evidence of in situ biodegradation processes, enabling the identification of zones with higher degradation potential. This is supported by the significant isotopic fractionation exhibited by dissolved PCE relative to the original DNAPL PCE (δ^13^C = − 25.66‰ in its residual phase; Puigserver et al. 2014). In particular, in some areas of the right bank within the HZ (profile X-X′ in Fig. 2 A, B), dissolved PCE became considerably enriched, reaching δ^13^C values of up to − 20.36‰ (Fig. 6, piezometer SD31), which coincides with zones showing high abundances of reductive dechlorinating microorganisms (Fig. 5B).

The X-X′ profile in Fig. 2 A is also represented in Fig. 5 A, which illustrates that the higher molar concentrations of dissolved PCE were located in the interchannel areas, accompanied by metabolites of PCE (the parent compound), similar to observations by Weatherill et al. (2018). It is precisely in the right bank (HZ, profile X-X′) where the highest degradation rates of PCE and the highest molar fractions of cDCE and VC occurred (Phillips et al. 2022), as shown in Fig. 5A.

(B) Pie charts of microorganisms capable of oxidative and reductive dechlorination along the groundwater flow. Each segment of the pie chart represents the percentage of microbial sequences (relative to the total identified in the sampled groundwater) attributed to microorganisms of the dechlorinating genera. Spring 2022 sampling survey.

Various pieces of evidence indicate that reductive dechlorination in this part of the HZ was more pronounced than in other parts of the contamination source: (i) the elevated presence of particulate OM in this HZ area (Fig. 2); (ii) the clearly methanogenic redox conditions (Fig. 4); and (iii) cDCE with a lighter isotopic composition (with a δ^13^C_cDCE_ value of − 24.37‰) compared to the isotopic composition of PCE dissolving and degrading in the HZ, which ranged from − 24.41 to − 20.36‰ (Fig. 6, profile X-X′).

In this part of the HZ, the subsurface geological structure and contaminant degradation control the mobilization rate of the PCE source. Thus, in the paleochannel areas, for example, along transect F-F′, the molar concentrations of chloroethenes provided by piezometer SD31 were higher than those observed in transect G-G′ by piezometer SD32 (Fig. 6). This is because along the latter paleochannel, there is a greater proportion of surface water mixing with groundwater, given the larger dimensions of this paleochannel and its higher hydraulic conductivity (see the information on hydraulic conductivity obtained through field tests in Table SI–2 in the SI). Additionally, it was observed that, although the degradation of dissolved PCE occurred in both F-F′ and G-G′, it was incomplete, resulting in the accumulation of cDCE (and only very little VC), as redox conditions varied from Fe reduction to sulfate reduction (similarly to what was observed by Němeček et al. 2020). In these two transects, there was uneven isotopic fractionation of PCE; it was higher in F-F′ (heavier) than in G-G′ (lighter; see Fig. 6), coinciding with more reducing conditions in F-F′ (Fig. 4 A, B), which is consistent with greater cDCE accumulation in F-F′ (Fig. 6).

As mentioned at the beginning of this section, the part of the right bank belonging to the HZ (profile X-X′) is where the free-phase DNAPL PCE was placed in the past. It is in this part of the HZ where the highest concentrations of chloroethenes were detected. However, it is also where the molar fractions of PCE metabolites (TCE and cDCE) were the highest, which agrees with the fact that this is a source that, while it was already aged in 2014 (Puigserver et al. 2014), is still active.

In transect B-B′ (NW sector of the study site), the presence of high amounts of dissolved OM from a continuous point source of contamination (Puigserver et al. 2014), stemming from the inadequate management of the liquid waste of livestock and poultry, accounts for the Fe-reducing and sulfate-reducing redox conditions observed. These redox conditions, particularly Fe-reducing environments, are known to enhance microbial reductive dechlorination, as Fe cycling can facilitate electron transfer processes required for chloroethene transformation.

Figure 3 C shows the high concentration of dissolved Fe recorded in this sector of the study site, and the spatial distribution of different redox zones can be observed in Fig. 4. According to the Fe-reducing redox conditions, the presence of TCE (with a δ^13^C value of − 23.29‰, much heavier than that of PCE, which in this area had a δ^13^C value of − 24.41‰; see Fig. 6, piezometer SD29) and the accumulation of cDCE (Vogel et al. 2018) showed a high degradation rate of TCE. Along transect B-B′, the rate of metabolite production was higher than in the other transects due to the higher percentage of OM, as mentioned earlier.

Along the flow lines, differences in the evolution of chloroethene concentrations were observed. Thus, in transects following the paleochannels of large dimensions, E-E′, F-F′, and G-G′, the last characterized, as mentioned earlier, by being situated along an area where the dimensions of the paleochannel are larger, the input of surface water from the river promoted a higher rate of dissolution of residual DNAPL PCE (Koch and Nowak 2015). In turn, this water input led to a greater dilution effect (Epting et al. 2018) than in transect E-E′. This led to a decrease in concentrations of redox-sensitive species such as nitrate (Fig. 3B), reducing competition between denitrifying and dechlorinating microorganisms in favor of the latter and enhancing chloroethene degradation.

In addition, the input of surface water from the river not only promotes DNAPL dissolution but also alters the availability of electron acceptors (e.g., nitrate), further supporting reductive dechlorination processes.

In the area where different paleochannels converge and where the mixing effect with surface waters is already less significant, it was observed that the predominant molar fraction is PCE (see in the map with the pie charts in Fig. 5 A and the plot of piezometer SD17 in the SW sector of the map). Furthermore, chloroethene molar concentrations had already significantly decreased in this sector (Fig. 6). Nevertheless, along the flow, the progressive degradation of dissolved PCE was observed, with δ^13^C_PCE_ values becoming heavier along the flow (see Fig. 6).

Along the larger paleochannels (E-E′, F-F′, and G-G′), it was observed that the molar concentrations of the metabolites of PCE decreased (Fig. 6), as well as the molar fractions of these metabolites (Fig. 5 A). This highlights the lower degradation rate occurring along the flow within these paleochannels (Puigserver et al. 2014).

In transects following smaller paleochannels, such as C–C′, a progressive increase in dissolved PCE degradation was indeed observed, as it became heavier along the flow (reaching a δ^13^C value of up to − 20.44‰; see Fig. 6, piezometer SD54), and the cDCE concentration increased along the flow (Fig. 5 A), especially in sectors where sulfate-reducing conditions were recorded (see Fig. 4 and piezometer SD55 in Fig. 6).

Conversely, in interchannel areas, the decrease in chloroethene concentrations along a flow line such as that followed by transect D-D′ was much lower than in paleochannel areas (Fig. 6). This was because dilution by surface water input was lower than in paleochannels. A greater production of metabolites along the flow was observed, although the presence of aged pools formed by residual DNAPL along the transect increased the molar fraction of PCE in groundwater, as was the case for piezometer SD18 (Fig. 5 A, Fig. 6).

### Distribution of microorganisms in the subsurface

The distribution of microorganisms in the HZ is controlled by a set of key site-specific factors that directly determine the biodegradation potential of chloroethenes. These include (i) redox conditions and electron acceptor availability; (ii) organic matter content as a source of electron donors; (iii) hydrogeological controls such as groundwater flow and river–aquifer interactions; and (iv) geological heterogeneity (paleochannel versus interchannel structures), which define the spatial distribution of microbial niches.

The distribution of microorganisms in the HZ is primarily controlled by redox conditions, which are governed by the interplay between geological structure, hydrogeology, and organic matter (OM) distribution. These factors collectively regulate the availability of electron donors and acceptors, thereby determining the dominant microbial metabolisms and overall biodegradation potential. Importantly, redox conditions not only control microbial distribution but are also shaped by microbial metabolism, resulting in a dynamic feedback between physical controls and biogeochemical processes.

Figure SI–1 in the SI shows how the Bacteria domain predominated in the surface waters of the river and in the paleochannel areas where oxidizing conditions predominated. In contrast, the Archaea domain increased its presence (in percentage terms of the sequences assigned) in the interchannel areas (transect D-D′), which coincided with the more reducing redox conditions (Fig. 4), similarly to what was observed by Zuo et al. (2022).

Table 3 shows the distribution of the most abundant bacterial phyla. Among these, the most abundant were *Proteobacteria* (with a percentage of 12.72% of the sequences assigned to this phylum) and *Actinobacteria* (8.34%), a phylum related to OM decomposition (Zhu et al. 2020).

**Table 3:** Distribution of the most abundant bacterial phyla in the study site. Bacterial phylum to oxidative (OD/od) and reductive (RD/rd) dichlorination

Phyla	Percentage of sequences assigned to a phylum	Contribution to dechlorination
Proteobacteria (Fe, Mn, and SO_4_^2−^ reduction)	12.72	RD
Acidobacteria	8.34	od
Planctomycetes	5.65	od
Chloroflexi	4.49	RD
Parcubacteria	2.60	rd
Woesearchaeota	1.96	rd
Cyanobacteria/chloroplast	1.35	OD
Nitrospirae (nitrification)	1.32	od
Thaumarchaeota (Archeobacterial domain)	1.16	od
Absconditabacterales (formerly known as SR1)	1.14	Rd

### Genera involved in redox process occurring in the aquifer-hyporheic zone system and factors governing their distribution and development

#### Spatial distribution of the genera of microorganisms capable of oxidize organic matter, nitrifying, and denitrifying

The spatial variability in microbial communities also reflects hydrogeological controls, as the inflow of surface water through paleochannels promotes more oxidizing conditions, whereas interchannel areas remain more reducing due to lower flow velocities and higher OM content.

The spatial distribution of OM-oxidizing, nitrifier, and denitrifier microorganisms (Figure SI–2 and Figure SI–3 in the SI) showed a higher proportion of denitrifiers in the HZ portion of the right bank adjacent to the river, i.e., along profile X-X′. This aligns with the more reducing conditions recorded in that profile compared to the innermost zone of the aquifer on the right bank (see the “Groundwater redox zonation of the aquifer-hyporheic zone system” section), where (similarly to observations by Azizian et al. 2017) the proportion of OM oxidizers and nitrifiers increased (Soldatova et al. 2021). This is related to diffuse agricultural pollution from manure fertilization in the region (see the “Groundwater redox zonation of the aquifer-hyporheic zone system” section) and specifically in the aquifer zone (similarly to observations by Morrissy et al. 2022). This pollution ensures a continuous input of OM, which through ammonification promotes high concentrations of ammonium (Lee et al. 2006), which in turn is oxidized by nitrifying microorganisms (Buss et al. 2004).

This resulted in an increase in nitrate concentrations (Fig. 3B), which promoted the development of nitrifying and denitrifying communities, highlighting how nutrient inputs and redox conditions jointly regulate microbial processes relevant to contaminant attenuation. In addition, the spatial variability in microbial communities reflects hydrogeological controls, as the inflow of surface water through paleochannels promotes more oxidizing conditions, whereas interchannel areas remain more reducing due to lower flow velocities and higher OM content. Furthermore, the elevated OM content in fine-grained interchannel deposits not only enhances sorption but also influences contaminant bioavailability, thereby indirectly controlling microbial degradation rates.

In the case of transect B-B′, the increase in the proportion of OM-oxidizing microorganisms was particularly significant, in line with a higher input of OM associated with wastewater discharge from the improper management of the liquid waste of livestock and poultry (see the “Chloroethene distribution and ^13^C isotopic fractionation along the aquifer-hyporheic zone system” section). Along transects C–C′, E-E′, and F-F′ in the paleochannel areas, a progressive increase in OM-oxidizing microorganisms and nitrifiers was observed. In contrast, in interchannel areas (transect D-D′), the proportions of denitrifiers were higher than those observed in the paleochannel areas. Additionally, these proportions in the interchannel areas remained constant along the flow, which is consistent with the predominance of more reducing conditions (see Figs. 3 and 4). The dilution effect (section “Groundwater redox zonation of the aquifer-hyporheic zone system”) produced by the influent relationship between the river and aquifer (Puigserver et al. 2014) was also reflected in the microbial distribution, as the higher proportion of surface water penetrating into the aquifer through the paleochannel areas (Fig. 2B, transects C–C′ and F-F′) ensured more oxidizing conditions than in interchannel areas, favoring a progressive increase in OM-oxidizing microorganisms and nitrifiers inward along the aquifer through the paleochannels.

The aerobic organotrophic microorganisms identified in surface water, which play a crucial role in the aerobic degradation of OM in water (see Figure SI–2 and Figure SI–3 in the SI) belong to the *Litorilinea* genus, which exhibits aerobic growth and is chemoorganotrophic (Kale et al. 2013), and the *Turneriella* genus, which is obligately aerobic (Levett et al. 2005). These microorganisms were not detected in the HZ (profile X-X′) or the interchannel areas, which is consistent with these areas presenting more reducing conditions (see the “Groundwater redox zonation of the aquifer-hyporheic zone system” section and Fig. 4).

In the paleochannel of transect C–C′ and to a lesser extent E-E′ and F-F′, the microorganisms identified in surface waters were present, along with *Polynucleobacter*. This is a bacterioplankton genus isolated from freshwater that is capable of degrading dissolved OM (Watanabe et al. 2009). Other genera of microorganisms identified were *Caulobacter*, bacterial organoheterotrophs capable of degrading OM in different media (including water) and associated with polyaromatic hydrocarbons (Wilhelm et al. 2018), and *Gordonia*, a strict aerobic OM degrader associated with hydrocarbon-contaminated sites (Franzetti et al. 2009) and with the degradation of petroleum hydrocarbons and polycyclic aromatic hydrocarbons (Drzyzga 2012). This genus is also known to be capable of nitrate reduction (Kim et al. 2003). Other identified microorganisms belonged to the *Fluviicola* genus (although with a low microbial load), which consists of aerobic or strictly aerobic species usually associated with oxygen consumption (O’Sullivan et al. 2005).

The most abundant nitrifying microorganisms (see Figure SI–2 and Figure SI–3 in the SI) found in surface water were *Nitrolancea*, which is a Gram-positive nitrite oxidizer genus capable of growing with nitrite and formate (Sorokin et al. 2014), and *Zavarzinella*, which is acidophilic and associated with nitrification–denitrification processes (Fuerst 2017). *Pseudomonas* was also present; it is capable of simultaneously completing nitrification and denitrification processes, in contrast to the traditional relatively independent aerobic nitrification and anaerobic/anoxic denitrification processes (Gao et al. 2020). These processes illustrate the coupling between hydrogeological transport of electron acceptors and their subsequent consumption by microbial communities.

*Diaphorobacter*, which is capable of carrying out simultaneous processes of nitrification and denitrification under aerobic conditions (Khardenavis et al. 2007), as well as degrading organic compounds such as polycyclic aromatic hydrocarbons (PAHs) (Wang et al. 2020), phenol (Ge et al. 2015), and triclocarban (Liang et al. 2020), was another genus identified in surface water.

To a lesser extent, *Nitrosospira*, an ammonia-oxidizing bacterium (Lourenço et al. 2018; Taylor and Bottomley 2006; Norton et al. 1996), which can also produce N_2_O (Shaw et al. 2006) and grow under aerobic and anaerobic conditions (Shaw et al. 2006), was also identified in surface water. On the other hand, *Limnohabitans*, with a significant role in freshwater bacterioplankton communities due to their high rates of substrate uptake, is capable of degrading OM (Stegen et al. 2016) and participating in the degradation of nitrate (Lee et al. 2021). In the part of the HZ corresponding to profile X-X′, these microorganisms were not detected, while in the more interior area of the right bank, the most abundant genera were *Pseudomonas*, *Diaphorobacter*, *Nitrosospira*, and *Nitrosomonas*, which clearly contribute to nitrification (Krüger et al. 2023). This is consistent with the more oxidizing conditions of these inner parts of the aquifer, as well as with the diffuse contamination of the aquifer by manure application.

The most abundant denitrifying microorganisms detected in surface waters were *Gemmatimonas*, which, although they are not able to reduce nitrate to nitrite (Zeng et al. 2015; Zhang et al. 2003), do exhibit NO_2_^−^ and N_2_O reductases, which can decrease N_2_O emissions and completely mineralize NO_3_^−^ (Chee-Sanford et al. 2019). *Gaiella* and *Conexibacter* (capable of reducing nitrate to nitrite) were also identified in river water (Severino et al. 2019; Albuquerque et al. 2011; Monciardini et al. 2003). *Phycisphaera*, capable of reducing nitrate to nitrite (Fukunaga et al. 2009), *Emticicia*, which was also found to be able to denitrify nitrate and nitrite (Xie et al. 2016; Liu et al. 2008; Saha and Chakrabarti 2006), and *Luteolibacter*, which is able to reduce nitrate to nitrite exclusively under aerobic conditions (Yoon et al. 2008), were identified, along with *Flavobacterium* to a lesser extent (described as anaerobic denitrifiers capable of reducing NO_3_^−^ to dinitrogen gas; Horn et al. 2005). In the river water, *Pseudomonas* and *Acidovorax*, able to reduce nitrate under aerobic conditions (Willems et al. 1992); *Acidovorax* sp. KKS102 (Shehu and Alias 2019), *Aquabacterium*, capable of using nitrate as an electron acceptor and a nitrate-dependent ferrous (Fe^2+^) oxidizing strain under anaerobic conditions (Xu et al. 2022); *Truepera*, which is related to nitrite denitrification and metal reduction in an Fe-reducing environment (Whittleston et al. 2013); *Cellvibrio*, able to reduce nitrate to nitrite under aerobic conditions (Mergaert et al. 2003); *Schlesneria*, a facultative aerobic nitrate reducer (Kulichevskaya et al. 2007); *Zavarzinella*, a chemo-organotrophic aerobe and an assimilatory microorganism that uses nitrate and ammonium found in wetland areas (Kulichevskaya et al. 2009); *Prosthecobacter*, a facultative aerobic and anaerobic microorganism capable of nitrate reduction (Lee et al. 2014; Takeda et al. 2008); *Roseomonas*, a strict aerobic capable of nitrate reduction (Sánchez-Porro et al. 2009); and *Hydrogenophaga*, capable of degrading nitrate (Fan et al. 2023), were also identified.

In the HZ (profile X-X′), in interchannel areas (transect D-D′, where an aged pool still remains; see piezometer SD18 in Figs. 5 and 6), and in transect B-B′, where an input of OM was detected (see the “Chloroethene distribution and ^13^C isotopic fractionation along the aquifer-hyporheic zone system” section), the most reducing conditions controlled the microbial distribution, since in these areas the redox conditions were more reducing than those of the nitrate reduction (Fig. 4). Thus, the low microbial loads of *Conexibacter*, *Gaiella*, *Gemmatimonas*, *Phycisphaera*, *Emticicia*, and to a lesser extent *Patulibacter* (not identified in surface waters and capable of nitrate reduction; Saha and Chakrabarti 2006; Takahashi et al. 2006) were identified. In contrast, along the paleochannel areas, there was a progressive increase in *Conexibacter*, *Gaiella*, and *Gemmatimonas* (also present in surface waters). Meanwhile, the presence and evolution along the flow in the aquifer of other microorganisms present in surface waters were uneven.

The transect along the paleochannel C–C′ exhibits a higher increase in *Flavobacterium*, *Pseudomonas*, *Acidovorax*, *Aquabacterium*, *Phycisphaera*, *Emticicia*, and *Diaphorobacter* (the latter promotes the coupling of nitrification and denitrification reactions under aerobic conditions). This profile is situated further inside the aquifer, with groundwater showing a lower percentage of mixing with surface water from the river. In contrast, for the transect along the paleochannel F-F′, the influx of surface water promotes more oxidizing conditions, resulting in a lower proportion of denitrifying microorganisms.

### Spatial distribution of the genera of Mn-oxidizing, Fe-oxidizing, and Fe-reducing microorganisms

The distribution of Fe- and Mn-cycling microorganisms is particularly relevant, as these elements play a direct role in controlling redox conditions and indirectly regulate microbial reductive dechlorination processes.

The occurrence of Mn-oxidizing, Fe-oxidizing, and Fe-reducing microorganisms (Figure SI–4 and Figure SI–5 in the SI) coincided with the spatial distribution of Fe in groundwater in the area close to the river on the right bank, where the amount of Fe was higher than in the inner parts of the aquifer (Fig. 3 A, C). This is consistent with a greater reduction rate of Fe^3+^ to Fe^2+^ (Figs. 3 and 4); see Tesoriero et al. (2017) and McMahon and Chapelle (2008).

In the paleochannel areas, the input of surface water favored the presence of Mn and Fe oxidizers, although this is also attributable to their presence in the groundwater of the aquifer. The higher proportion of Fe oxidizers inside the aquifer agrees with a lower proportion of Fe in the groundwater of that part of the aquifer (Fig. 3 C) compared to the HZ (profile X-X′, Figs. 2 and 3).

In surface waters, *Pseudolabrys*, an aerobic genus capable of oxidizing Mn (Dangeti et al. 2020), and *Delftia*, which is also capable of oxidizing Mn, although its ability to degrade hydrocarbons has also been observed (Hong et al. 2023), were identified. These microorganisms were also detected in the internal areas of the paleochannels, where Mn-reduction conditions prevailed (Fig. 3 C, Fig. 4, and Fig. 2 A, transect C–C′).

Fe oxidizers were not detected in surface water but were present in the internal areas of the paleochannels, coinciding with the areas where Mn oxidizers were identified. Among the identified microorganisms in transects C–C′ and F-F′, *Ferrovibrio*, clearly an Fe oxidizer (Ryu et al. 2020), and, with a lower microbial load, *Azospira*, capable of Fe oxidation and also denitrification (Puigserver et al. 2016, 2023; Li et al. 2016), can be mentioned.

The microbial diversity of Fe-reducing microorganisms was greater than that of oxidizers. *Rhodofera* (capable of reducing Fe and also degrading TCE; Luo et al. 2019), *Pseudomonas*, and *Aquabacterium* were detected in surface waters. The presence of Fe reducers was not detected in the HZ (profile X-X′) and interchannel areas, coinciding with redox conditions that were more reducing than Fe reduction, whereas their presence was detected in paleochannel areas, especially in smaller ones, such as that corresponding to the C–C′ transect.

In addition to the increasing microbial load of microorganisms present in surface waters, this study identified several Fe-reducing genera, including *Aciditerrimonas* (a facultative anaerobe capable of reducing Fe^3^⁺; Itoh et al. 2011), *Ferribacterium* (an Fe reducer also involved in the reduction of other metals; Cummings et al. 1999; Jroundi et al. 2020), *Melioribacter* (Fortney et al. 2018), and *Geobacter* (Reguera and Kashefi 2019; Lovley et al. 1993), which has been widely reported as capable of degrading chlorinated solvents (Dutta et al. 2022). Additionally, *Geothrix*, a microorganism clearly involved in the Fe cycle (Xue et al. 2021), was detected at lower abundance, particularly in localized areas such as that represented by piezometer SD55 (transect E-E′), where punctual organic matter inputs promoted more reducing conditions within the paleochannel. No Mn-reducing microorganisms were detected. These findings indicate that Fe and Mn cycling not only reflects redox zonation but also actively contributes to the establishment of microbial niches where chloroethene degradation can occur.

### Spatial distribution of genera of microorganisms capable of sulfide oxidation, sulfate reduction, methanogenesis, and fermentation

As with Fe and Mn oxidizers, sulfide-oxidizing organisms were more abundant in the interior zones of the aquifer than in the HZ (profile X-X′). In the cases of sulfate-reducing and methanogenic microorganisms, there was a greater predominance of methanogens in the HZ than sulfate reducers. This is in accordance with the dilution effect observed in the HZ (section “Groundwater redox zonation of the aquifer-hyporheic zone system”). This effect resulted in a lower concentration of sulfate in groundwater, making it less bioavailable and hindering its degradation (Puigserver et al. 2014), even under sulfate-reducing redox conditions (Fig. 4). This reduced sulfate bioavailability is also consistent with a shift in microbial niches within these communities and a greater development of methanogenic niches. Towards the interior of the aquifer, the increase in the proportion of the sulfate contribution by the fractured marlstone substrate (coming from the oxidation of pyrite in these marlstones) promoted the development of sulfate-reducing microorganisms, which is accompanied by greater isotopic fractionation of sulfate (Puigserver et al. 2014); this is evidence that sulfate-reduction processes were occurring.

The predominance of methanogenic conditions in the HZ (profile X-X′) was accompanied by a greater presence of fermenting microorganisms, ensuring the presence of bioavailable electrons in the medium due to the high proportion of OM present (Xu et al. 2019; see Fig. 2B and the “Geological and hydrogeological control of the aquifer-hyporheic zone system on the distribution of contaminants and their biodegrading microorganisms” section).

The geological structure also controls the spatial distribution of methanogenic, sulfate-reducing, fermentative, and sulfide-oxidizing microorganisms, with methanogenic and sulfate-reducing communities predominating in interchannel areas (transect D-D′), whereas fermenters and sulfide oxidizers are more abundant in paleochannel areas (transects C–C′, E-E′, and F-F′). An exception to this pattern is observed in profile B-B′, where a higher input of organic matter (see the “Spatial distribution of the genera of microorganisms capable of oxidize organic matter, nitrifying, and denitrifying” subsection) promotes an increase in sulfate-reducing and methanogenic microorganisms towards the interior of the aquifer (Ghezzi et al. 2021; Peiffer et al. 2021). This redox sequence, characterized by the progression from Fe and Mn reduction to sulfate reduction and methanogenesis, governs the availability of electron acceptors and donors, ultimately controlling the efficiency of reductive dechlorination.

Regarding sulfide-oxidizing genera in surface waters, the following were identified (see Figure SI–6 and Figure SI–7 in the SI): *Tumebacillus*, a sulfur bacterium that oxidizes sulfur and utilizes various carbon sources to support growth (Duan et al. 2020); and *Limnohabitans*, chemoorganotrophic and facultatively anaerobic bacteria that grow better under aerobic conditions (Hahn et al. 2010; Kasalický et al. 2010) and are able to oxidize ammonia and sulfur, indicating great metabolic versatility (Zeng et al. 2012). *Thiocapsa* was also identified in surface water. Some species of this genus, such as *Thiocapsa* spp., are anoxygenic phototrophs that use hydrogen sulfide, thiosulfate, elemental sulfur, and molecular hydrogen as electron donors during photolithotrophic growth (Wu et al. 2021; Caumette et al. 2004). These microorganisms were not identified either in the HZ (profile X-X′) or in the interchannel areas, while in the internal zones of the paleochannels (transect C–C′), *Limnohabitans* was mainly detected, with *Thiocapsa* being present to a lesser extent. This finding is consistent with the predominance of denitrification and Mn-reduction conditions, coupled with the presence of OM, as observed, for example, by piezometer SD55.

The sulfate-reducing microorganisms (Figure SI–6 and Figure SI–7 in the SI) identified in surface water included *Bdellovibrio*, a genus of obligate predatory bacteria that selectively feed on a wide range of Gram-negative bacteria, specifically associated with the major bacterial group of sulfate reduction (Paix et al. 2019; Hespell et al. 1984); *Peredibacter*, a genus of predatory, Gram-negative, bacteriovorous species that require a Gram-negative host (Davidov and Jurkevitch 2004); and *Halobacteriovorax*, which preys on Gram-negative bacteria (Young et al. 2018). In the HZ, these microorganisms were not detected, as redox conditions were methanogenic, displacing sulfate reducers (additionally, low sulfate concentrations due to dilution hinder the development of these sulfate-reducing microorganisms; Jørgensen et al. 2019).

In the interchannel areas (Fig. 5 A, B), *Bdellovibrio* and *Thermofilum* (an archaebacterium) were identified. *Thermofilum* is a genus of thermophilic, anaerobic sulfur-respiring microorganisms (Joseph et al. 2021). Specifically, *Thermofilum* was identified in the zone where free-phase DNAPL PCE had been previously detected (currently in the residual phase) and where sulfate-reducing conditions were observed in the present study. This highlights that these microorganisms are capable of withstanding extreme conditions, such as the high toxicity of free- or residual-phase DNAPL PCE, similar to what was observed by Puigserver et al. (2023).

With regard to sulfate-reducing microorganisms, these were well developed in interchannel areas (*Bdellovibrio*, *Peredibacter*, *Thermofilum*, *Halobacteriovorax*, *Bacteriovorax*, *Vampirovibrio*, and *Peredibacter*) and to a lesser extent in the paleochannel areas of the inner aquifer zone (Figure SI–6 and Figure SI–7 in the SI).

The methanogenic microorganisms (Figure SI–6 and Figure SI–7 in the SI) identified in surface waters are as follows: *Methanomassiliicococcus*, described as archaea capable of degrading different hydrocarbons, as well as providing H_2_ to the medium (Chen et al. 2023), and *Methanospirillum*, a genus of hydrogenotrophic methanogens that use H_2_/CO_*2*_ as a primary substrate to produce methane. Some species of this genus also use formate or secondary alcohols and CO_2_ (Munoz-Palazon et al. 2022). Additionally, *Methanospirillum* sp. could be microbial indicators for removing 1,1-DCE and VC in degradation (Chen and Chang 2020). However, well-developed *Methanomassiliicococcus* and *Methanospirillum* were found mainly in the HZ and in interchannel areas where *Pseudorhodobacter* was also identified, which had previously been associated with methanotrophic genera such as *Methylomonas* or *Methylobacter*, known to thrive in methane-generating environments (Němeček et al. 2018). However, species from these two genera, such as *Pseudomonas denitrificans* or *Brevundimonas denitrificans*, have also been associated with environments where nitrate reduction occurs, using nitrate as an electron acceptor (Toumi et al. 2021).

The microbial diversity of fermentative microorganisms was significant (Figure SI–6 and Figure SI–7 in the SI), with these organisms being present in both surface water and groundwater. Among them, the genus *Opitutus* (Chin et al. 2001) stands out due to its widespread occurrence across most samples and its highest abundance in interchannel areas, where it contributes to maintaining electron availability for reduction reactions. Overall, the predominance of fermentative and methanogenic microorganisms in the HZ ensures a continuous supply of electron donors (e.g., H₂), which are essential for sustaining reductive dechlorination processes.

### Genera of oxidative and reductive dechlorinating microorganisms

The distribution of oxidative and reductive dechlorinating microorganisms represents a direct indicator of the biodegradation potential of chloroethenes and reflects the combined influence of geological, hydrogeological, and geochemical controls. In this context, the coincidence between isotopic fractionation patterns and the abundance of reductive dechlorinating microorganisms confirms that biodegradation is primarily driven by microbial processes under reducing conditions.

**Fig. 7:**
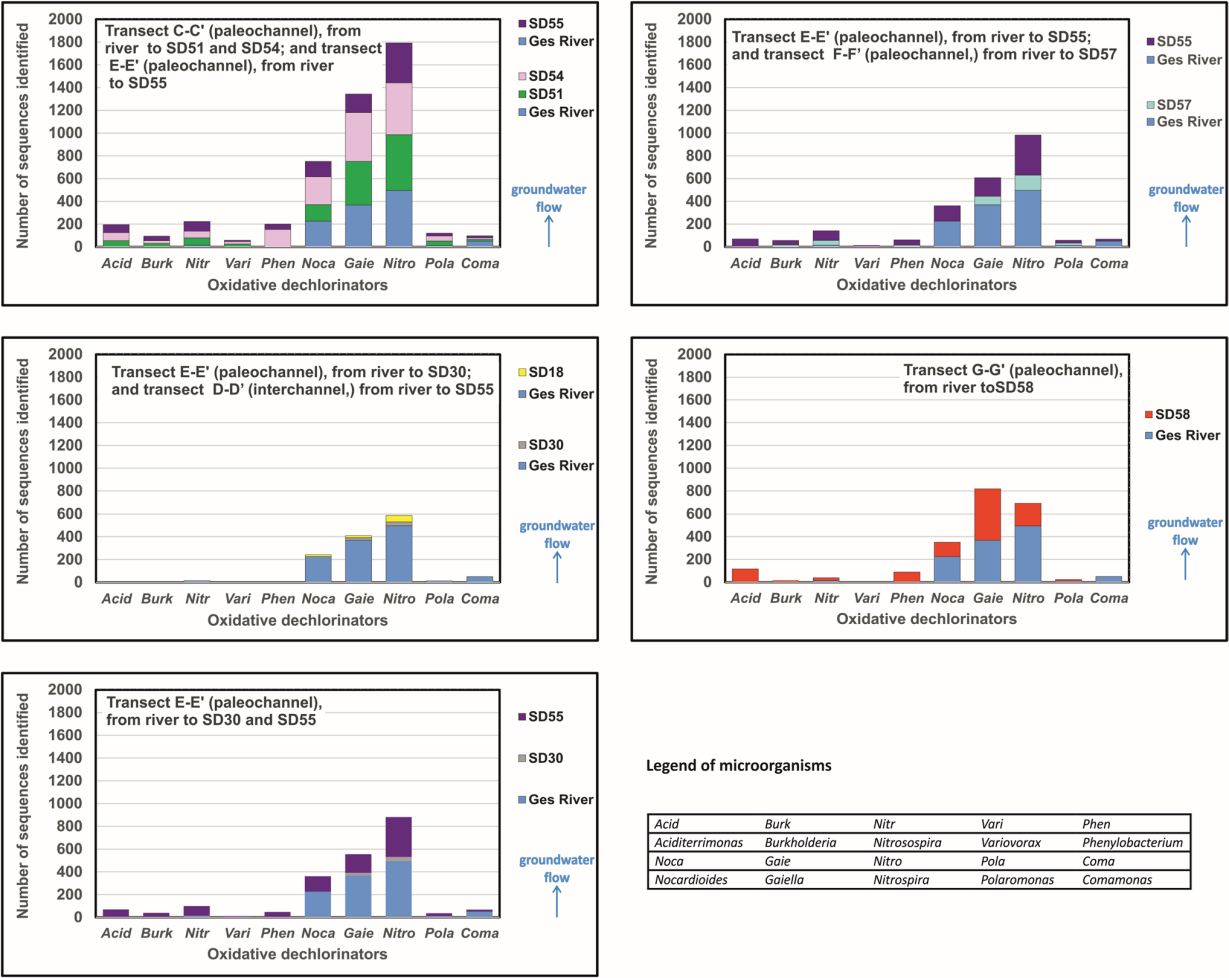
Microorganisms capable of oxidative dechlorination. Each color within the bars represents a piezometer within the monitoring network of the ACA. Spring 2022 sampling survey

The distribution of dechlorinating microorganisms in the HZ differed somewhat from that observed for the other microorganisms analyzed in the “Spatial distribution of the genera of microorganisms capable of oxidize organic matter, nitrifying, and denitrifying”; “Spatial distribution of the genera of Mn-oxidizing, Fe-oxidizing, and Fe-reducing microorganisms”; and “Spatial distribution of genera of microorganisms capable of sulfide oxidation, sulfate reduction, methanogenesis, and fermentation” subsections, which are involved in various biogeochemical reactions and redox zones at the study site. In Fig. 5B, a higher proportion of oxidative dechlorinators is evident in the HZ compared to the interior of the aquifer, particularly in paleochannel areas (transects F-F′ and G-G′ in Fig. 7) but also in paleochannel B-B′, while reductive dechlorinators were more significant in interchannel areas. This distribution is primarily controlled by the spatial occurrence of DNAPL PCE, its dissolution dynamics, and the associated redox conditions, which together determine contaminant bioavailability and the dominant biodegradation pathways.

This difference can be explained by the distribution of DNAPLs in the medium. In the HZ (profile X-X′), DNAPL PCE pools were placed in the past and have since evolved into a mature state (aged pools; see the “Chloroethene distribution and ^13^C isotopic fractionation along the aquifer-hyporheic zone system” section), where DNAPL PCE exists in a residual phase. The elevated concentration of dissolved PCE resulting from continuous flushing by groundwater flow from the aged pools can be toxic to non-dechlorinating microorganisms in the environment (Puigserver et al. 2016). The prevalence of oxidative dechlorinators in the HZ (profile X-X′) is linked to their greater presence in surface waters, which, being influent in the river in the right bank, penetrate the aquifer mainly through paleochannel areas, thus favoring the presence of oxidative dechlorinators in these areas. Additionally, an increase in the proportion of reductive dechlorinators towards the interior of the aquifer was observed within the paleochannels (Fig. 5B).

It is important to emphasize the greater presence of oxidative dechlorinators in paleochannel areas compared to interchannel areas, as this ensures that the cDCE metabolites (also VC; see Fig. 5 A), which were highly present in the source zones (formed by the aged pools in the residual phase), can be degraded oxidatively to CO_2_ (Lohner and Tiehm 2009; Zeppilli et al. 2021). This clarifies that although VC was detected in the source where reducing redox conditions dominated, its presence alone does not account for the significant natural attenuation observed in the medium since the contamination sources were placed. Rather, what transpired was the coupling of reductive dechlorinating microorganisms in the interchannel areas with oxidative microorganisms in the paleochannels similar to what was observed by Lohner and Tiehm 2009 in aerobic column experiments. This coupling favored the total mineralization of DNAPL PCE, which demonstrates the validity of the working hypothesis.

The most abundant dechlorinating microorganisms identified were the oxidative ones. The following were identified in surface waters: *Nitrosospira*, capable of degrading chloroethenes (Aggarwal et al. 2018); *Nocardioides*, aerobic and related to the aerobic degradation of contaminants such as VC (Liu et al. 2018; Schmidt et al. 2014); *Gaiella*, identified together with *Nocardioides* in areas where oxidative dechlorination occurs (Luo et al. 2021); *Nitrospira*, also described as capable of dechlorination (Yan et al. 2021); *Polaromonas*, which is aerobic and chemoorganotrophic (Irgens et al. 1996) and able to use cDCE as the sole source of carbon and aerobically dechlorinate it to CO_2_ (Giddings et al. 2010; Jennings et al. 2009; Mattes et al. 2008); and *Comamonas*, capable of co-metabolizing DCE (Zalesak et al. 2017).

**Fig. 8:**
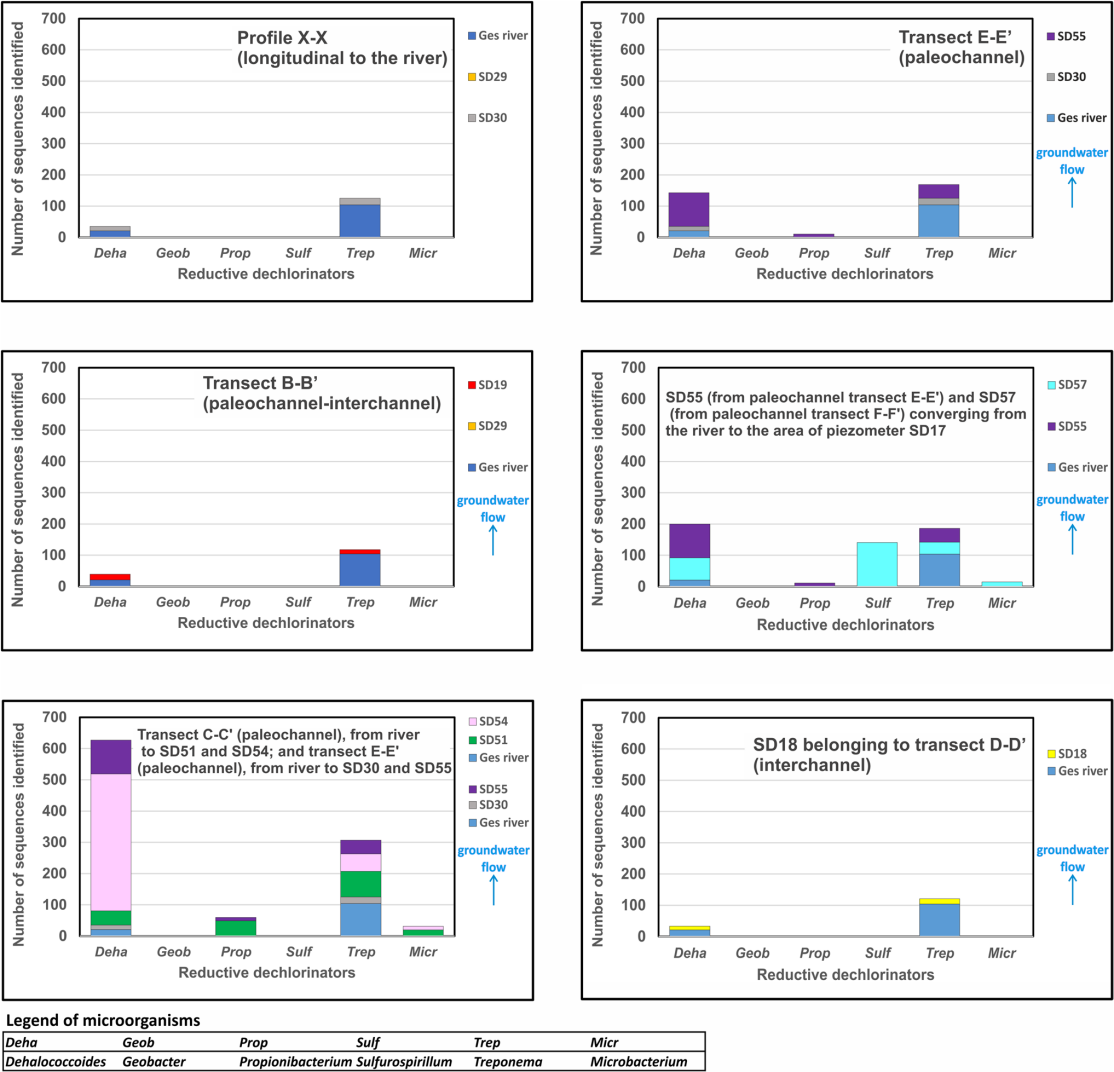
Microorganisms capable of reductive dechlorination. Each color within the bars represents a piezometer within the monitoring network of the ACA. Spring 2022 sampling survey

On the contrary, the predominance of anaerobic conditions in the HZ (Figs. 4 and 2, profile X-X′) led to the identification of only the genera *Gaiella* and *Nitrospira* in this area (Fig. 8), while in the paleochannel area corresponding to transect C–C′ (located further inside the aquifer), the same genera of microorganisms found in surface waters were found in addition to *Aciditerrimonas* (also associated with reductive dechlorination; Puigserver et al. 2023); *Burkholderia* (also detected in areas where dechlorination occurs; Qiu et al. 2023); *Variovorax*, identified as being capable of degrading phenol and other aromatic compounds (Futamata et al. 2005; Vallaeys et al. 1998) and associated with *Nocardioides*, *Sediminibacterium*, *Aquabacterium*, and *Pseudomonas* in the aerobic degradation of VC (Wilson et al. 2016); and *Phenylobacterium*, which has been detected in the dechlorination of polychlorinated biphenyls (PCBs) and is associated with *Dehalococcoides* sp., *Ochrobactrum* sp., *Parasegetibacter* sp., and *Thermithiobacillus* sp. (Jing et al. 2018).

The progressive increase of *Gaiella*, *Nitrospira*, *Nocardioides*, *Phenylobacterium*, and to a lesser extent *Aciditerrimonas* and *Nitrosospira* is noteworthy, coinciding with the paleochannel area C–C′, with a significant decrease in chloroethenes, including both PCE and its metabolites (Fig. 7), and redox conditions shifting from iron reduction to sulfate reduction.

In other paleochannel areas near the river, such as those where transects F-F′ and G-G′, which correspond to the larger paleochannels, are located, the greater contribution of surface water and their closer proximity to the river explain the identification of the genera *Gaiella* and *Nitrospira*. Additionally, other less abundant microorganisms similar to those identified in transect C–C′ were identified along the groundwater flow, including *Aciditerrimonas*, *Burkholderia*, *Phenylobacterium*, *Polaromonas*, and *Nitrosospira* (Fig. 7). The greater dilution effect (described in the “Groundwater redox zonation of the aquifer-hyporheic zone system” section) and the lower proportion of reductive dechlorination metabolites explain how the higher natural attenuation produced by the oxidative dechlorination of cDCE and VC generated in the HZ occurred more easily along the groundwater flow.

The predominance of oxidative dechlorinators in paleochannel areas favored the oxidation of cDCE and VC, which are lighter and more difficult to degrade metabolites under reducing conditions according to Lohnerand and Stiehm (2009). This explains why these metabolites were practically not identified in paleochannel areas (Fig. 5 A).

In contrast to paleochannel areas, cDCE was identified in interchannel areas, such as transect D-D′, where monitoring piezometer SD18 is located (Figs. 5 and 6). At this piezometer, an aged pool of DNAPL PCE was detected in the past; it was already in the residual phase by Puigserver et al. (2014) and continues to be in the residual phase today (see the “Chloroethene distribution and ^13^C isotopic fractionation along the aquifer-hyporheic zone system” section). The more reducing conditions of the environment hindered the presence of oxidative dechlorinators. Thus, although the area of monitoring piezometer SD18 is within the aquifer at a distance from the river similar to that of the monitoring piezometers in the paleochannel area of transect C–C′, there was no increase in the diversity of oxidative dechlorinators, and only *Gaiella*, *Nitrospira*, and *Nocardioides* were identified. Additionally, an increase in the microbial load of these microorganisms was detected along the flow, possibly due to greater adaptation to the environment.

In the case of reductive dechlorinators, these were scarce in surface water, the HZ, and the interchannel areas, although the genera *Dehalococcoides* and *Treponema* were detected. The *Dehalococcoides* genus is widely described as reductive dechlorinators of chlorinated solvents (Dutta et al. 2022), and some species of this genus are able to completely or incompletely biodegrade chlorinated solvents. *Treponema*, although it has been found in studies of contamination by chlorinated solvents in groundwater, is not known to play a role in reductive dechlorination (Hellal et al. 2021; Lee et al. 2015; Kotik et al. 2013).

On the contrary, a higher proportion of reductive dechlorinators were detected in paleochannel areas (Fig. 5B), including microorganisms from the genera *Geobacter* and *Dehalococcoides* (whose species *Dehalococoides mccartyi* is capable of reductively degrading PCE to ethene; Chang et al. 2011); *Clostridium* and *Desulfovibrio*, which are facultative bacteria with an adaptive metabolism that leads to reductive dechlorination (Atashgahi et al. 2016) and have also been described as providers of the H_2_, acetate, and cobamides required by *Dehalococcides*, preventing the inactivation of exogenous vitamins (Peng et al. 2020); *Propionibacterium*, identified as capable of reductively degrading PCE to ethene and also described as a PCE biodegrader; *Sulfurospirillum*, which are also facultative bacteria with an adaptive metabolism for reductive dechlorination (Atashgahi et al. 2016) that are capable of biodegrading PCE (Jin et al. 2023); and *Treponema* and *Microbacterium* (identified in the sequential degradation of chloroethenes; Bertolini et al. 2023).

Among the most abundant genera of identified microorganisms were *Dehalococcoides* and *Treponema*, although significant increases in the microbial load of *Propionibacterium* (Fig. 8) were also detected in some sectors in the interior of the aquifer, explaining the complete PCE degradation.

The occurrence of reductive dechlorinators in zones associated with Fe-reducing and methanogenic conditions highlights the importance of Fe cycling in sustaining electron flow and supporting organohalide respiration. For these reason, Fe and Mn are not passive indicators but active geochemical drivers that regulate electron flow, redox zonation, and microbial niche differentiation, ultimately controlling the efficiency of chloroethene biodegradation.

This coupling between redox conditions, microbial communities, and contaminant distribution highlights the importance of site-specific characteristics in controlling natural attenuation processes. In this context, the coexistence of reductive dechlorination in interchannel areas and oxidative degradation in paleochannels demonstrates a coupled biodegradation system, where geological heterogeneity and groundwater flow generate complementary redox niches that enable the complete mineralization of chloroethenes. Overall, this behavior reflects a feedback mechanism in which hydrogeological conditions regulate contaminant and electron acceptor availability, while microbial activity simultaneously drives the redox evolution of the system.

## Conclusions

The most relevant and novel conclusions of the study are presented below. These conclusions provide new insights into microbial dynamics and the degradation processes of chlorinated solvents in aquifers, which can inform environmental management strategies and remediation approaches in contaminated environments.

### The coupling of oxidative and reductive dechlorination enhances natural attenuation

The synergy between oxidative and reductive microorganisms in different zones of the aquifer promotes the total mineralization of chloroethenes. This interaction is crucial for the complete degradation of compounds like cDCE and VC, showcasing an effective natural attenuation within the environment.

### There are differences in the distribution of dechlorinating microorganisms according to redox conditions

A distinct distribution of oxidative and reductive microorganisms occurs depending on the redox conditions, with oxidative microorganisms predominating in areas influenced by surface water and a higher proportion of reductive microorganisms in paleochannel and interchannel areas under reducing conditions. This variability underscores the importance of understanding environmental conditions for predicting dechlorinating microbial activity.

### Paleochannel areas are significant in the complete degradation of chloroethenes

Paleochannel areas exhibit greater diversity and activity of oxidative and reductive microorganisms and enhanced mixing with surface water, which promotes dilution, DNAPL dissolution, and the oxidative degradation of metabolites such as cDCE and VC. In contrast, interchannel areas favor reductive dechlorination due to higher organic matter content and more reducing conditions. This spatial complementarity enables complete degradation across the system.

### Microbial adaptation to groundwater flow occurs

Significant microbial adaptation occurs along the groundwater flow, with changes in microbial diversity and abundance being observed along the flow path. This reflects the combined influence of hydrogeological transport, redox evolution, and contaminant distribution on microbial community structure.

### Bioremediation strategies could be based on specific microorganisms

The identification of key microorganisms involved in chloroethene degradation, such as the genera *Dehalococcoides* and *Treponema*, as well as aerobic degraders of cDCE and VC (e.g., *Nocardioides* and *Polaromonas*), suggests the possibility of designing targeted bioremediation strategies harnessing these microorganisms to enhance in situ chloroethene degradation efficiency.

Dilution and proximity to the river affect the efficiency of natural attenuation. Greater contaminant dilution near the river correlates with an increased efficiency of natural attenuation, especially in paleochannel areas adjacent to the inflow of surface water. In addition to dilution, surface water input modifies redox conditions and electron acceptor availability, thereby reducing competition between microbial pathways and enhancing dechlorination processes.

### Dilution and proximity to the river affect the efficiency of natural attenuation

Greater contaminant dilution near the river correlates with an increased efficiency of natural attenuation, especially in paleochannel areas adjacent to the inflow of surface water. In addition to dilution, surface water input modifies redox conditions and electron acceptor availability, thereby reducing competition between microbial pathways and enhancing dechlorination processes.

The integration of concentration data with compound-specific isotope analysis (CSIA) and microbial community characterization demonstrates that natural attenuation cannot be inferred solely from concentration decreases, as dilution processes may mask degradation. Instead, robust assessment requires multiple lines of evidence, including isotopic enrichment, metabolite formation, and the presence of functional microbial groups.

The results obtained in this study allow the establishment of a transferable conceptual framework to evaluate the natural attenuation potential of chloroethenes in other hydrographic basins, particularly within hyporheic zones. In this context, we consider that assessing natural attenuation potential requires identifying the key controls governing the processes that promote it, focusing on three fundamental components: (i) characterization of the physical environment; (ii) definition of the prevailing geochemical conditions; and (iii) identification of the dominant microbial communities.

 To facilitate extrapolation to other sites, a minimal assessment workflow is proposed:

(i) **Hydrogeological screening**, including the identification of sedimentary heterogeneity (e.g., preferential flow paths such as paleochannels versus low-permeability zones), hydraulic conductivity contrasts, and groundwater flow patterns that control residence times and contaminant transport.
(ii) **Geochemical and redox characterization**, focusing on the identification of well-developed redox gradients (from oxic to methanogenic conditions) and the distribution of key electron acceptors (O₂, NO₃⁻, Fe(III), SO₄^2^⁻), which determine the potential for both oxidative and reductive degradation pathways.
(iii) **Verification of in situ degradation**, based on multiple lines of evidence, including concentration trends, the presence of daughter products (TCE, cDCE, VC), and isotopic fractionation patterns indicative of biodegradation.
(iv) **Microbial functional assessment**, including the identification of key degraders (e.g., *Dehalococcoides* for reductive dechlorination and aerobic degraders of cDCE and VC), as well as supporting microbial communities involved in electron donor and acceptor cycling.
(v) **Conceptual model integration**, combining the previous steps to determine whether the system supports coupled oxidative–reductive processes required for complete mineralization.

First, it is essential to characterize sedimentary heterogeneity, particularly from a textural perspective, in order to determine where sorption processes prevail over contaminant mobilization. This characterization should be complemented by the evaluation of hydraulic conductivity across different geological structures, such as paleochannels and interchannel zones, to assess hydrogeological connectivity. The presence of paleochannels and active hyporheic zones plays a key role in controlling surface water–groundwater interactions, DNAPL dissolution, and the development of redox niches.

Second, the identification of well-developed redox gradients (ranging from oxic to methanogenic conditions) is critical, as it allows the inference of the coexistence of oxidative and reductive dechlorination processes, which is a prerequisite for the complete mineralization of PCE and its daughter products. These gradients are closely linked to the distribution of organic matter and should therefore be carefully evaluated.

The predominance of reductive versus oxidative microbial communities ultimately determines whether toxic intermediates accumulate or are further degraded.

Third, the availability and spatial distribution of organic matter control the system’s capacity to sustain reductive processes, acting as an electron donor source and regulating oxygen consumption. These factors should be complemented with direct lines of evidence of degradation, including concentration trends, the presence of degradation products, and isotopic fractionation, as well as the identification of functional microbial communities.

Overall, a system can be considered “equipped” for natural attenuation when it exhibits: (i) active hydraulic connectivity enabling interaction between different hydrogeological compartments; (ii) well-defined and spatially variable redox zonation; (iii) sufficient organic matter availability to sustain reducing conditions; and (iv) coexisting microbial communities capable of both reductive and oxidative degradation.

Conversely, when one or more of these conditions are not met—such as limited electron donor availability, absence of key microbial populations, or lack of redox gradients—natural attenuation may be incomplete, leading to the accumulation of toxic intermediates (e.g., cDCE or VC).

In such cases, the system may be improved through targeted interventions, including the addition of electron donors, bioaugmentation with specific dechlorinating microorganisms, or the modification of hydrodynamic conditions (e.g., enhancing river–aquifer interactions) to promote the development of favorable redox niches.

Although this study is based on a specific case, the proposed framework is transferable to other hydrographic basins, particularly those characterized by alluvial aquifers with active hyporheic exchange. However, its applicability depends on site-specific factors such as geological complexity, scale, contaminant loading, and external inputs (e.g., agricultural or wastewater sources), which must be considered when extrapolating the results to different environmental settings.

## Supplementary Information

Below is the link to the electronic supplementary material.

ESM1 (DOCX 2.55 MB)

## Data Availability

The datasets generated and analyzed during the current study are available in the Mendeley repository, 10.17632/4vcpd832ct.1.

## References

[CR1] Aggarwal S, Gomez-Smith CK, Jeon Y, LaPara TM, Waak MB, Hozalski RM (2018) Effects of chloramine and coupon material on biofilm abundance and community composition in bench-scale simulated water distribution systems and comparison with full-scale water mains. Environ Sci Technol 52(22):13077–13088. 10.1021/acs.est.8b02607 30351033 10.1021/acs.est.8b02607

[CR2] Aguilar-Rangel EJ, Prado BL, Vásquez-Murrieta MS, Estrada-de Los Santos P, Siebe C, Falcón LI, Santillán J, Alcántara-Hernández RJ (2020) Temporal analysis of the microbial communities in a nitrate-contaminated aquifer and the co-occurrence of anammox, n-damo and nitrous-oxide reducing bacteria. J Contam Hydrol 234:103657. 10.1016/j.jconhyd.2020.103657 32777591 10.1016/j.jconhyd.2020.103657

[CR3] Albuquerque L, França L, Rainey FA, Schumann P, Nobre MF, da Costa MS (2011) *Gaiella occulta* gen. nov., sp. nov., a novel representative of a deep branching phylogenetic lineage within the class Actinobacteria and proposal of Gaiellaceae fam. nov. and Gaiellales ord. nov. Syst Appl Microbiol 34(8):595–599. 10.1016/j.syapm.2011.07.001 10.1016/j.syapm.2011.07.001 21899973

[CR4] Amaral HI, Aeppli C, Kipfer R, Berg M (2011) Assessing the transformation of chlorinated ethenes in aquifers with limited potential for natural attenuation: added values of compound-specific carbon isotope analysis and groundwater dating. Chemosphere 85(5):774–781. 10.1016/j.chemosphere.2011.06.063 21741066 10.1016/j.chemosphere.2011.06.063

[CR5] Anderson JE, Mccarty PL (1997) Transformation yields of chlorinated ethenes by a methanotrophic mixed culture expressing particulate methane monooxygenase. Appl Environ Microbiol 63:687–693. 10.1128/aem.63.2.687-693.1997 9023946 10.1128/aem.63.2.687-693.1997 PMC168358

[CR6] Antoniou K, Mamais D, Pantazidou M (2019) Reductive dechlorination of trichloroethene under different sulfate-reducing and electron donor conditions. J Contam Hydrol 226:103519, 103519. 10.1016/j.jconhyd.2019.103519 31302292 10.1016/j.jconhyd.2019.103519

[CR7] Atashgahi S, Lu Y, & Smidt H (2016) Overview of known organohalide-respiring bacteria—phylogenetic diversity and environmental distribution. Organohalide-respiring bacteria 63–105. 10.1007/978-3-662-49875-0_5

[CR8] Aulenta F, Canosa A, Leccese M, Petrangeli Papini M, Majone M, Viotti P (2007) Field study of in situ anaerobic bioremediation of a chlorinated solvent source zone. Ind Eng Chem Res 46(21):6812–6819. 10.1021/ie070048m

[CR9] Azizian M, Boano F, Cook PL, Detwiler RL, Rippy MA, Grant SB (2017) Ambient groundwater flow diminishes nitrate processing in the hyporheic zone of streams. Water Resour Res 53(5):3941–3967. 10.1002/2016WR020048

[CR10] Bae S, Lee W (2012) Enhanced reductive degradation of carbon tetrachloride by biogenic vivianite and Fe (II). Geochim Cosmochim Acta 85:170–186. 10.1016/j.gca.2012.02.023

[CR11] Bellizia E, Boaga J, Fontana A, D’Alpaos A, Cassiani G, Ghinassi M (2021) Impact of genesis and abandonment processes of a fluvial meander on geometry and grain-size distribution of the associated point bar (Venetian Plain, Italy). Mar Pet Geol 127:104951. 10.1016/j.marpetgeo.2021.104951

[CR12] Berns EC, Sanford RA, Valocchi AJ, Strathmann TJ, Schaefer CE, Werth CJ (2019) Contributions of biotic and abiotic pathways to anaerobic trichloroethene transformation in low permeability source zones. J Contam Hydrol 224:103480. 10.1016/j.jconhyd.2019.04.003 31006532 10.1016/j.jconhyd.2019.04.003

[CR13] Bertolini M, Zecchin S, Cavalca L (2023) Sequential anaerobic/aerobic microbial transformation of chlorinated ethenes: use of sustainable approaches for aquifer decontamination. Water (Basel) 15(7):1406. 10.3390/w15071406

[CR14] Boano F, Harvey JW, Marion A, Packman AI, Revelli R, Ridolfi L, Wörman A (2014) Hyporheic flow and transport processes: mechanisms, models, and biogeochemical implications. Rev Geophys 52(4):603–679. 10.1002/2012RG000417

[CR15] Bonsor HC, MacDonald AM, Ahmed KM, Burgess WG, Basharat M, Calow RC, Dixit A, Foster SSD, Gopal K, Lapworth DJ, Moench M, Mukherjee A, Rao MS, Shamsudduha M, Smith L, Taylor RG, Tucker J, van Steenbergen F, Yadav SK, Zahid A (2017) Hydrogeological typologies of the Indo-Gangetic basin alluvial aquifer. South Asia. Hydrogeol J 25(5):1377. 10.1007/s10040-017-1550-z 32025191 10.1007/s10040-017-1550-z PMC6979522

[CR16] Boulton AJ, Findlay S, Marmonier P, Stanley EH, Valett HM (1998) The functional significance of the hyporheic zone in streams and rivers. Annu Rev Ecol Syst 29(1):59–81. 10.1146/annurev.ecolsys.29.1.59

[CR17] Bouwer EJ et al (1994) Bioremediation of chlorinated solvents using alternate electron acceptors. In: Norris RD (ed) Handbook of bioremediation. Lewis Publishers, pp 149–175

[CR18] Bradley PM (2003) History and ecology of chloroethene biodegradation: a review. Bioremediat J 7(2):81–109. 10.1080/713607980

[CR19] Bradley PM, Chapelle FH (2010) Biodegradation of chlorinated ethenes. In: Stroo, H., Ward, C. (eds) In situ remediation of chlorinated solvent plumes. SERDP/ESTCP Environmental Remediation Technology. Springer, New York, NY, pp 39–67. 10.1007/978-1-4419-1401-9_3

[CR20] Bradley PM, Chapelle FH (2011) Microbial mineralization of dichloroethene and vinyl chloride under hypoxic conditions. Groundwater Monit Remediation 31(4):39–49. 10.1111/j.1745-6592.2011.01339.x

[CR21] Bradley PM, Chapelle FH (2000) Aerobic microbial mineralization of dichloroethene as sole carbon substrate. Environ Sci Technol 34:221–223. 10.1021/es990785c

[CR22] Broholm K, Ludvigsen L, Jensen TF, Østergaard H (2005) Aerobic biodegradation of vinyl chloride and cis-1,2-dichloroethylene in aquifer sediments. Chemosphere 60(11):1555–1564. 10.1016/j.chemosphere.2005.02.056 16083761 10.1016/j.chemosphere.2005.02.056

[CR23] Burston MW, Nazari MM, Bishop PK, Lerner DN (1993) Pollution of groundwater in the Coventry region (UK) by chlorinated hydrocarbon solvents. J Hydrol 149(1–4):137–161. 10.1016/0022-1694(93)90104-H

[CR24] Buss SR, Herbert AW, Morgan P, Thornton SF, Smith JWN (2004) A review of ammonium attenuation in soil and groundwater. Q J Eng Geol Hydrogeol 37(4):347–359. 10.1144/1470-9236/04-005

[CR25] Butler EC, Chen L, Darlington R (2013) Transformation of trichloroethylene to predominantly non-regulated products under stimulated sulfate reducing conditions. Groundwater Monit Remediation 33(3):52–60. 10.1111/gwmr.12015

[CR26] Cárdenas MB (2015) Hyporheic zone hydrologic science: a historical account of its emergence and a prospectus. Water Resour Res 51(5):3601–3616. 10.1002/2015WR017028

[CR27] Caumette P, Guyoneaud R, Imhoff JF, Süling J, Gorlenko V (2004) *Thiocapsa marina* sp. nov., a novel, okenone-containing, purple sulfur bacterium isolated from brackish coastal and marine environments. Int J Syst Evol Microbiol 54:1031–1036. 10.1099/ijs.0.02964-0 15280266 10.1099/ijs.0.02964-0

[CR28] Chang YC, Ikeutsu K, Toyama T, Choi D, Kikuchi S (2011) Isolation and characterization of tetrachloroethylene-and cis-1, 2-dichloroethylene-dechlorinating propionibacteria. J Ind Microbiol Biotechnol 38(10):1667. 10.1007/s10295-011-0956-1 21437617 10.1007/s10295-011-0956-1

[CR29] Chapelle FH (1996) Identifying redox conditions that favor the natural attenuation of chlorinated ethenes in contaminated ground-water systems. Symposium on natural attenuation of chlorinated organics in ground water. US Environmental Protection Agency, Washington, D.C., pp 17–20

[CR30] Chapman SW, & Parker BL (2005) Plume persistence due to aquitard back diffusion following dense nonaqueous phase liquid source removal or isolation. Water Resour Res 41(12). 10.1029/2005WR004224

[CR31] Chartrand MM, Morrill PL, Lacrampe-Couloume G, Sherwood Lollar B (2005) Stable isotope evidence for biodegradation of chlorinated ethenes at a fractured bedrock site. Environ Sci Technol 39(13):4848–4856. 10.1021/es048592z 16053083 10.1021/es048592z

[CR32] Chee-Sanford J, Tian D, Sanford R (2019) Consumption of N2O and other N-cycle intermediates by *Gemmatimonas aurantiaca* strain T-27. Microbiol 165(12):1345–1354. 10.1099/mic.0.000847 10.1099/mic.0.000847 31580255

[CR33] Chen C, Li L, Wang Y, Dong X, & Zhao FJ (2023) Methylotrophic methanogens and bacteria synergistically demethylate dimethylarsenate in paddy soil and alleviate rice straighthead disease. The ISME J 1–11. 10.1038/s41396-023-01498-7 10.1038/s41396-023-01498-7 PMC10579292 37604918

[CR34] Chen TW, Chang SC (2020) Potential microbial indicators for better bioremediation of an aquifer contaminated with vinyl chloride or 1,1-dichloroethene. Water Air Soil Pollut 231:1–23. 10.1007/s11270-020-04538-6

[CR35] Chen HM, Wu MT (2017) Residential exposure to chlorinated hydrocarbons from groundwater contamination and the impairment of renal function-an ecological study. Sci Rep 7(1):40283. 10.1038/srep40283 28067285 10.1038/srep40283 PMC5220340

[CR36] Chi Z, Liu X, Li H, Liang S, Luo Y-H, Zhou C, Rittmann BE (2023) Co-metabolic biodegradation of chlorinated ethene in an oxygen- and ethane-based membrane biofilm reactor. Sci Total Environ 905:167323. 10.1016/j.scitotenv.2023.167323 37742949 10.1016/j.scitotenv.2023.167323

[CR37] Chin K-J, Liesack W, Janssen PH (2001) *Opitutus terrae* gen. nov., sp. nov., to accommodate novel strains of the division ‘Verrucomicrobia’ isolated from rice paddy soil. Int J Syst Evol Microbiol 51:1965–1968. 10.1099/00207713-51-6-1965 11760935 10.1099/00207713-51-6-1965

[CR38] Christof O, Seifert R, Michaelis W (2002) Volatile halogenated organic compounds in European estuaries. Biogeochem 59:143–160. 10.1023/A:1015592115435

[CR39] Cole JR, Wang Q, Fish JA, Chai B, McGarrell DM, Sun Y, Brown CT, Porras-Alfaro A, Kuske CR, Tiedje JM (2014) Ribosomal database project: data and tools for high throughput rRNA analysis. Nucleic Acids Res. 10.1093/nar/gkt1244 10.1093/nar/gkt1244 PMC3965039 24288368

[CR40] Coleman NV, Mattes TE, Gossett JM, Spain JC (2002) Biodegradation of cis- dichloroethene as the sole carbon source by a β-Proteobacterium. Appl Environ Microbiol 68(6):2726–2730. 10.1128/AEM.68.6.2726-2730.2002 12039726 10.1128/AEM.68.6.2726-2730.2002 PMC123969

[CR41] Coleman NV, Spain JC (2003) Epoxyalkane: coenzyme M transferase in the ethene and vinyl chloride biodegradation pathways of Mycobacterium strain JS60. J Bacteriol 185(18):5536–5545. 10.1128/JB.185.18.5536-5545.2003 12949106 10.1128/JB.185.18.5536-5545.2003 PMC193758

[CR42] Conant BJ Jr (2004) Delineating and quantifying ground water discharge zones using streambed temperatures. Groundwater 42(2):243–257. 10.1111/j.1745-6584.2004.tb02671.x 10.1111/j.1745-6584.2004.tb02671.x 15035588

[CR43] Conant B Jr, Robinson CE, Hinton MJ, Russell HA (2019) A framework for conceptualizing groundwater-surface water interactions and identifying potential impacts on water quality, water quantity, and ecosystems. J Hydrol 574:609–627. 10.1016/j.jhydrol.2019.04.050

[CR44] Culpepper JD, Scherer MM, Robinson TC, Neumann A, Cwiertny D, Latta DE (2018) Reduction of PCE and TCE by magnetite revisited. Environ Sci Process Impacts 20(10):1340–1349. 10.1039/c8em00286j 30191930 10.1039/c8em00286j

[CR45] Cummings DE, Caccavo F Jr, Spring S, Rosenzweig RF (1999) Ferribacterium limneticum, gen. nov., sp. nov., an Fe (III)-reducing microorganism isolated from mining-impacted freshwater lake sediments. Arch Microbiol 171:183–188. 10.1007/s002030050697

[CR46] Dangeti S, McBeth JM, Roshani B, Vyskocil JM, Rindall B, Chang W (2020) Microbial communities and biogenic Mn-oxides in an on-site biofiltration system for cold Fe-(II)-and Mn (II)-rich groundwater treatment. Sci Total Environ 710:136386. 10.1016/j.scitotenv.2019.136386 31927292 10.1016/j.scitotenv.2019.136386

[CR47] Danko AS, Luo M, Bagwell CE, Brigmon RL, Freedman DL (2004) Involvement of linear plasmids in aerobic biodegradation of vinyl chloride. Appl Environ Microbiol 70(10):6092–6097. 10.1128/AEM.70.10.6092-6097.200 15466555 10.1128/AEM.70.10.6092-6097.2004 PMC522125

[CR48] Davidov Y, Jurkevitch E (2004) Diversity and evolution of *Bdellovibrio*-and-like organisms (BALOs), reclassification of *Bacteriovorax starrii* as *Peredibacter starrii* gen. nov., comb. nov., and description of the *Bacteriovorax*–*Peredibacter* clade as Bacteriovoracaceae fam. nov. Int J Syst Evol Microbiol 54(5):1439–1452. 10.1099/ijs.0.02978-0 10.1099/ijs.0.02978-0 15388693

[CR49] Dhivert E, Grosbois C, Rodrigues S, Desmet M (2015) Influence of fluvial environments on sediment archiving processes and temporal pollutant dynamics (Upper Loire River, France). Sci Total Environ 505:121–136. 10.1016/j.scitotenv.2014.09.082 25310887 10.1016/j.scitotenv.2014.09.082

[CR50] Dolinová I, Štrojsová M, Černík M, Němeček J, Macháčková J, Ševců A (2017) Microbial degradation of chloroethenes: a review. Environ Sci Pollut Res 24:13262–13283. 10.1007/s11356-017-8867-y 10.1007/s11356-017-8867-y 28378313

[CR51] Drzyzga O (2012) The strengths and weaknesses of *Gordonia*: a review of an emerging genus with increasing biotechnological potential. Crit Rev Microbiol 38(4):300–316. 10.3109/1040841X.2012.668134 22551505 10.3109/1040841X.2012.668134

[CR52] Duan JL, Sun JW, Ji MM, Ma Y, Cui ZT, Tian RK, Xu PC, Sun WL, Yuan XZ (2020) Indicatory bacteria and chemical composition related to sulfur distribution in the river-lake systems. Microbiol Res 236:126453. 10.1016/j.micres.2020.126453 32155560 10.1016/j.micres.2020.126453

[CR53] Dutta N, Usman M, Ashraf MA, Luo G, Zhang S (2022) A critical review of recent advances in the bio-remediation of chlorinated substances by microbial dechlorinators. Chemical Engineering Journal Advances. 10.1016/j.ceja.2022.100359

[CR54] Einsiedl F, Pilloni G, Ruth-Anneser B, Lueders T, Griebler C (2015) Spatial distributions of sulphur species and sulphate-reducing bacteria provide insights into sulphur redox cycling and biodegradation hot-spots in a hydrocarbon-contaminated aquifer. Geochim Cosmochim Acta 156:207–221. 10.1016/j.gca.2015.01.014

[CR55] Ellis PA, Rivett MO (2007) Assessing the impact of VOC-contaminated groundwater on surface water at the city scale. J Contam Hydrol 91(1–2):107–127. 10.1016/j.jconhyd.2006.08.015 17182150 10.1016/j.jconhyd.2006.08.015

[CR56] Engelhardt I, Piepenbrink M, Trauth N, Stadler S, Kludt C, Schulz M, Schüth C, Ternes TA (2011) Comparison of tracer methods to quantify hydrodynamic exchange within the hyporheic zone. J Hydrol 400(1–2):255–266. 10.1016/j.jhydrol.2011.01.033

[CR57] Epting J, Huggenberger P, Radny D, Hammes F, Hollender J, Page RM, Weber S, Bänninger D, Auckenthaler A (2018) Spatiotemporal scales of river-groundwater interaction–the role of local interaction processes and regional groundwater regimes. Sci Total Environ 618:1224–1243. 10.1016/j.scitotenv.2017.09.219 29111243 10.1016/j.scitotenv.2017.09.219

[CR58] Erning K, Grandel S, Dahmke A, Schäfer D (2012) Simulation of DNAPL infiltration and spreading behaviour in the saturated zone at varying flow velocities and alternating subsurface geometries. Environ Earth Sci 65:1119–1131. 10.1007/s12665-011-1361-9

[CR59] Fan X, Nie L, Chen Z, Zheng Y, Wang G, Shi K (2023) Simultaneous removal of nitrogen and arsenite by heterotrophic nitrification and aerobic denitrification bacterium *Hydrogenophaga* sp. H7. Front Microbiol 13:1103913. 10.3389/fmicb.2022.1103913 36938130 10.3389/fmicb.2022.1103913 PMC10020585

[CR60] Field JA, & Sierra R (2001) Review of scientific literature on microbial dechlorination and chlorination of key chlorinated compounds. Department of Chemical & Environmental Engineering University of Arizona, 37p

[CR61] Findlay M, Smoler DF, Fogel S, Mattes TE (2016) Aerobic vinyl chloride metabolism in groundwater microcosms by methanotrophic and etheneotrophic bacteria. Environ Sci Technol 50(7):3617–3625. 10.1021/acs.est.5b05798 26918370 10.1021/acs.est.5b05798

[CR62] Fortney NW, He S, Kulkarni A, Friedrich MW, Holz C, Boyd ES, Roden EE (2018) Stable isotope probing for microbial iron reduction in Chocolate Pots hot spring. Yellowstone National Park. Appl Environ Microbiol 84(11):e02894-17. 10.1128/AEM.02894-17 29602784 10.1128/AEM.02894-17 PMC5960972

[CR63] Franzetti A, Caredda P, Ruggeri C, La Colla P, Tamburini E, Papacchini M, Bestetti G (2009) Potential applications of surface active compounds by *Gordonia sp. *strain BS29 in soil remediation technologies. Chemosphere 75(6):801–807. 10.1016/j.chemosphere.2008.12.052 19181361 10.1016/j.chemosphere.2008.12.052

[CR64] Freitas JG, Rivett MO, Roche RS, Durrant M, Walker C, Tellam JH (2015) Heterogeneous hyporheic zone dechlorination of a TCE groundwater plume discharging to an urban river reach. Sci Total Environ 505:236–252. 10.1016/j.scitotenv.2014.09.083 25461025 10.1016/j.scitotenv.2014.09.083

[CR65] Freixa A, Rubol S, Carles-Brangarí A, Fernàndez-Garcia D, Butturini A, Sanchez-Vila X, Romaní AM (2016) The effects of sediment depth and oxygen concentration on the use of organic matter: an experimental study using an infiltration sediment tank. Sci Total Environ 540:20–31. 10.1016/j.scitotenv.2015.04.007 25900223 10.1016/j.scitotenv.2015.04.007

[CR66] Fuerst JA (2017) Planctomycetes—new models for microbial cells and activities. Microbial resources. Academic Press, pp 1–27. 10.1016/B978-0-12-804765-1.00001-1

[CR67] Fukunaga Y, Kurahashi M, Sakiyama Y, Ohuchi M, Yokota A, Harayama S (2009) *Phycisphaera mikurensis* gen. nov., sp. nov., isolated from a marine alga, and proposal of Phycisphaeraceae fam. nov., Phycisphaerales ord. nov. and Phycisphaerae classis nov. in the phylum Planctomycetes. J Gen Appl Microbiol 55(4):267–275. 10.2323/jgam.55.267 19700920 10.2323/jgam.55.267

[CR68] Futamata H, Nagano Y, Watanabe K, Hiraishi A (2005) Unique kinetic properties of phenol-degrading *Variovorax* strains responsible for efficient trichloroethylene degradation in a chemostat enrichment culture. Appl Environ Microbiol 71(2):904–911 15691947 10.1128/AEM.71.2.904-911.2005 PMC546690

[CR69] Futami, R, Muñoz-Pomer A, Viu JM, Domínguez-Escribà, L., Covelli L, Bernet GP, J.M. S, Moya A, Llorens C (2011) GPRO: the professional tool for management, functional analysis and annotation of omic sequences and databases. Biotechvana Bioinforma 1–5. http://www.biotechvana.com/software/gpro

[CR70] Galia T, Škarpich V, Vala O (2022) Trees and shrubs as components of the storage of coarse particulate organic matter and instream wood in Mediterranean intermittent streams. Ecohydrol Hydrobiol 22(4):553–564. 10.1016/j.ecohyd.2022.08.003

[CR71] Gao AJ, Zhu T, Liu C, Zhang J, Gao J, Zhang J, Cai M, Li Y (2020) Ammonium removal characteristics of heterotrophic nitrifying bacterium *Pseudomonas stutzeri* GEP-01 with potential for treatment of ammonium-rich wastewater. Bioprocess Biosyst Eng 43:959–969. 10.1007/s00449-020-02292-x 31980902 10.1007/s00449-020-02292-x

[CR72] Ge Q, Yue X, Wang G (2015) Simultaneous heterotrophic nitrification and aerobic denitrification at high initial phenol concentration by isolated bacterium *Diaphorobacter sp.* PD-7. Chin J Chem Eng 23(5):835–841. 10.1016/j.cjche.2015.02.001

[CR73] Geng C, Xiaosi S, Liu Y, Shida Z (2021) Effect of riverbed sediment flushing and clogging on river-water infiltration rate: a case study in the Second Songhua River. Northeast China. Hydrogeol J 29(2):551–565. 10.1007/s10040-020-02218-7

[CR74] Ghezzi D, Filippini M, Cappelletti M, Firrincieli A, Zannoni D, Gargini A, Fedi S (2021) Molecular characterization of microbial communities in a peat-rich aquifer system contaminated with chlorinated aliphatic compounds. Environ Sci Pollut Res 28:23017–23035. 10.1007/s11356-020-12236-3 10.1007/s11356-020-12236-3 33438126

[CR75] Giddings CG, Liu F, Gossett JM (2010) Microcosm assessment of *Polaromonas sp.* JS666 as a bioaugmentation agent for degradation of cis-1, 2-dichloroethene in aerobic, subsurface environments. Groundwater Monit Remediat 30(2):106–113. 10.1111/j.1745-6592.2010.01283.x

[CR76] Gros M, Catalán N, Mas-Pla J, Čelić M, Petrović M, Farré MJ (2021) Groundwater antibiotic pollution and its relationship with dissolved organic matter: identification and environmental implications. Environ Pollut 289:117927. 10.1016/j.envpol.2021.117927 34426209 10.1016/j.envpol.2021.117927

[CR77] Guha N, Loomis D, Grosse Y, Lauby-Secretan B, El Ghissassi F, Bouvard V, Benbrahim-Tallaa L, Baan R, Mattock H, Straif K (2012) International Agency for Research on Cancer Monograph Working Group carcinogenicity of trichloroethylene, tetrachloroethylene, some other chlorinated solvents, and their metabolites. Lancet Oncol 13(12):1192–1193. 10.1016/s1470-2045(12)70485-0 23323277 10.1016/s1470-2045(12)70485-0

[CR78] Haggerty R, Wondzell SM, Johnson MA (2002) Power-law residence time distribution in the hyporheic zone of a 2nd-order mountain stream. Geophys Res Lett 29(13):18–1. 10.1029/2002GL014743

[CR79] Hahn MW, Kasalický V, Jezbera J, Brandt U, Jezberova J, Šimek K (2010) *Limnohabitans curvus* gen. nov., sp. nov., a planktonic bacterium isolated from a freshwater lake. Int J Syst Evol Microbiol 60(6):1358–1365. 10.1099/ijs.0.013292-0 19671731 10.1099/ijs.0.013292-0 PMC3091418

[CR80] Hampton TB, Zarnetske JP, Briggs MA, Dehkordy FM, Singha K, Day-Lewis FD, MahmoodPoor Dehkordy F, Harvey JW, Chowdhury SR, Lane JW (2020) Experimental shifts of hydrologic residence time in a sandy urban stream sediment–water interface alter nitrate removal and nitrous oxide fluxes. Biogeochem 149(2):195–219. 10.1007/s10533-020-00674-7

[CR81] Haston ZC, Mccarty PL (1999) Chlorinated ethene half-velocity coefficients (KS) for reductive dehalogenation. Environ Sci Technol 33:223–226. 10.1021/es9805876

[CR82] Hartwell SI, Alden RW, Wright DA, Ailstock S, Kerhin R (2000) Correlation of measures of ambient toxicity and fish community diversity in a Chesapeake Bay tributary, Maryland, USA: A biological, chemical, and geological assessment. Environ Toxicol Chem 19(7):1753–1763. 10.1002/etc.5620190708

[CR83] He YT, Wilson JT, Su C, Wilkin RT (2015) Review of abiotic degradation of chlorinated solvents by reactive iron minerals in aquifers. Groundw Monit Remediat 35(3):57–75. 10.1111/gwmr.12111

[CR84] Hellal J, Joulian C, Urien C, Ferreira S, Denonfoux J, Hermon L, Vuilleumier S, Imfeld G (2021) Chlorinated ethene biodegradation and associated bacterial taxa in multi-polluted groundwater: Insights from biomolecular markers and stable isotope analysis. Sci Total Environ 763:142950. 10.1016/j.scitotenv.2020.142950 33127155 10.1016/j.scitotenv.2020.142950

[CR85] Hertle S, De Boni N, Schell H, Tiehm A (2023) Electrochemical biostimulation of aerobic metabolic TCE degradation in a bioaugmentation approach. Environ Sci Pollut Res 30(49):107673–107680. 10.1007/s11356-023-29839-1 10.1007/s11356-023-29839-1 PMC10611883 37735338

[CR86] Hespell RB, Paster BJ, Macke TJ, Woese CR (1984) The origin and phylogeny of the bdellovibrios. Syst Appl Microbiol 5(2):196–203. 10.1016/S0723-2020(84)80020-X

[CR87] Höhne A, Müller BM, Schulz H, Dara R, Posselt M, Lewandowski J, McCallum JL (2022) Fate of trace organic compounds in the hyporheic zone: Influence of microbial metabolism. Water Res 224:119056. 10.1016/j.watres.2022.119056 36126632 10.1016/j.watres.2022.119056

[CR88] Holliger C, Schumacher W (1994) Reductive dehalogenation as a respiratory process. Antonie Van Leeuwenhoek 66:239–246. 10.1007/BF00871642 7747935 10.1007/BF00871642

[CR89] Hong C, Zhang J, Liu T, Teng W, Fu R, Qiu Y (2023) Simultaneous and long-term effective immobilization of lead, cadmium and arsenic in multi-contaminated soil by ferrihydrite-supported animal-derived biochar. J Environ Chem Eng 11(3):109989. 10.1016/j.jece.2023.109989

[CR90] Horn MA, Ihssen J, Matthies C, Schramm A, Acker G, Drake HL (2005) *Dechloromonas denitrificans* sp. nov., *Flavobacterium denitrificans* sp. nov., *Paenibacillus anaericanus* sp. nov. and *Paenibacillus terrae* strain MH72, N2O-producing bacteria isolated from the gut of the earthworm *Aporrectodea caliginosa*. Int J Syst Evol Microbiol 55(3):1255–1265. 10.1099/ijs.0.63484-0 15879265 10.1099/ijs.0.63484-0

[CR91] Hsu CY, Chiang HC, Shie RH, Ku CH, Lin TY, Chen MJ, Chen NT, Chen YC (2018) Ambient VOCs in residential areas near a large-scale petrochemical complex: Spatiotemporal variation, source apportionment and health risk. Environ Pollut 240:95–104. 10.1016/j.envpol.2018.04.076 29730422 10.1016/j.envpol.2018.04.076

[CR92] Hug LA, Maphosa F, Leys D, Löffler FE, Smidt H, Edwards EA, Adrian L (2013) Overview of organohalide-respiring bacteria and a proposal for a classification system for reductive dehalogenases. Philos Trans R Soc Lond B Biol Sci 368(1616):20120322. 10.1098/rstb.2012.0322 23479752 10.1098/rstb.2012.0322 PMC3638463

[CR93] Huijbregts MA, Rombouts LJ, Ragas AM, van de Meent D (2005) Human-toxicological effect and damage factors of carcinogenic and noncarcinogenic chemicals for life cycle impact assessment. Integr Environ Assess Manag 1(3):181–244. 10.1897/2004-007R.1 16639884 10.1897/2004-007r.1

[CR94] Irgens RL, Gosink JJ, Staley JT (1996) *Polaromonas vacuolata* gen. nov., sp. nov., a psychrophilic, marine, gas vacuolate bacterium from Antarctica. Int J Syst Evol Microbiol 46(3):822–826. 10.1099/00207713-46-3-822 10.1099/00207713-46-3-822 8782696

[CR95] Itoh T, Yamanoi K, Kudo T, Ohkuma M, Takashina T (2011) *Aciditerrimonas ferrireducens* gen. nov., sp. nov., an iron-reducing thermoacidophilic actinobacterium isolated from a solfataric field. Int J Syst Evol Microbiol 61(6):1281–1285. 10.1099/ijs.0.023044-0 20639230 10.1099/ijs.0.023044-0

[CR96] Jennings LK, Chartrand MM, Lacrampe-Couloume G, Lollar BS, Spain JC, Gossett JM (2009) Proteomic and transcriptomic analyses reveal genes upregulated by cis-dichloroethene in *Polaromonas* sp. strain JS666. Appl Environ Microbiol 75(11):3733–3744. 10.1128/AEM.00031-09 19363075 10.1128/AEM.00031-09 PMC2687319

[CR97] Jin H, Huo L, Yang Y, Lv Y, Wang J, Maillard J, Holliger C, Löffler FE, Yan J (2023) *Sulfurospirillum diekertiae* sp. nov., a tetrachloroethene-respiring bacterium isolated from contaminated soil. Int J Syst Evol Microbiol 73(2):005693. 10.1099/ijsem.0.005693 10.1099/ijsem.0.005693 36735579

[CR98] Jing R, Fusi S, Kjellerup BV (2018) Remediation of polychlorinated biphenyls (PCBs) in contaminated soils and sediment: state of knowledge and perspectives. Front Environ Sci 6(79):79. 10.3389/fenvs.2018.00079

[CR99] Jørgensen BB, Findlay AJ, Pellerin A (2019) The biogeochemical sulfur cycle of marine sediments. Front Microbiol 10:849. 10.3389/fmicb.2019.00849 31105660 10.3389/fmicb.2019.00849 PMC6492693

[CR100] Joseph V, Chellappan G, Aparajitha S, Ramya RN, Vrinda S, Rejish Kumar VJ, Bright Singh IS (2021) Molecular characterization of bacteria and archaea in a bioaugmented zero-water exchange shrimp pond. SN Appl Sci 3:1–20. 10.1007/s42452-021-04392-z

[CR101] Jroundi F, Descostes M, Povedano-Priego C, Sánchez-Castro I, Suvannagan V, Grizard P, Merroun ML (2020) Profiling native aquifer bacteria in a uranium roll-front deposit and their role in biogeochemical cycle dynamics: insights regarding in situ recovery mining. Sci Total Environ 721:137758. 10.1016/j.scitotenv.2020.137758 32179349 10.1016/j.scitotenv.2020.137758

[CR102] Jugder BE, Ertan H, Bohl S, Lee M, Marquis CP, Manefield M (2016) Organohalide respiring bacteria and reductive dehalogenases: key tools in organohalide bioremediation. Front Microbiol 7:249. 10.3389/fmicb.2016.00249 26973626 10.3389/fmicb.2016.00249 PMC4771760

[CR103] Kale V, Björnsdóttir SH, Friðjónsson ÓH, Pétursdóttir SK, Omarsdottir S, Hreggviðsson GO (2013) *Litorilinea aerophila* gen. nov., sp. nov., an aerobic member of the class Caldilineae, phylum Chloroflexi, isolated from an intertidal hot spring. Int J Syst Evol Microbiol 63(Pt_3):1149–1154. 10.1099/ijs.0.044115-0 10.1099/ijs.0.044115-0 22771681

[CR104] Kasalický V, Jezbera J, Šimek K, Hahn MW (2010) *Limnohabitans planktonicus* sp nov and *Limnohabitans parvus* sp nov, planktonic betaproteobacteria isolated from a freshwater reservoir, and emended description of the genus *Limnohabitans*. Int J Syst Evol Microbiol 60(12):2710–2714. 10.1099/ijs.0.018952-0 20061501 10.1099/ijs.0.018952-0 PMC3091486

[CR105] Khardenavis AA, Kapley A, Purohit HJ (2007) Simultaneous nitrification and denitrification by diverse *Diaphorobacter* sp. Appl Microbiol Biotechnol 77:403–409. 10.1007/s00253-007-1176-5 17876578 10.1007/s00253-007-1176-5

[CR106] Kim KK, Lee CS, Kroppenstedt RM, Stackebrandt E, Lee ST (2003) *Gordonia sihwensis* sp. nov., a novel nitrate-reducing bacterium isolated from a wastewater-treatment bioreactor. Int J Syst Evol Microbiol 53(5):1427–1433. 10.1099/ijs.0.02224-0 13130028 10.1099/ijs.0.02224-0

[CR107] Kim E-S, Nomura I, Hasegawa Y, Takamizawa K (2006) Characterization of a newlyisolatedcis-1, 2-dichloroethylene and aliphatic compound-degrading bacterium, *Clostridium* sp. strain KYT-1. Biotechnol Bioprocess Eng 11:553–556. 10.1007/BF02932083

[CR108] Koch J, Nowak W (2015) Predicting DNAPL mass discharge and contaminated site longevity probabilities: conceptual model and high-resolution stochastic simulation. Water Resour Res 51(2):806–831. 10.1002/2014WR015478

[CR109] Kotik M, Davidová A, Voříšková J, Baldrian P (2013) Bacterial communities in tetrachloroethene-polluted groundwaters: a case study. Sci Total Environ 454:517–527. 10.1016/j.scitotenv.2013.02.082 23567172 10.1016/j.scitotenv.2013.02.082

[CR110] Krause S, Hannah DM, Fleckenstein JH, Heppell CM, Kaeser D, Pickup R, Pinay G, Robertson AL, Wood PJ (2011) Inter-disciplinary perspectives on processes in the hyporheic zone. Ecohydrology 4(4):481–499. 10.1002/eco.176

[CR111] Krüger M, Chaudhari N, Thamdrup B, Overholt WA, Bristow LA, Taubert M, Küsel K, Jehmlich N, von Bergen M, & Herrmann M (2023) Differential contribution of nitrifying prokaryotes to groundwater nitrification. The ISME J 1–11. 10.1038/s41396-023-01471-4 10.1038/s41396-023-01471-4 PMC10504367 37422599

[CR112] Kulichevskaya IS, Baulina OI, Bodelier PL, Rijpstra WIC, Damste JSS, Dedysh SN (2009) *Zavarzinella formosa* gen. nov., sp. nov., a novel stalked, Gemmata-like planctomycete from a Siberian peat bog. Int J Syst Evol Microbiol 59(2):357–364. 10.1099/ijs.0.002378-0 19196778 10.1099/ijs.0.002378-0

[CR113] Kulichevskaya IS, Ivanova AO, Belova SE, Baulina OI, Bodelier PL, Rijpstra WIC, Sinninghe Damsté JS, Zavarzin GA, Dedysh SN (2007) *Schlesneria paludicola* gen. nov., sp. nov., the first acidophilic member of the order Planctomycetales, from *Sphagnum*-dominated boreal wetlands. Int J Syst Evol Microbiol 57(11):2680–2687. 10.1099/ijs.0.65157-0 17978240 10.1099/ijs.0.65157-0

[CR114] Larned ST, Unwin MJ, Boustead NC (2015) Ecological dynamics in the riverine aquifers of a gaining and losing river. Freshw Sci 34(1):245–262. 10.1086/678350

[CR115] Lee DW, Ahn Y, Pandi K, Park J, Yun ST, Jang M, Choi J (2021) Evaluation of natural attenuation-potential and biogeochemical analysis in nitrate contaminated bedrock aquifers by carbon source injection. Sci Total Environ 780:146459. 10.1016/j.scitotenv.2021.146459 34030323 10.1016/j.scitotenv.2021.146459

[CR116] Lee MS, Lee KK, Hyun Y, Clement TP, Hamilton D (2006) Nitrogen transformation and transport modeling in groundwater aquifers. Ecol Model 192(1–2):143–159. 10.1016/j.ecolmodel.2005.07.013

[CR117] Lee JH, Lim CS, Lee MG, Kim HS (2015) Evaluation of a rapid immunochromatographic treponemal antibody test comparing the *Treponema pallidum* particle agglutination assay. J Clin Lab Anal 29(5):383–386. 10.1002/jcla.21783 25385043 10.1002/jcla.21783 PMC6807139

[CR118] Lee J, Park B, Woo SG, Lee J, Park J (2014) *Prosthecobacter algae* sp. nov., isolated from activated sludge using algal metabolites. Int J Syst Evol Microbiol 64(Pt_2):663–667. 10.1099/ijs.0.052787-0 10.1099/ijs.0.052787-0 24170774

[CR119] Levett PN, Morey RE, Galloway R, Steigerwalt AG, Ellis WA (2005) Reclassification of *Leptospira parva* Hovind-Hougen et al 1982 as *Turneriella parva* gen nov, comb nov. Int J Syst Evol Microbiol 55(4):1497–1499. 10.1099/ijs.0.63088-0 10.1099/ijs.0.63088-0 16014471

[CR120] Li X, Zhang W, Liu T, Chen L, Chen P, Li F (2016) Changes in the composition and diversity of microbial communities during anaerobic nitrate reduction and Fe (II) oxidation at circumneutral pH in paddy soil. Soil Biol Biochem 94:70–79. 10.1016/j.soilbio.2015.11.013

[CR121] Li C, Xue C, Ouyang W, Liu M, Sun Y, Liu H (2023) Identification and synergetic mechanism of TCE, H2 and O2 metabolic microorganisms in the joint H2/O2 system. Sci Total Environ 879:163026. 10.1016/j.scitotenv.2023.163026 36965730 10.1016/j.scitotenv.2023.163026

[CR122] Liang B, Yun H, Kong D, Ding Y, Li X, Vangnai AS, Wang A (2020) Bioaugmentation of triclocarban and its dechlorinated congeners contaminated soil with functional degraders and the bacterial community response. Environ Res 180(108840):108840. 10.1016/j.envres.2019.108840 31654905 10.1016/j.envres.2019.108840

[CR123] Lihl C, Douglas LM, Franke S, P´erez-de-Mora A, Meyer AH, Daubmeier M, Edwards EA, Nijenhuis I, Sherwood Lollar B, Elsner M (2019) Mechanistic dichotomy in bacterial trichloroethene dechlorination revealed by carbon and chlorine isotope effects. Environ Sci Technol 53(8):4245–4254. 10.1021/acs.est.8b06643 30857389 10.1021/acs.est.8b06643

[CR124] Liu QM, Ten LN, Yu HS, Jin FX, Im WT, Lee ST (2008) *Emticicia ginsengisoli* sp. nov., a species of the family ‘Flexibacteraceae’isolated from soil of a ginseng field. Int J Syst Evol Microbiol 58(5):1100–1105. 10.1099/ijs.0.65386-0 18450696 10.1099/ijs.0.65386-0

[CR125] Liu X, Wu Y, Wilson FP, Yu K, Lintner C, Cupples AM, Mattes TE (2018) Integrated methodological approach reveals microbial diversity and functions in aerobic groundwater microcosms adapted to vinyl chloride. FEMS Microbiol Ecol 94(9):fiy124. 10.1093/femsec/fiy124 10.1093/femsec/fiy124 29945195

[CR126] Löffler FE, Ritalahti KM, Zinder SH (2013) *Dehalococcoides* and reductive dichlorination of chlorinated solvents. Bioaugmentation for groundwater remediation. Springer, New York, NY, pp 39–88. 10.1007/978-1-4614-4115-1_2

[CR127] Lohner ST, Tiehm A (2009) Application of electrolysis to stimulate microbial reductive PCE dechlorination and oxidative VC biodegradation. Environ Sci Technol 43:7098–7104. 10.1021/es900835d 19806748 10.1021/es900835d

[CR128] Lourenço KS, Cassman NA, Pijl AS, Van Veen JA, Cantarella H, Kuramae EE (2018) *Nitrosospira* sp. govern nitrous oxide emissions in a tropical soil amended with residues of bioenergy crop. Front Microbiol 9:674. 10.3389/fmicb.2018.00674 29692763 10.3389/fmicb.2018.00674 PMC5902487

[CR129] Lovley DR, Giovannoni SJ, White DC, Champine JE, Phillips EJP, Gorby YA, Goodwin S (1993) *Geobacter metallireducens* gen. nov. sp. nov., a microorganism capable of coupling the complete oxidation of organic compounds to the reduction of iron and other metals. Arch Microbiol 159:336–344. 10.1007/BF00290916 8387263 10.1007/BF00290916

[CR130] Luo SG, Chen SC, Cao WZ, Lin WH, Sheu YT, Kao CM (2019) Application of γ-PGA as the primary carbon source to bioremediate a TCE-polluted aquifer: a pilot-scale study. Chemosphere 237:124449. 10.1016/j.chemosphere.2019.124449 31376698 10.1016/j.chemosphere.2019.124449

[CR131] Luo S, Zhen Z, Zhu X, Ren L, Wu W, Zhang W, Chen Y, Zhang D, Song Z, Lin Z, Liang YQ (2021) Accelerated atrazine degradation and altered metabolic pathways in goat manure assisted soil bioremediation. Ecotoxicol Environ Saf 221:112432. 10.1016/j.ecoenv.2021.112432 34166937 10.1016/j.ecoenv.2021.112432

[CR132] Mariotti A, Germon JC, Hubert P, Kaiser P, Letolle R, Tardieux A, Tardieux P (1981) Experimental determination of nitrogen kinetic isotope fractionation: some principles; illustration for the denitrification and nitrification processes. Plant Soil 62:413–430. 10.1007/BF02374138

[CR133] Mattes TE, Alexander AK, Richardson PM, Munk AC, Han CS, Stothard P, Coleman NV (2008) The genome of *Polaromonas* sp. strain JS666: insights into the evolution of a hydrocarbon-and xenobiotic-degrading bacterium, and features of relevance to biotechnology. Appl Environ Microbiol 74(20):6405–6416. 10.1128/AEM.00197-08 18723656 10.1128/AEM.00197-08 PMC2570305

[CR134] Mattes TE, Alexander AK, Coleman NV (2010) Aerobic biodegradation of the chloroethenes: pathways, enzymes, ecology, and evolution. FEMS Microbiol Rev 34(4):445–475. 10.1111/j.1574-6976.2010.00210.x 20146755 10.1111/j.1574-6976.2010.00210.x

[CR135] Maymó-Gatell X, Chien Y-t, Gossett JM, Zinder SH (1997) Isolation of a bacterium that reductively dechlorinates tetrachloroethene to ethene. Sci 276:1568–1571. 10.1126/science.276.5318.1568 10.1126/science.276.5318.1568 9171062

[CR136] Maymó-Gatell X, Nijenhuis I, Zinder SH (2001) Reductive dechlorination of cis-1, 2-dichloroethene and vinyl chloride by “*Dehalococcoides ethenogenes*.” Environ Sci Technol 35(3):516–521. 10.1021/es001285i 11351722 10.1021/es001285i

[CR137] McCarty PL, Smith DP (1986) Anaerobic wastewater treatment. Environ Sci Technol 20(12):1200–1206

[CR138] McGuire TM, Newell CJ, Looney BB, Vangelas KM, Sink CH (2004) Historical analysis of monitored natural attenuation: a survey of 191 chlorinated solvent sites and 45 solvent plumes. Remediat J 15(1):99–112. 10.1002/rem.20036

[CR139] McMahon PB, Chapelle FH (2008) Redox processes and water quality of selected principal aquifer systems. Ground Water 46(2):259–271. 10.1111/j.1745-6584.2007.00385.x 18307432 10.1111/j.1745-6584.2007.00385.x

[CR140] McKnight US, Rasmussen JJ, Kronvang B, Bjerg PL, Binning PJ (2012) Integrated assessment of the impact of chemical stressors on surface water ecosystems. Sci Total Environ 427:319–331. 10.1016/j.scitotenv.2012.04.011 22554536 10.1016/j.scitotenv.2012.04.011

[CR141] Mergaert J, Lednicka D, Goris J, Cnockaert MC, De Vos P, Swings J (2003) Taxonomic study of *Cellvibrio* strains and description of *Cellvibrio ostraviensis* sp. nov., *Cellvibrio fibrivorans* sp. nov. and *Cellvibrio gandavensis* sp. nov. Int J Syst Evol Microbiol 53(2):465–471. 10.1099/ijs.0.02316-0 12710614 10.1099/ijs.0.02316-0

[CR142] Meyer JL, Paul MJ, Taulbee WK (2005) Stream ecosystem function in urbanizing landscapes. J N Am Benthol Soc 24(3):602–612. 10.1899/04-021.1

[CR143] Miller LG, Baesman SM, Kirshtein J, Voytek MA, Oremland RS (2013) A biogeochemical and genetic survey of acetylene fermentation by environmental samples and bacterial isolates. Geomicrobiol J 30(6):501–516. 10.1080/01490451.2012.732662

[CR144] Mojarrad BB, Betterle A, Singh T, Olid C, Wörman A (2019) The effect of stream discharge on hyporheic exchange. Water 11(7):1436. 10.3390/w11071436

[CR145] Monciardini P, Cavaletti L, Schumann P, Rohde M, Donadio S (2003) *Conexibacter woesei* gen. nov., sp. nov., a novel representative of a deep evolutionary line of descent within the class Actinobacteria. Int J Syst Evol Microbiol 53(2):569–576. 10.1099/ijs.0.02400-0 12710628 10.1099/ijs.0.02400-0

[CR146] Moran MJ, Zogorski JS, Squillace PJ (2007) Chlorinated solvents in groundwater of the United States. Environ Sci Technol 41(1):74–81. 10.1021/es061553y 17265929 10.1021/es061553y

[CR147] Morrison RD, Murphy BL (2015) Chlorinated solvents: a forensic evaluation. Royal Society of Chemistry, Dorchester. 10.1039/9781849737265

[CR148] Morrissy JG, Currell MJ, Reichman SM, Surapaneni A, Megharaj M, Crosbie ND, Hirth D, Aquilina S, Rajendram W, Ball AS (2022) The variation in groundwater microbial communities in an unconfined aquifer contaminated by multiple nitrogen contamination sources. Water 14(4):613. 10.3390/w14040613

[CR149] Mueller BM, Schulz H, Danczak RE, Putschew A, Lewandowski J (2021) Simultaneous attenuation of trace organics and change in organic matter composition in the hyporheic zone of urban streams. Sci Rep 11(1):4179. 10.1038/s41598-021-83750-8 33603043 10.1038/s41598-021-83750-8 PMC7892836

[CR150] Munoz-Palazon B, Mikola A, Rosa-Masegosa A, Vilchez-Vargas R, Link A, Gonzalez-Lopez J, Gonzalez-Martinez A (2022) Novel application of aerobic granular biofilm systems for treating nitrate-polluted groundwater at low temperature: microbial community and performance. J Environ Chem Eng 10(3):107818. 10.1016/j.jece.2022.107818

[CR151] National Research Council (1999) Improving management of persistent of contaminants. Groundwater and soil cleanup. National Academic Press, Washington, DC, pp 113–174

[CR152] Němeček J, Marková K, Špánek R, Antoš V, Kozubek P, Lhotský O, Černík M (2020) Hydrochemical conditions for aerobic/anaerobic biodegradation of chlorinated ethenes—a multi-site assessment. Water 12(2):322. 10.3390/w12020322

[CR153] Němeček J, Steinová J, Špánek R, Pluhař T, Pokorný P, Najmanová P, Knytl V, Černík M (2018) Thermally enhanced in situ bioremediation of groundwater contaminated with chlorinated solvents–a field test. Sci Total Environ 622:743–755. 10.1016/j.scitotenv.2017.12.047 29223901 10.1016/j.scitotenv.2017.12.047

[CR154] Nijenhuis I, Kuntze K (2016) Anaerobic microbial dehalogenation of organohalides—state of the art and remediation strategies. Curr Opin Biotechnol 38:33–38. 10.1016/j.copbio.2015.11.009 10.1016/j.copbio.2015.11.009 26773757

[CR155] Norton JM, Low JM, Klotz MG (1996) The gene encoding ammonia monooxygenase subunit A exists in three nearly identical copies in *Nitrosospira* sp. NpAV. FEMS Microbiol Lett 139(2–3):181–188. 10.1016/0378-1097(96)00139-5 8674986 10.1111/j.1574-6968.1996.tb08200.x

[CR156] Nsir K, Schäfer G, di Chiara Roupert R, Mercury L (2018) Pore scale modelling of DNAPL migration in a water–saturated porous medium. J Contam Hydrol 215:39–50. 10.1016/j.jconhyd.2018.07.001 30060891 10.1016/j.jconhyd.2018.07.001

[CR157] O’Connor AE, Luek JL, McIntosh H, Beck AJ (2015) Geochemistry of redox-sensitive trace elements in a shallow subterranean estuary. Mar Chem 172:70–81. 10.1016/j.marchem.2015.03.001

[CR158] O’Sullivan LA, Rinna J, Humphreys G, Weightman AJ, Fry JC (2005) *Fluviicola taffensis* gen. nov., sp. nov., a novel freshwater bacterium of the family Cryomorphaceae in the phylum ‘Bacteroidetes.’ Int J Syst Evol Microbiol 55(5):2189–2194. 10.1099/ijs.0.63736-0 16166730 10.1099/ijs.0.63736-0

[CR159] Ottosen CB, Rønde V, McKnight US, Annable MD, Broholm MM, Devlin JF, Bjerg PL (2020) Natural attenuation of a chlorinated ethene plume discharging to a stream: integrated assessment of hydrogeological, chemical and microbial interactions. Water Res 186:116332. 10.1016/j.watres.2020.116332 32871289 10.1016/j.watres.2020.116332

[CR160] Paix B, Ezzedine JA, Jacquet S (2019) Diversity, dynamics, and distribution of *Bdellovibrio* and like organisms in perialpine lakes. Appl Environ Microbiol 85(6):e02494-18. 10.1128/AEM.02494-18 30635378 10.1128/AEM.02494-18 PMC6414362

[CR161] Pan Y, Yang J, Jia Y, Xu Z (2016) Experimental study on non-aqueous phase liquid multiphase flow characteristics and controlling factors in heterogeneous porous media. Environ Earth Sci 75:1–13. 10.1007/s12665-015-4888-3

[CR162] Pankow JF, Cherry JA (1996) Dense chlorinated solvents and other DNAPLs in groundwater: history, behavior, and remediation. Waterloo Press, Portland, Oregon

[CR163] Patterson BM, Lee M, Bastow TP, Wilson JT, Donn MJ, Furness A, Goodwin B, Manefield M (2016) Concentration effects on biotic and abiotic processes in the removal of 1, 1, 2-trichloroethane and vinyl chloride using carbon-amended ZVI. J Contam Hydrol 188:1–11. 10.1016/j.jconhyd.2016.02.004 26934432 10.1016/j.jconhyd.2016.02.004

[CR164] Payne KAP, Quezada CP, Fisher K, Dunstan MS, Collins FA, Sjuts H, Levy C, Hay S, Rigby SEJ, Leys D (2014) Reductive dehalogenase structure suggests a mechanism for B12-dependent dehalogenation. Nature 517(7535):513–516. 10.1038/nature13901 25327251 10.1038/nature13901 PMC4968649

[CR165] Peiffer S, Kappler A, Haderlein SB, Schmidt C, Byrne JM, Kleindienst S, Vogt C, Richnow HH, Obst M, Angenent LT, Bryce C, McCammon C, Planer-Friedrich B (2021) A biogeochemical–hydrological framework for the role of redox-active compounds in aquatic systems. Nat Geosci 14(5):264–272. 10.1038/s41561-021-00742-z

[CR166] Peng I, Goris T, Lu Y, Nijsse B, Burrichter A, Schleheck D, Koehorst JJ, Liu J, Sipkema D, Sinninghe Damste JS, Stams AJM, Häggblom MM, Smidt H, Atashgahi S (2020) Organohalide-respiring *Desulfoluna* species isolated from marine environments. ISME J 14(3):815–827. 10.1038/s41396-019-0573-y 31896791 10.1038/s41396-019-0573-y PMC7031245

[CR167] Peterson DM, Miller D, Dander D, Kautsky M, Nofchissey J (2016) Groundwater remediation in a flood plain aquifer at Shiprock, New Mexico (Paper 16097). In: Proceedings of the waste management symposium 2016 (WM2016). WM Symposia, Phoenix, AZ, USA, OSTI ID 22837990

[CR168] Pham HT, Suto K, Inoue C (2009) Trichloroethylene transformation in aerobic pyrite suspension: pathways and kinetic modeling. Environ Sci Technol 43:6744–6749. 10.1021/es900623u 19764244 10.1021/es900623u

[CR169] Phillips E, Bergquist BA, Chartrand MM, Chen W, Edwards EA, Elsner M, Gilevska T, Hirschorn S, Horst A, Lacrampe-Couloume G, Mancini SA, McKelvie J, Morrill PL, Sullivan Ojeda A, Slater GF, Sleep BE, De Vera J, Warr O, Passeport E (2022) Compound specific isotope analysis in hydrogeology. J Hydrol 128588. 10.1016/j.jhydrol.2022.128588

[CR170] Piazzon MC, Naya-Català F, Simó-Mirabet P, Picard-Sánchez A, Roig FJ, Calduch-Giner JA, SitjàBobadilla A, Pérez-Sánchez J (2019) Sex, age, and bacteria: how the intestinal microbiota is modulated in a protandrous hermaphrodite fish. Front Microbiol 10:2512. 10.3389/fmicb.2019.02512 31736931 10.3389/fmicb.2019.02512 PMC6834695

[CR171] Pinardi M, Soana E, Severini E, Racchetti E, Celico F, Bartoli M (2022) Agricultural practices regulate the seasonality of groundwater-river nitrogen exchanges. Agric Water Manag 273:107904. 10.1016/j.agwat.2022.107904

[CR172] Puigserver D, Cortés A, Viladevall M, Nogueras X, Parker BL, Carmona JM (2014) Processes controlling the fate of chloroethenes emanating from DNAPL aged sources in river–aquifer contexts. J Contam Hydrol 168:25–40. 10.1016/j.jconhyd.2014.09.005 25278314 10.1016/j.jconhyd.2014.09.005

[CR173] Puigserver D, Herrero J, Torres M, Cortés A, Nijenhuis I, Kuntze K, Parker BL, Carmona JM (2016) Reductive dechlorination in recalcitrant sources of chloroethenes in the transition zone between aquifers and aquitards. Environ Sci Pollut Res 23:18724–18741. 10.1007/s11356-016-7068-4 10.1007/s11356-016-7068-4 27314420

[CR174] Puigserver D, Herrero J, Carmona JM (2022) Nitrate removal by combining chemical and biostimulation approaches using micro-zero valent iron and lactic acid. Sci Total Environ 843:156841. 10.1016/j.scitotenv.2022.156841 35750160 10.1016/j.scitotenv.2022.156841

[CR175] Puigserver D, Herrero J, Carmona JM (2023) Mobilization pilot test of PCE sources in the transition zone to aquitards by combining mZVI and biostimulation with lactic acid. Sci Total Environ 877:162751. 10.1016/j.scitotenv.2023.162751 36921871 10.1016/j.scitotenv.2023.162751

[CR176] Qiu L, Guo X, Liang Z, Lu Q, Wang S, Shim H (2023) Uncovering the metabolic pathway of novel *Burkholderia sp.* for efficient triclosan degradation and implication: insight from exogenous bioaugmentation and toxicity pressure. Environ Pollut 334:122111. 10.1016/j.envpol.2023.122111 37392866 10.1016/j.envpol.2023.122111

[CR177] Reguera G, Kashefi K (2019) The electrifying physiology of *Geobacter* bacteria, 30 years on. Adv Microb Physiol 74:1–96. 10.1016/bs.ampbs.2019.02.007 31126529 10.1016/bs.ampbs.2019.02.007

[CR178] Rivett MO, Dearden RA, Wealthall GP (2014) Architecture, persistence and dissolution of a 20 to 45 year old trichloroethene DNAPL source zone. J Contam Hydrol 170:95–115. 10.1016/j.jconhyd.2014.09.008 25444120 10.1016/j.jconhyd.2014.09.008

[CR179] Romani AM, Guasch H, & Balaguer MD (2016) Aquatic biofilms. Ecology, water quality and wastewater treatment Norfolk (UK): Caister Academic Press. USA. 10.21775/9781910190173

[CR180] Rowe BL, Toccalino PL, Moran MJ, Zogorski JS, Price CV (2007) Occurrence and potential human-health relevance of volatile organic compounds in drinking water from domestic wells in the United States. Environ Health Perspect 115(11):1539–1546. 10.1289/ehp.10253 18007981 10.1289/ehp.10253 PMC2072842

[CR181] Roy JW, Bickerton G (2012) Toxic groundwater contaminants: an overlooked contributor to urban stream syndrome? Environ Sci Technol 46(2):729–736. 10.1021/es2034137 22201254 10.1021/es2034137

[CR182] Roy JW, Bickerton G, Frank RA, Grapentine L, Hewitt LM (2016) Assessing risks of shallow riparian groundwater quality near an oil sands tailings pond. Groundwater 54(4):545–559. 10.1111/gwat.12392 10.1111/gwat.12392 26743232

[CR183] Ryu D, Kim M, Han B, Lee KE, Lee BH, Lee EY, Jung GY, Kim SJ, Park SJ (2020) *Ferrovibrio terrae* sp. nov., isolated from soil. Int J Syst Evol Microbiol 70(2):1042–1047. 10.1099/ijsem.0.003872 31999241 10.1099/ijsem.0.003872

[CR184] Saha P, Chakrabarti T (2006) *Emticicia oligotrophica* gen. nov., sp. nov., a new member of the family ‘Flexibacteraceae’, phylum Bacteroidetes. Int J Syst Evol Microbiol 56(5):991–995. 10.1099/ijs.0.64086-0 16627643 10.1099/ijs.0.64086-0

[CR185] Sawyer AH, & Cardenas MB (2009) Hyporheic flow and residence time distributions in heterogeneous cross-bedded sediment W08406. Water Resour Res 45(8). 10.1029/2008WR007632

[CR186] Schaefer CE, Ho P, Berns E, Werth C (2018) Mechanisms for abiotic dechlorination of trichloroethene by ferrous minerals under oxic and anoxic conditions in natural sediments. Environ Sci Technol 52(23):13747–13755. 10.1021/acs.est.8b04108 30394724 10.1021/acs.est.8b04108

[CR187] Scheutz C, Broholm MM, Durant ND, Weeth EB, Jørgensen TH, Dennis P, Jacobsen CS, Cox EE, Chambon JC, Bjerg PL (2010) Field evaluation of biological enhanced reductive dechlorination of chloroethenes in clayey till. Environ Sci Technol 44(13):5134–5141. 10.1021/es1003044 20527918 10.1021/es1003044

[CR188] Schmidt KR, Gaza S, Voropaev A, Ertl S, Tiehm A (2014) Aerobic biodegradation of trichloroethene without auxiliary substrates. Water Res 59:112–118. 10.1016/j.watres.2014.04.008 24793109 10.1016/j.watres.2014.04.008

[CR189] Schmieder R, Edwards R (2011) Quality control and preprocessing of metagenomic datasets. Bioinformatics 27(6):863–864. 10.1093/bioinformatics/btr026 21278185 10.1093/bioinformatics/btr026 PMC3051327

[CR190] Seddon KR, Stark A, Torres MJ (2000) Influence of chloride, water, and organic solvents on the physical properties of ionic liquids. Pure Appl Chem 72(12):2275–2287. 10.1351/pac200072122275

[CR191] Sekar R, Taillefert M, DiChristina TJ, Löffler FE (2016) Simultaneous transformation of commingled trichloroethylene, tetrachloroethylene, and 1,4- dioxane by a microbially driven fenton reaction in batch liquid cultures. Appl Environ Microbiol 82(21):6335–6343. 10.1128/AEM.02325-16 27542932 10.1128/AEM.02325-16 PMC5066350

[CR192] Severino R, Froufe HJ, Barroso C, Albuquerque L, Lobo-da-Cunha A, da Costa MS, Egas C (2019) High-quality draft genome sequence of *Gaiella occulta* isolated from a 150 meter deep mineral water borehole and comparison with the genome sequences of other deep-branching lineages of the phylum Actinobacteria. Microbiolopen 8(9):e00840. 10.1002/mbo3.840 10.1002/mbo3.840 PMC6741124 30977302

[CR193] Sharma N, Whittaker AC, Watkins SE, Valero L, Vérité J, Puigdefabregas C, Adatte T, Garcés M, Guillocheau M, Castelltort S (2023) Water discharge variations control fluvial stratigraphic architecture in the Middle Eocene Escanilla Formation. Spain. Sci Rep 13(1):6834. 10.1038/s41598-023-33600-6 37100796 10.1038/s41598-023-33600-6 PMC10133228

[CR194] Shaw LJ, Nicol GW, Smith Z, Fear J, Prosser JI, Baggs EM (2006) *Nitrosospira* spp. can produce nitrous oxide via a nitrifier denitrification pathway. Environ Microbiol 8(2):214–222. 10.1111/j.1462-2920.2005.00882.x 16423010 10.1111/j.1462-2920.2005.00882.x

[CR195] Shehu D, Alias Z (2019) Dechlorination of polychlorobiphenyl degradation metabolites by a recombinant glutathione S-transferase from *Acidovorax* sp. KKS102. FEBS Open Bio 9(3):408–419. 10.1002/2211-5463.12405 10.1002/2211-5463.12405 PMC6396153 30868049

[CR196] Şimşir B, Yan J, Im J, Graves D, Löffler FE (2017) Natural attenuation in streambed sediment receiving chlorinated solvents from underlying fracture networks. Environ Sci Technol 51(9):4821–4830. 10.1021/acs.est.6b05554 28328216 10.1021/acs.est.6b05554 PMC6944067

[CR197] Soldatova E, Dong Y, Li J, Liu Y, Zan J, Boeckx P, Sun Z (2021) Nitrogen transformation and pathways in the shallow groundwater–soil system within agricultural landscapes. Environ Geochem Health 43:441–459. 10.1007/s10653-020-00733-w 33000346 10.1007/s10653-020-00733-w

[CR198] Sonne AT, Rasmussen JJ, Höss S, Traunspurger W, Bjerg PL, McKnight US (2018) Linking ecological health to co-occurring organic and inorganic chemical stressors in a groundwater-fed stream system. Sci Total Environ 642:1153–1162. 10.1016/j.scitotenv.2018.06.119 30045497 10.1016/j.scitotenv.2018.06.119

[CR199] Sorokin DY, Vejmelkova D, Lücker S, Streshinskaya GM, Rijpstra WIC, Sinninghe Damsté JS, Kleerbezem R, van Loosdrecht M, Muyzer G, Daims H (2014) *Nitrolancea hollandica* gen. nov., sp. nov., a chemolithoautotrophic nitrite-oxidizing bacterium isolated from a bioreactor belonging to the phylum Chloroflexi. Int J Syst Evol Microbiol 64(Pt_6):1859–1865. 10.1099/ijs.0.062232-0 10.1099/ijs.0.062232-0 24573161

[CR200] Stegen JC, Fredrickson JK, Wilkins MJ, Konopka AE, Nelson WC, Arntzen EV, Chrisler WB, Chu RK, Danczak RE, Fansler SJ, Kennedy DW, Resch CT, Tfaily M (2016) Groundwater–surface water mixing shifts ecological assembly processes and stimulates organic carbon turnover. Nat Commun 7(1):11237. 10.1038/ncomms11237 27052662 10.1038/ncomms11237 PMC4829693

[CR201] Steinbach A, Seifert R, Annweiler E, Michaelis W (2004) Hydrogen and carbon isotope fractionation during anaerobic biodegradation of aromatic hydrocarbons a field study. Environ Sci Technol 38(2):609–616. 10.1021/es034417r 14750739 10.1021/es034417r

[CR202] Storey RG, Fulthorpe RR, Williams DD (1999) Perspectives and predictions on the microbial ecology of the hyporheic zone. Freshw Biol 41(1):119–130. 10.1046/j.1365-2427.1999.00377.x

[CR203] Takahashi Y, Matsumoto A, Morisaki K, Ōmura S (2006) *Patulibacter minatonensis* gen. nov., sp. nov., a novel actinobacterium isolated using an agar medium supplemented with superoxide dismutase, and proposal of Patulibacteraceae fam. nov. Int J Syst Evol Microbiol 56(2):401–406. 10.1099/ijs.0.63796-0 16449447 10.1099/ijs.0.63796-0

[CR204] Takeda M, Yoneya A, Miyazaki Y, Kondo K, Makita H, Kondoh M, Suzuki I, Koizumi JI (2008) *Prosthecobacter fluviatilis* sp. nov., which lacks the bacterial tubulin btubA and btubB genes. Int J Syst Evol Microbiol 58(7):1561–1565. 10.1099/ijs.0.65787-0 18599695 10.1099/ijs.0.65787-0

[CR205] Taylor AE, Bottomley PJ (2006) Nitrite production by *Nitrosomonas europaea* and *Nitrosospira* sp. AV in soils at different solution concentrations of ammonium. Soil Biol Biochem 38(4):828–836. 10.1016/j.soilbio.2005.08.001

[CR206] Taylor AE, Dolan ME, Bottomley PJ, Semprini L (2007) Utilization of fluoroethene as a surrogate for aerobic vinyl chloride transformation. Environ Sci Technol 41:6378–6383. 10.1021/es0701255 17948782 10.1021/es0701255

[CR207] Tesoriero AJ, Gronberg JA, Juckem PF, Miller MP, Austin BP (2017) Predicting redox-sensitive contaminant concentrations in groundwater using random forest classification. Water Resour Res 53(8):7316–7331. 10.1002/2016WR020197

[CR208] Tesoriero AJ, Stratton LE, Miller MP (2021) Influence of redox gradients on nitrate transport from the landscape to groundwater and streams. Sci Total Environ 800:150200. 10.1016/j.scitotenv.2021.15020 34625279 10.1016/j.scitotenv.2021.150200

[CR209] Tobiszewski M, Namieśnik J (2012) Abiotic degradation of chlorinated ethanes and ethenes in water. Environ Sci Pollut Res 19:1994–2006. 10.1007/s11356-012-0764-9 10.1007/s11356-012-0764-9 PMC3390699 22293908

[CR210] Toumi M, Abbaszade G, Sbaoui Y, Farkas R, Ács É, Jurecska L, Tóth E (2021) Cultivation and molecular studies to reveal the microbial communities of groundwaters discharge located in Hungary. Water 13(11):1533. 10.3390/w13111533

[CR211] Vallaeys T, Albino L, Soulas G, Wright AD, Weightman AJ (1998) Isolation and characterization of a stable 2, 4-dichlorophenoxyacetic acid degrading bacterium, *Variovorax paradoxus*, using chemostat culture. Biotechnol Lett 20:1073–1076. 10.1023/A:1005438930870

[CR212] Vogel M, Nijenhuis I, Lloyd J, Boothman C, Pöritz M, Mackenzie K (2018) Combined chemical and microbiological degradation of tetrachloroethene during the application of Carbo-Iron at a contaminated field site. Sci Total Environ 628:1027–1036. 10.1016/j.scitotenv.2018.01.310 30045527 10.1016/j.scitotenv.2018.01.310

[CR213] Vogel TM, McCarty PL (1985) Biotransformation of tetrachloroethylene to trichloroethylene, dichloroethylene, vinyl chloride, and carbon dioxide under methanogenic conditions. Appl Environ Microbiol 49(5):1080–1083. 10.1128/aem.49.5.1080-1083.1985 3923927 10.1128/aem.49.5.1080-1083.1985 PMC238509

[CR214] Wagner AO, Markt R, Mutschlechner M, Lackner N, Prem EM, Praeg N, Illmer P (2019) Medium preparation for the cultivation of microorganisms under strictly anaerobic/anoxic conditions. JoVE (Journal of Visualized Experiments) 150:e60155. 10.3791/60155 10.3791/60155 PMC6796894 31475968

[CR215] Wang P, Zhang Y, Jin J, Wang T, Wang J, Jiang B (2020) A high-efficiency phenanthrene-degrading *Diaphorobacter* sp. isolated from PAH-contaminated river sediment. Sci Total Environ 746:140455. 10.1016/j.scitotenv.2020.140455 32758981 10.1016/j.scitotenv.2020.140455

[CR216] Watanabe K, Komatsu N, Ishii Y, Negishi M (2009) Effective isolation of bacterioplankton genus *Polynucleobacter* from freshwater environments grown on photochemically degraded dissolved organic matter. FEMS Microbiol Ecol 67(1):57–68. 10.1111/j.1574-6941.2008.00606.x 19049496 10.1111/j.1574-6941.2008.00606.x

[CR217] Weatherill JJ, Atashgahi S, Schneidewind U, Krause S, Ullah S, Cassidy N, Rivett MO (2018) Natural attenuation of chlorinated ethenes in hyporheic zones: a review of key biogeochemical processes and in-situ transformation potential. Water Res 128:362–382. 10.1016/j.watres.2017.10.059 29126033 10.1016/j.watres.2017.10.059

[CR218] Weatherill JJ, Krause S, Ullah S, Cassidy NJ, Levy A, Drijfhout FP, Rivett MO (2019) Revealing chlorinated ethene transformation hotspots in a nitrate-impacted hyporheic zone. Water Res 161:222–231. 10.1016/j.watres.2019.05.083 31200219 10.1016/j.watres.2019.05.083

[CR219] Weatherill J, Krause S, Voyce K, Drijfhout F, Levy A, Cassidy N (2014) Nested monitoring approaches to delineate groundwater trichloroethene discharge to a UK lowland stream at multiple spatial scales. J Contam Hydrol 158:38–54. 10.1016/j.jconhyd.2013.12.001 24424265 10.1016/j.jconhyd.2013.12.001

[CR220] Wehncke EV, & Mariano NA (2021) Groundwater and its role in maintaining the ecological functions of ecosystems—a review. Intensified Land and Water Use 55–86. 10.1007/978-3-030-65443-6_4

[CR221] Wei N, Finneran KT (2011) Influence of ferric iron on complete dechlorination of trichloroethylene (TCE) to ethene: Fe (III) reduction does not always inhibit complete dechlorination. Environ Sci Technol 45(17):7422–7430. 10.1021/es201501a 21777002 10.1021/es201501a

[CR222] Whittleston RA, Stewart DI, Mortimer RJ, Burke IT (2013) Enhancing microbial iron reduction in hyperalkaline, chromium contaminated sediments by pH amendment. Appl Geochem 28:135–144. 10.1016/j.apgeochem.2012.10.003

[CR223] Wilhelm RC (2018) Following the terrestrial tracks of *Caulobacter*-redefining the ecology of a reputed aquatic oligotroph. ISME J 12(12):3025–3037. 10.1038/s41396-018-0257-z 30108303 10.1038/s41396-018-0257-z PMC6246563

[CR224] Willems A, Goor M, Thielemans S, Gillis M, Kersters K, De Ley J (1992) Transfer of several phytopathogenic *Pseudomonas* species to *Acidovorax* as *Acidovorax avenae* subsp *avenae* subsp. nov., comb nov., *Acidovorax avenae* subsp *citrulli*, *Acidovorax avenae* subsp *cattleyae*, and *Acidovorax konjaci*. Int J Syst Evol Microbiol 42(1):107–119. 10.1099/00207713-42-1-107 10.1099/00207713-42-1-107 1371056

[CR225] Willis JR, González-Torres P, Pittis AA, Bejarano LA, Cozzuto L, Andreu-Somavilla N, Alloza-Trabado M, Valentín A, Ksiezopolska E, Company C, Onywera H, Montfort M, Hermoso A, Iraola-Guzmán S, Saus E, Labeeuw A, Carolis C, Hecht J, Ponomarenko J, Gabaldón T (2018) Citizen science charts two major “stomatotypes” in the oral microbiome of adolescents and reveals links with habits and drinking water composition. Microbiome 6:1–17. 10.1186/s40168-018-0592-3 30522523 10.1186/s40168-018-0592-3 PMC6284318

[CR226] Wilson FP, Liu X, Mattes TE, Cupples AM (2016) *Nocardioides*, *Sediminibacterium*, *Aquabacterium*, *Variovorax*, and *Pseudomonas* linked to carbon uptake during aerobic vinyl chloride biodegradation. Environ Sci Pollut Res 23:19062–19070. 10.1007/s11356-016-7099-x 10.1007/s11356-016-7099-x 27343076

[CR227] Wittlingerová Z, Macháčková J, Petruželková A, Zimová M (2016) Occurrence of perchloroethylene in surface water and fish in a river ecosystem affected by groundwater contamination. Environ Sci Pollut Res 23:5676–5692. 10.1007/s11356-015-5806-7 10.1007/s11356-015-5806-7 26578381

[CR228] Woessner WW (2000) Stream and fluvial plain ground water interactions: rescaling hydrogeologic thought. Groundwater 38(3):423–429. 10.1111/j.1745-6584.2000.tb00228.x

[CR229] Wu YT, Chiang PW, Tandon K, Rogozin DY, Degermendzhy AG, Tang SL (2021) Single-cell genomics-based analysis reveals a vital ecological role of *Thiocapsa* sp. LSW in the meromictic Lake Shunet, Siberia. Microb Genom. 10.1099/mgen.0.000712 10.1099/mgen.0.000712 PMC8767323 34860152

[CR230] Wu M, Wu J, Wu J (2017) Simulation of DNAPAPL migration in heterogeneous translucent porous media based on estimation of representative elementary volume. J Hydrol 553:276–288. 10.1016/j.jhydrol.2017.08.005

[CR231] Xie B, Liu B, Yi Y, Yang L, Liang D, Zhu Y, Liu H (2016) Microbiological mechanism of the improved nitrogen and phosphorus removal by embedding microbial fuel cell in anaerobic–anoxic–oxic wastewater treatment process. Bioresource Technol 207:109–117. 10.1016/j.biortech.2016.01.090 10.1016/j.biortech.2016.01.090 26874439

[CR232] Xu H, Chang J, Wang H, Liu Y, Zhang X, Liang P, Huang X (2019) Enhancing direct interspecies electron transfer in syntrophic-methanogenic associations with (semi) conductive iron oxides: effects and mechanisms. Sci Total Environ 695:133876. 10.1016/j.scitotenv.2019.133876 31756846 10.1016/j.scitotenv.2019.133876

[CR233] Xu L, Su J, Ali A, Chang Q, Shi J, Yang Y (2022) Denitrification performance of nitrate–dependent ferrous (Fe^2+^) oxidizing *Aquabacterium* sp. XL4: adsorption mechanisms of bio–precipitation of phenol and estradiol. J Hazard Mater 427(127918):127918. 10.1016/j.jhazmat.2021.127918 34863560 10.1016/j.jhazmat.2021.127918

[CR234] Xue X, Wang D, Yi X, Li Y, Han H (2021) Simultaneously autotrophic denitrification and organics degradation in low-strength coal gasification wastewater (LSCGW) treatment via microelectrolysis-triggered Fe (II)/Fe (III) cycle. Chemosphere 278:130460. 10.1016/j.chemosphere.2021.130460 33838412 10.1016/j.chemosphere.2021.130460

[CR235] Yan A, Guo X, Hu D, Chen X (2022) Reactive transport of NH4+ in the hyporheic zone from the ground water to the surface water. Water 14(8):1237. 10.3390/w14081237

[CR236] Yan N, An M, Chu J, Cao L, Zhu G, Wu W, Wang W, Zhang Y, Rittmann BE (2021) More rapid dechlorination of 2, 4-dichlorophenol using acclimated bacteria. Bioresour Technol 326:124738. 10.1016/j.biortech.2021.124738 33497925 10.1016/j.biortech.2021.124738

[CR237] Yoon J, Matsuo Y, Adachi K, Nozawa M, Matsuda S, Kasai H, Yokota A (2008) Description of *Persicirhabdus sediminis* gen. nov., sp. nov., *Roseibacillus ishigakijimensis* gen. nov., sp. nov., *Roseibacillus ponti* sp. nov., *Roseibacillus persicicus* sp. nov., *Luteolibacter pohnpeiensis* gen. nov., sp. nov. and *Luteolibacter algae* sp. nov., six marine members of the phylum ‘Verrucomicrobia’, and emended descriptions of the class Verrucomicrobiae, the order Verrucomicrobiales and the family Verrucomicrobiaceae. Int J Syst Evol Microbiol 58(4):998–1007. 10.1099/ijs.0.65520-0 10.1099/ijs.0.65520-0 18398209

[CR238] You X, Liu S, Berns-Herrboldt EC, Dai C, Werth CJ (2023) Kinetics of hydroxyl radical production from oxygenation of reduced iron minerals and their reactivity with trichloroethene: effects of iron amounts, iron species, and sulfate reducing bacteria. Environ Sci Technol 57(12):4892–4904. 10.1021/acs.est.3c00122 36921080 10.1021/acs.est.3c00122

[CR239] Young JM, Skvortsov T, Arkhipova K, Allen CC (2018) Draft genome sequence of the predatory marine bacterium *Halobacteriovorax* sp strain JY17. Genome Announc 6(1):10–1128. 10.1128/genomeA.01416-17 10.1128/genomeA.01416-17 PMC5754496 29301887

[CR240] Zalesak M, Ruzicka J, Vicha R, Dvorackova M (2017) Cometabolic degradation of dichloroethenes by *Comamonas testosteroni* RF2. Chemosphere 186:919–927. 10.1016/j.chemosphere.2017.07.156 28830064 10.1016/j.chemosphere.2017.07.156

[CR241] Zanotti C, Rotiroti M, Fumagalli L, Stefania GA, Canonaco F, Stefenelli G, Prévôt ASH, Leoni B, Bonomi T (2019) Groundwater and surface water quality characterization through positive matrix factorization combined with GIS approach. Water Res 159:122–134. 10.1016/j.watres.2019.04.058 31082643 10.1016/j.watres.2019.04.058

[CR242] Zeng Y, Kasalický V, Šimek K, Koblížek M (2012) Genome sequences of two freshwater betaproteobacterial isolates, *Limnohabitans* species strains Rim28 and Rim47, indicate their capabilities as both photoautotrophs and ammonia oxidizers. J Bacteriol. 10.1128/jb.01481-12 10.1128/JB.01481-12 PMC3486343 23105051

[CR243] Zeng Y, Selyanin V, Lukeš M, Dean J, Kaftan D, Feng F, Koblížek M (2015) Characterization of the microaerophilic, bacteriochlorophyll a-containing bacterium *Gemmatimonas phototrophica* sp. nov., and emended descriptions of the genus *Gemmatimonas* and *Gemmatimonas aurantiaca*. Int J Syst Evol Microbiol 65(Pt_8):2410–2419. 10.1099/ijs.0.000272 10.1099/ijs.0.000272 25899503

[CR244] Zeppilli M, Dell’Armi E, Papini MP, Majone M (2021) Sequential reductive/oxidative bioelectrochemical process for groundwater perchloroethylene removal. Chem Eng Trans 86:373–378. 10.3303/CET2186063

[CR245] Zhang H, Sekiguchi Y, Hanada S, Hugenholtz P, Kim H, Kamagata Y, Nakamura K (2003) *Gemmatimonas aurantiaca* gen. nov., sp. nov., a gram-negative, aerobic, polyphosphate-accumulating micro-organism, the first cultured representative of the new bacterial phylum Gemmatimonadetes phyl. nov. Int J Syst Evol Microbiol 53(4):1155–1163. 10.1099/ijs.0.02520-0 10.1099/ijs.0.02520-0 12892144

[CR246] Zheng F, Gao Y, Sun Y, Shi X, Xu H, Wu J (2015) Influence of flow velocity and spatial heterogeneity on DNAPL migration in porous media: insights from laboratory experiments and numerical modelling. Hydrogeol J 23(8):1703. 10.1007/s10040-015-1314-6

[CR247] Zhu A, Yang Z, Liang Z, Gao L, Li R, Hou L, Li S, Xie Z, Wu Y, Chen J, Cao L (2020) Integrating hydrochemical and biological approaches to investigate the surface water and groundwater interactions in the hyporheic zone of the Liuxi River basin, southern China. J Hydrol 583:124622. 10.1016/j.jhydrol.2020.124622

[CR248] Zuo R, Xue Z, Wang J, Meng L, Zhao X, Pan M, Cai W (2022) Spatiotemporal variations of redox conditions and microbial responses in a typical river bank filtration system with high Fe2+ and Mn2+ contents. J Hydrol 609:127777. 10.1016/j.jhydrol.2022.127777

