# Supplementary material for: Control of alluvial aquifer architecture on reductive and oxidative dechlorination of chloroethenes
Source: Environ Sci Pollut Res Int. 2026 Jun 20;33(20):9943–77. doi: 10.1007/s11356-026-37894-7 (PMC13350131; doi:10.1007/s11356-026-37894-7)
Supplement: Supplementary file 1 — (DOCX 2.55 MB) [file 11356_2026_37894_MOESM1_ESM.pdf]

Supporting Information

(SI document)

Effect of geological structure on natural attenuation of chloroethenes in the aquifer-hyporheic zone system

Puigserver D.[a], Herrero J.[b], Cortés A.[c], Millán A.[d], Parker B.[e] and Carmona JM.[f]

[a, b, d, f] Department of Mineralogy, Petrology and Applied Geology. Faculty of Earth Sciences. University of Barcelona (UB). Water Research Institute (IdRA-UB). C/ Martí i Franquès, s/n. E-08028 Barcelona (Spain).

[c] Department of Biology, Healthcare and the Environment. Faculty of Pharmacy and Food Sciences. University of Barcelona (UB). C/ Joan XXIII, 27-31. E-08028 Barcelona (Spain). 08028 Barcelona

[e] School of Engineering, University of Guelph, 50, Stone Road East, Guelph, N1G 2W1, Ontario, Canada

[a*] Serra Húnter Tenure-elegible Lecturer. puigserverdiana@ub.edu

[b,] jofreherreroferran@ub.edu

[c] acortes@ub.edu

[d] amillan@ub.edu

[e] bparker@uoguelph.ca

[f] jmcarmona@ub.edu

* Corresponding author

INDEX

Table SI – 1 3
Table SI – 2 5
Figure SI – 1 6
Figure SI – 2 8
Figure SI – 3 9
Figure SI – 4 11
Figure SI – 5 12
Figure SI – 6 13
Figure SI – 7 14

Table SI – 1

Table SI–1. Values of foc (as a fraction of one) at different depths in boreholes S1, S2 and S9 (interchannel areas) and in boreholes S3, S5, S10, S6, and S11 along a flow line in paleochannel areas. See map of Figure 2A in the main document.

Interchannel areas. foc average values below the ground surface (depth of water table 0.8 m close to the influent right bank)					
S1 depth (m)	foc (as a fraction of one)	S2 depth (m)	foc (as a fraction of one)	S9 depth (m)	foc (as a fraction of one)
0	0.0560	0	0.0399	0	0.0480
0.09	0.0570	0.09	0.0340	-0.21	0.0455
0.17	0.0446	0.35	0.0255	0.35	0.0351
0.3	0.0231	0.66	0.0119	0.66	0.0175
0.68	0.0214	0.87	0.0148	0.87	0.0181
0.88	0.0090	1.2	0.0091	1.2	0.0090
1.2	0.0082	1.65	0.0105	1.65	0.0093
1.8	0.0045	1.9	0.0115	1.9	0.0080
2.2	0.0056	2	0.0043	2	0.0050
2.5	0.0045	2.7	0.0123	2.7	0.0084
		2.8	0.0064	2.9	0.0032
		3.2	0.0018	3.1	0.0070
Average value	0.0064	Average value	0.0088	Average value	0.0085

Paleochannel areas. foc average values below the ground surface (depth of water table 0.8 m close to the influent right bank)									
S3 depth (m)	foc (as a fraction of one)	S5 depth (m)	foc (as a fraction of one)	S10 depth (m)	foc (as a fraction of one)	S6 depth (m)	foc (as a fraction of one)	S11 depth (m)	foc (as a fraction of one)
0	0.1280	0	0.0528	0	0.0260	0	0.0252	0	0.0580
0.08	0.0352	0.1	0.0444	0.09	0.0246	0.1	0.0293	0.1	0.0334
0.35	0.0269	0.6	0.0794	0.475	0.0339	0.4	0.0222	0.5	0.0406
0.6	0.0068	0.7	0.0089	0.65	0.0048	0.8	0.0054	0.75	0.0065
1.2	0.0079	1.1	0.0049	1.15	0.0036	1.35	0.0058	1.225	0.0055
1.5	0.0049	1.2	0.0085	1.35	0.0044	1.7	0.0048	1.45	0.0056
1.8	0.0039	1.5	0.0068	1.65	0.0038	1.8	0.0045	1.65	0.0048
2.1	0.0036	1.85	0.0103	1.975	0.0046	2.1	0.0034	1.975	0.0055
2.5	0.0033	2.3	0.0031	2.4	0.0020	2.4	0.0028	2.35	0.0028
		2.4	0.0010	2.7	0.0016	2.9	0.0039	2.65	0.0022
		3.1	0.0032	3	0.0025	3	0.0045	3.05	0.0034
Average value	0.0047	Average value	0.0054	Average value	0.0032	Average value	0.0044	Average value	0.0043

Table SI – 2

Table SI–2. Hydraulic conductivity values determined by field slug tests in the monitoring network of the Catalan Water Agency along the X-X' profile and transects following the paleochannel and interchannel areas.

**Profile X-X'**	**Paleochannel**
**Piezometers**	**K (m/d)**
SD29	310
SD28	85
SD30	295
SD31	315
SD32	300

**Transect E-E'**	**Paleochannel**
**Piezometers**	**K (m/d)**
SD30	295
SD55	180
SD17	130

**Transect B-B'**	**Paleochannel**
**Piezometers**	**K (m/d)**
SD29	310
SD19	65

**Transect F-F'**	**Paleochannel**
**Piezometers**	**K (m/d)**
SD31	315
SD57	180
SD17	130

**Transect C-C'**	**Paleochannel**
**Piezometers**	**K (m/d)**
SD51	285
SD54	235
SD55	180
SD17	130

**Transect G-G'**	**Paleochannel**
**Piezometers**	**K (m/d)**
SD32	300
SD59	245
SD58	205
SD17	130

**Transect D-D'**	**Interchannel**
**Piezometers**	**K (m/d)**
SD28	85
SD18	30

Figure SI – 1

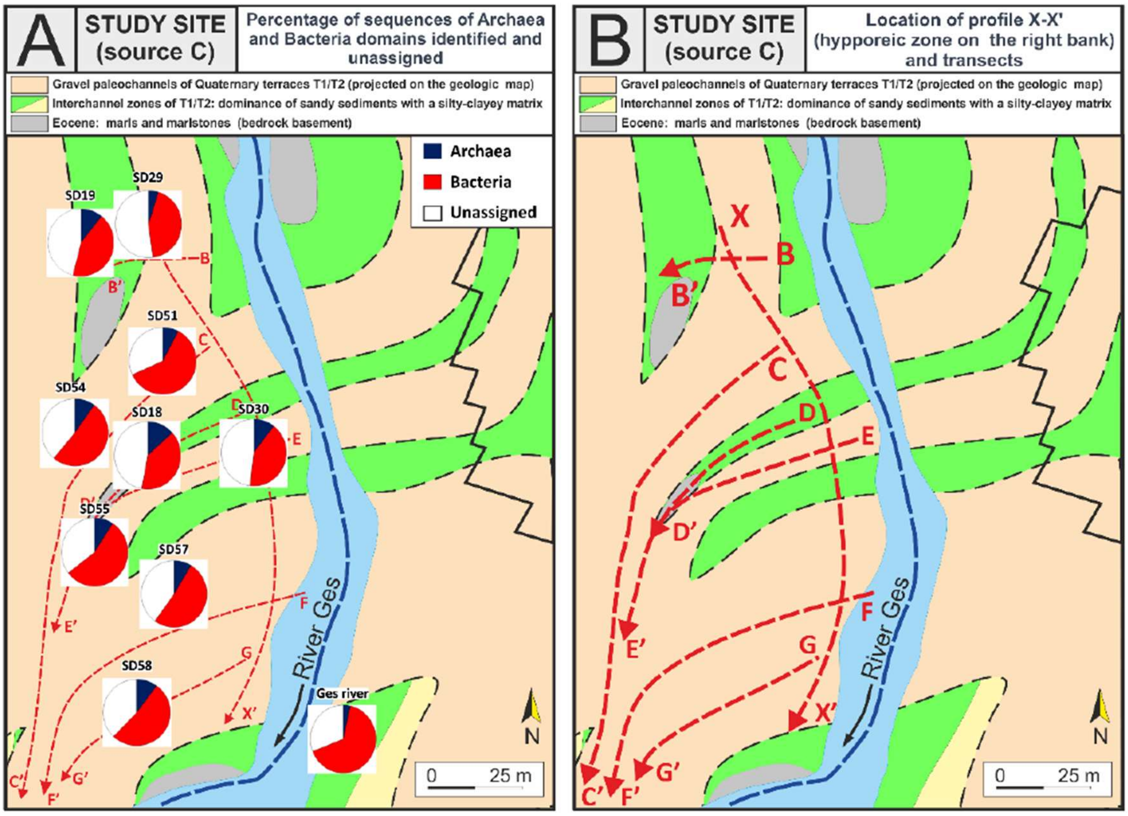

Figure SI – 1. Pie charts of the Archaea and Bacteria domains along the groundwater flow in the study site. Each segment of the pie chart represents the percentage of microbial sequences (relative to the total identified and the unassigned sequences in the sampled groundwater) attributed to the Archaea and Bacteria domains. Spring 2022 sampling survey.

Processes involving the different phyla identified at the study site

Protobacteria exhibit a wide diversity, encompassing both anaerobic and microaerophilic as well as facultative aerobic forms. Within this phylum lie genera responsible for processes such as Fe and Mn reduction, as well as sulfate reduction.

The phylum Actinobacteria is comprised of Gram-positive Eubacteria, many of which are ubiquitous in soil and play a fundamental role in organic matter decomposition. These microorganisms are typically facultative aerobes capable of fermenting a broad range of organic compounds, some of which are associated with manure disposal.

The phylum Bacteroidetes is characterized by its fermentative metabolism and has been identified in cultures enriched with chloroethenes, as well as in contaminated areas, although no direct relationship with dehalogenation processes has been established.

Within the Archaeal domain, three abundant phyla have been detected: Crenarchaeota, Euryarchaeota, and Thaumarchaeota.

The phylum Euryarchaeota is associated with methanogenesis, particularly through members of the Methanomassiliicoccales order, which is consistent with the reducing conditions observed in piezometers SD19, SD18, and SD30, associated with a higher presence of natural or anthropogenic organic matter.

The phylum Crenarchaeota is related to sulfate reduction, especially through members of the Desulfurococcales order, primarily in smaller zones such as around piezometers SD29, SD19, SD30, and SD18.

The genus *Nitrospirae* is associated with aerobic bacteria performing nitrification, either through heterotrophic oxidation (ammonium and/or nitrite) or autotrophic oxidation (urea).

The phylum Thaumarchaeota is associated with aerobic bacteria performing nitrification, especially through the Nitrosopumilaceae and Nitrososphaeraceae orders.

The phylum Firmicutes comprises a group of Gram-positive bacteria, most of which possess a fermentative metabolism. Many members of this phylum produce endospores, conferring resistance to desiccation and enabling survival in extreme conditions.

Figure SI – 2

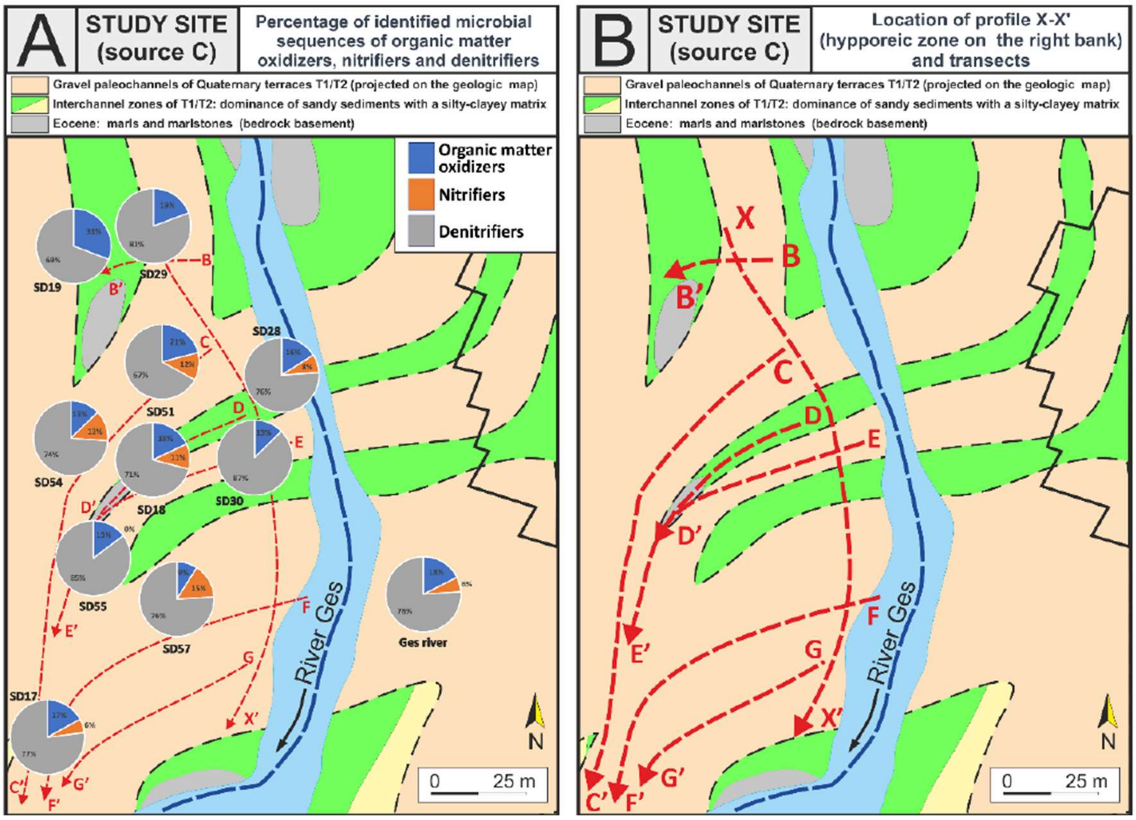

Figure SI–2. Pie charts of microorganisms capable of organic matter oxidation, nitrification, and denitrification along the groundwater flow. Each segment of the pie chart represents the percentage of microbial sequences (relative to the total identified in the sampled groundwater) attributed to microorganisms of the organic matter oxidizing, nitrifying, and denitrifying genera. Spring 2022 sampling survey.

Figure SI – 3

a)

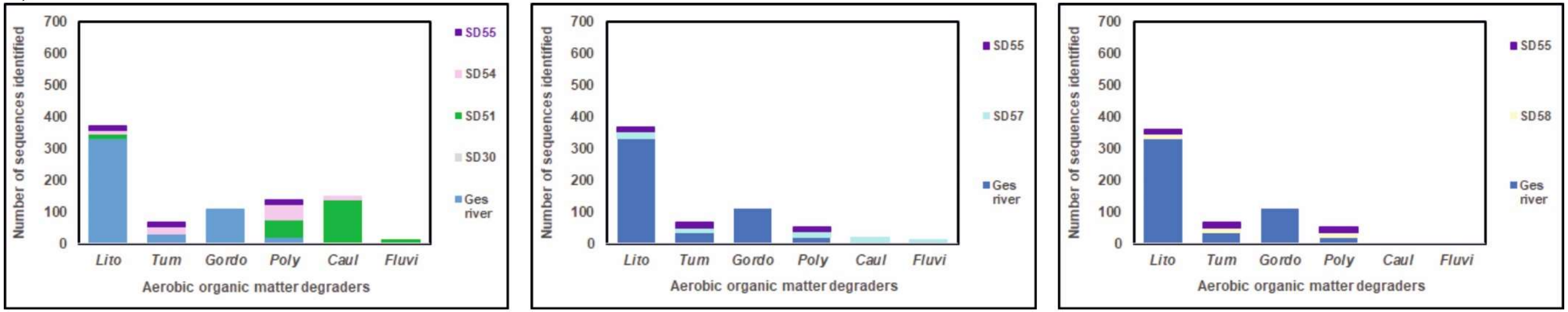

Lito	Turn	Gordo	Poly	Caul	Fluvi
Litorilinea	Turneriella	Gordonia	Polynucleobacter	Caulobacter	Fluviicola

b)

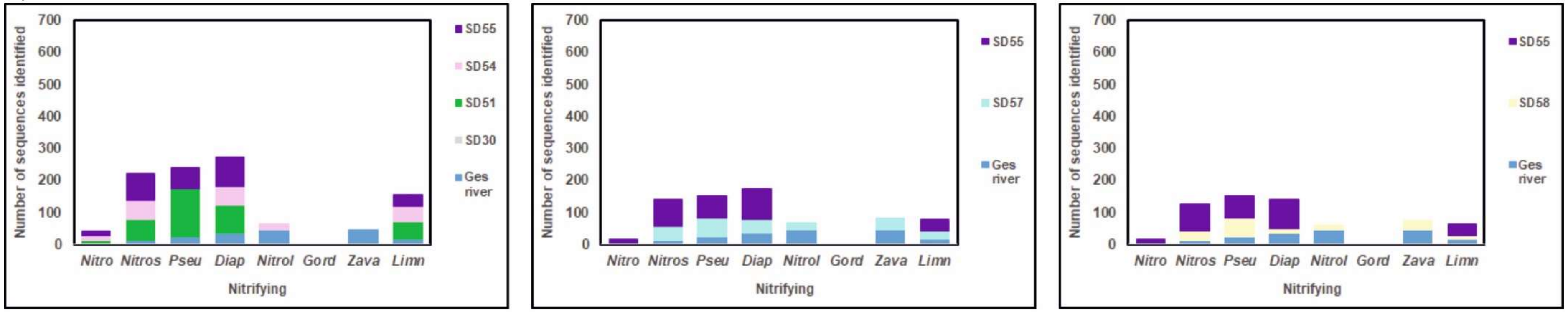

Nitro	Nitros	Pseu	Diap	Nitrol	Gord	Zava	Limn
Nitrosomonas	Nitrosospira	Pseudomonas	Diaphorobacter	Nitrolancea	Gordonia	Zavarzinella	Limnohabitans

c)

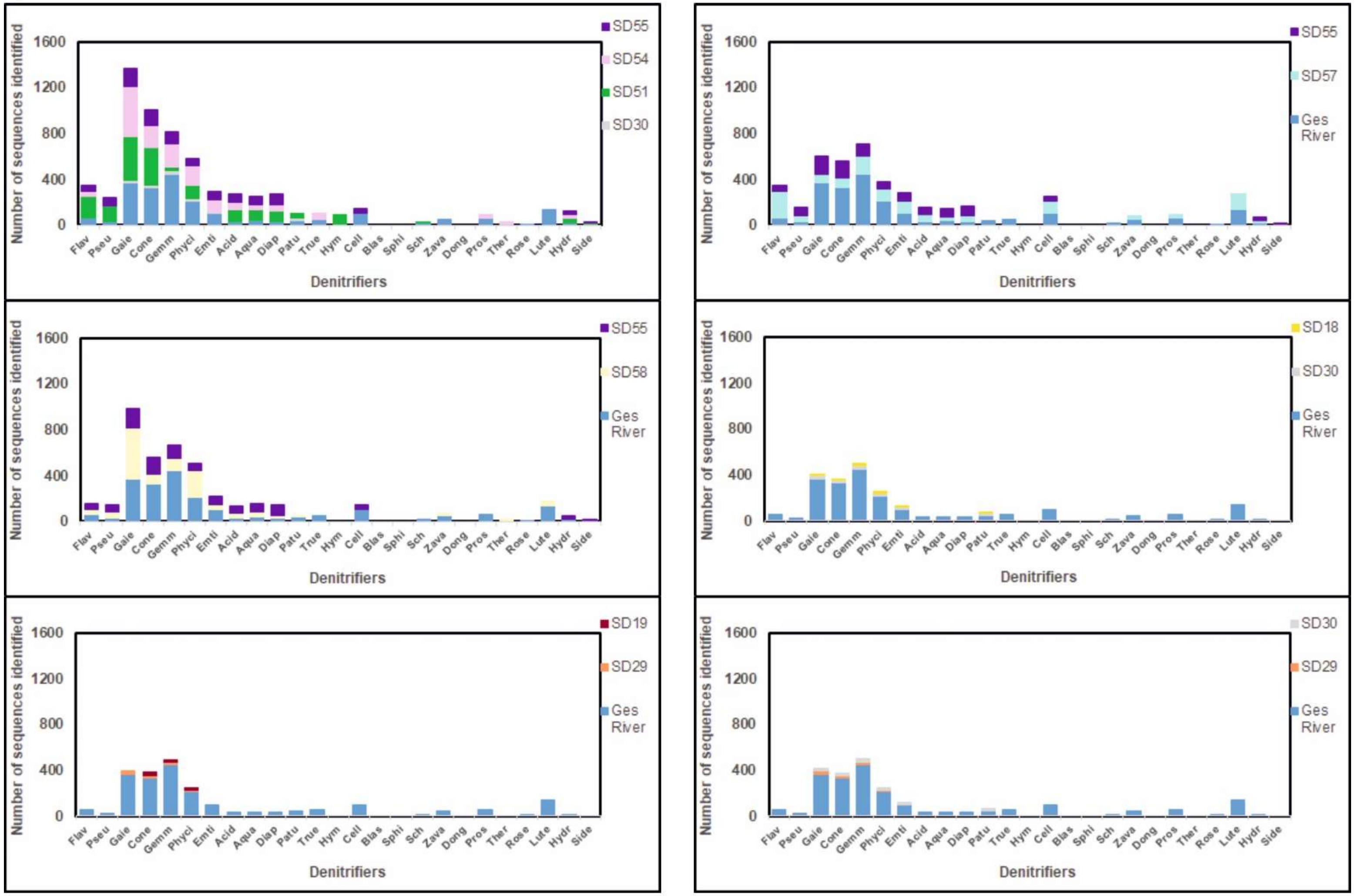

Flav	*Pseu*	*Gaie*	*Cone*	*Gemm*	*Phyci*	*Emti*	*Acid*	*Aqua*	*Diap*	*Patu*	*True*	*Hym*
Flavobacterium	*Pseudomonas*	*Gaiella*	*Conexibacter*	*Gemmatimonas*	*Phycisphaera*	*Emticicia*	*Acidovorax*	*Aquabacterium*	*Diaphorobacter*	*Patulibacter*	*Truepera*	*Hymenobacter*
Cell	*Blas*	*Sphi*	*Sch*	*Zava*	*Dong*	*Pros*	*Ther*	*Rose*	*Lute*	*Hydr*	*Side*	
Cellvibrio	*Blastococcus*	*Sphingobacterium*	*Schlesneria*	*Zavarzinella*	*Dongia*	*Prosthecobacter*	*Thermogutta*	*Roseomonas*	*Luteolibacter*	*Hydrogenophaga*	*Sideroxydans*	

Figure SI–3. Identified genus capable organic matter oxidizing (a), nitrifying (b) and denitrifying (c) in the study site. Each color within the bars represents a piezometer within the monitoring network of the Catalan Water Agency.. Spring 2022 sampling survey.

Figure SI – 4

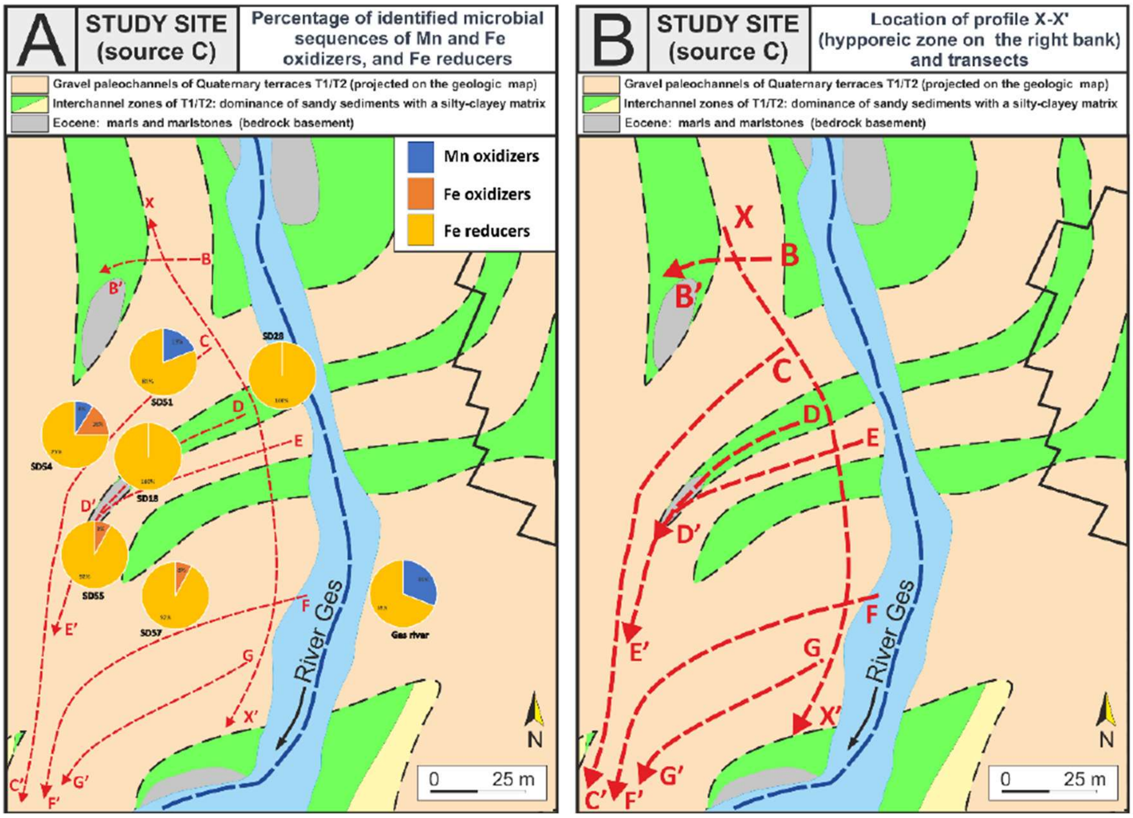

Figure SI–4. Pie charts of microorganisms capable of Mn-oxidizing, Fe-oxidizing, and Fe-reducing along the groundwater flow. Each segment of the pie chart represents the percentage of microbial sequences (relative to the total identified in the sampled groundwater) attributed to the mentioned biogeochemical processes. Spring 2022 sampling survey.

Figure SI – 5

a)

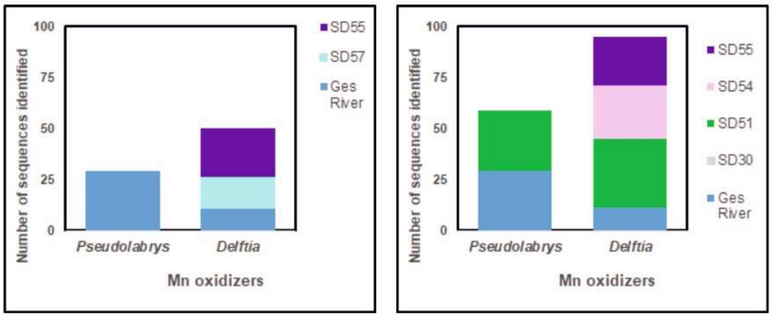

b)

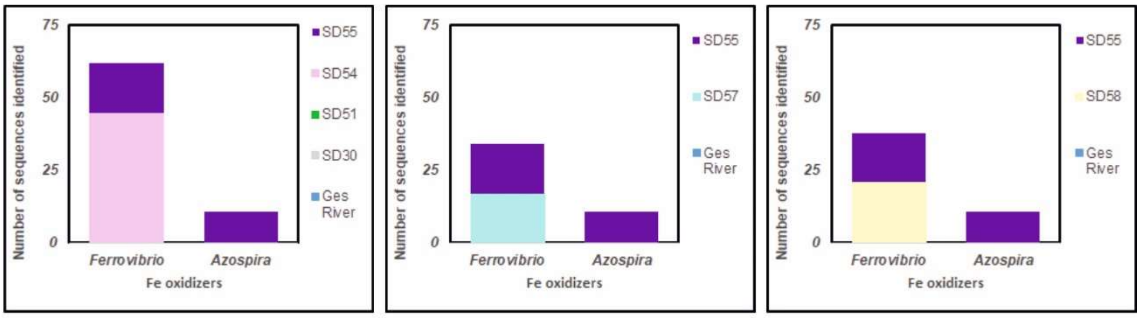

c)

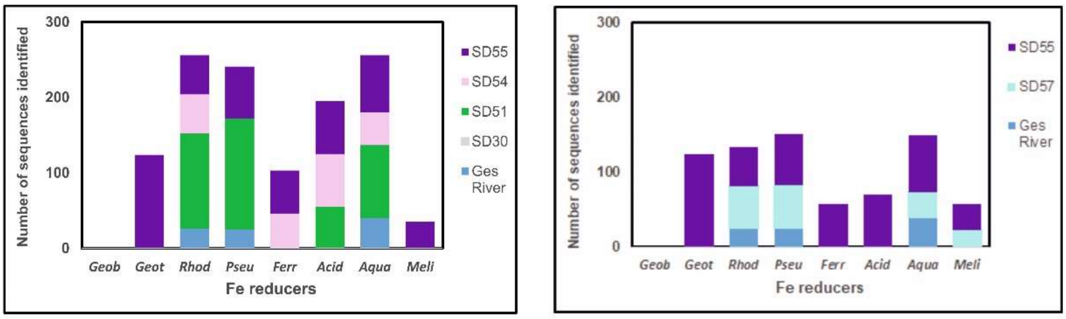

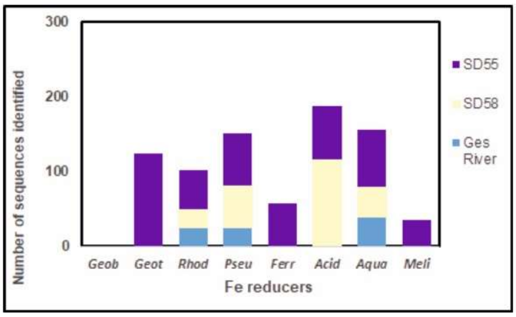

Geob	*Geot*	*Rhod*	*Pseu*
Geobacter	*Geothrix*	*Rhodoferax*	*Pseudomonas*
Ferr	*Acid*	*Aqua*	*Meli*
Ferribacterium	*Aciditerrimonas*	*Aquabacterium*	*Melioribacter*

Figure SI–5. Identified genus capable of Mn-oxidizing (a), Fe-oxidizing (b) and Fe-reducing (c) in the study site. Each color within the bars represents a piezometer within the monitoring network of the Catalan Water Agency.. Spring 2022 sampling survey.

Figure SI – 6

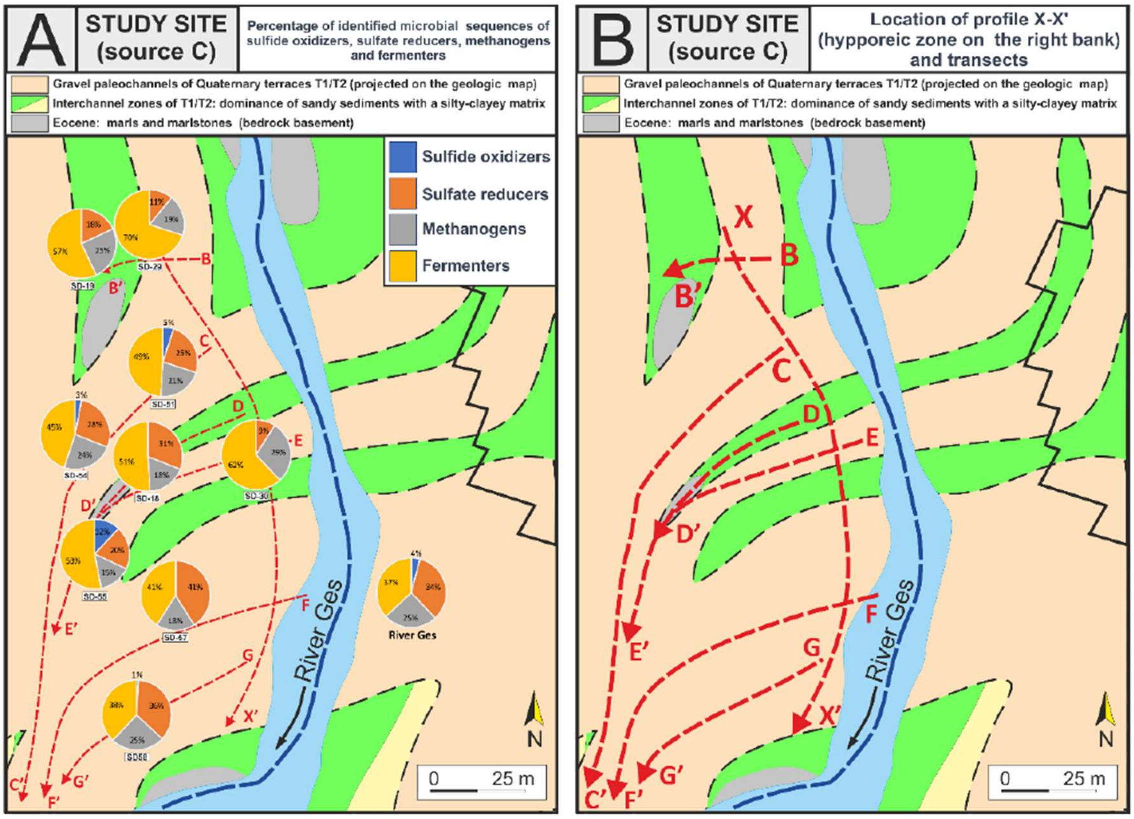

Figure SI–6. Pie charts of microorganisms capable of sulfide oxidation, sulfate reduction, methanogenesis, and fermentation along the groundwater flow. Each segment of the pie chart represents the percentage of microbial sequences (relative to the total identified in the sampled groundwater) attributed to microorganisms of the sulfide oxidation, sulfate reduction, methanogenesis, and fermentation genera.. Spring 2022 sampling survey.

Figure SI – 7

a)

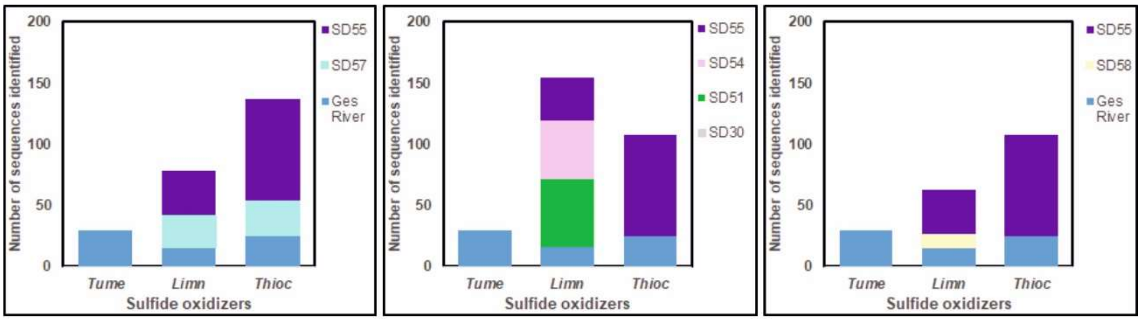

Tume	*Limn*	*Thioc*
Tumebacillus	*Limnohabitans*	*Thiocapsa*

b)

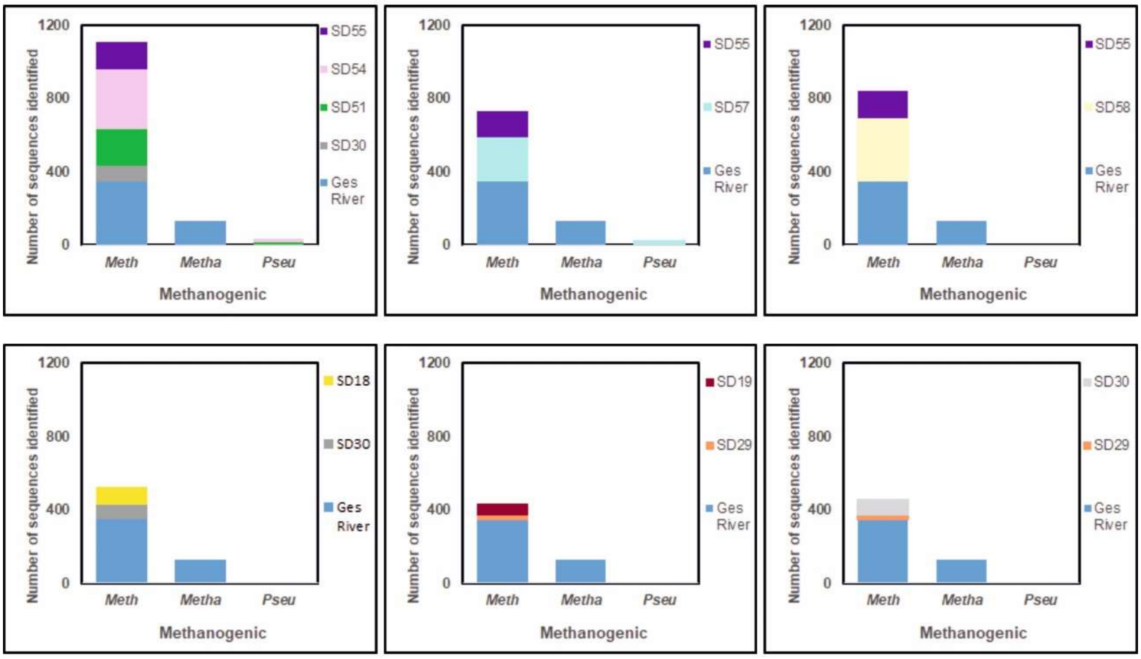

Meth	*Metha*	*Pseud*
Methanomassiliicoccus	*Methanospirillum*	*Pseudorhodobacter*

c)

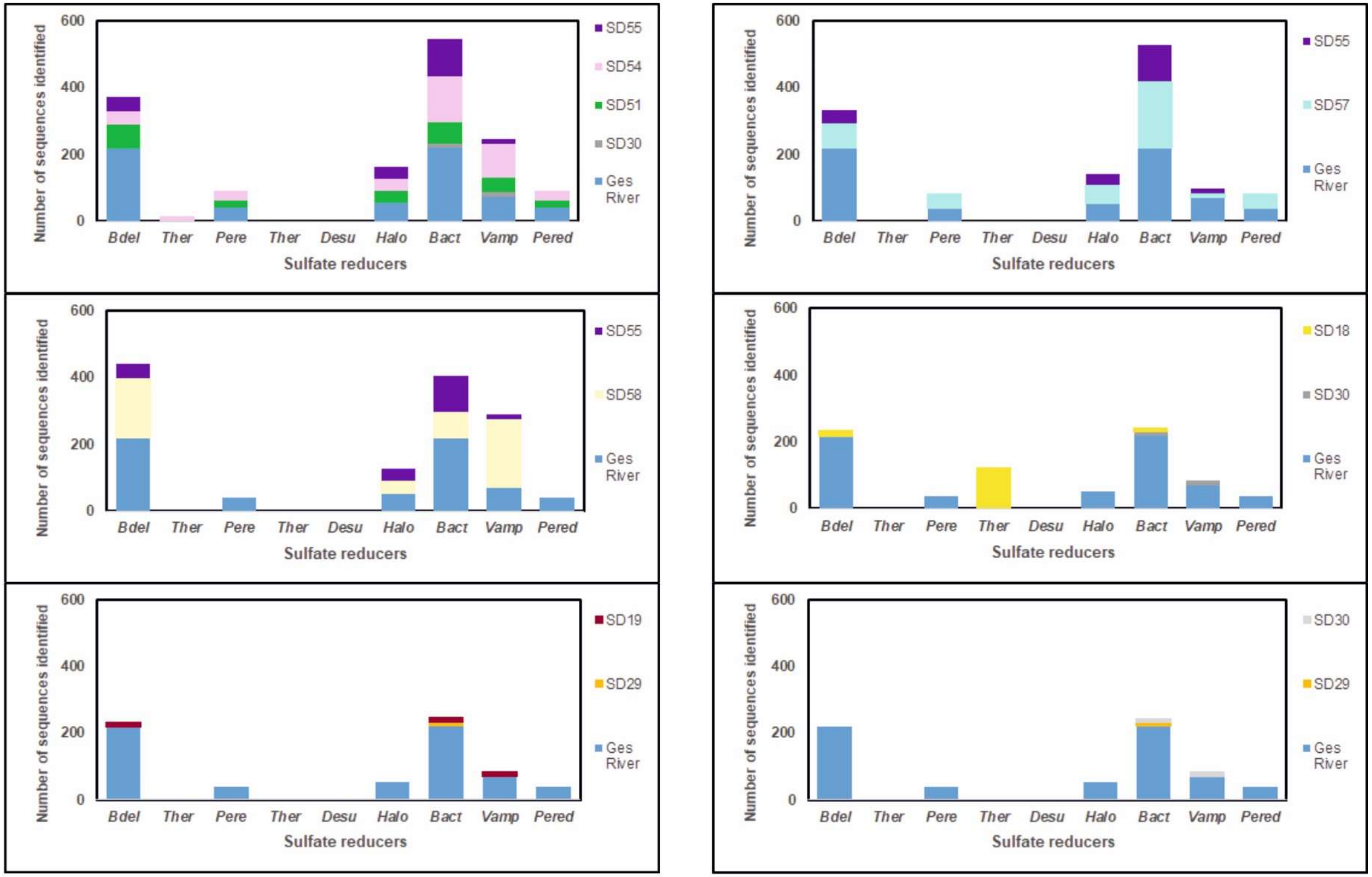

Bdel	***Ther***	***Pere***	***Ther***	***Desu***	***Halo***	***Bact***	***Vamp***	***Pered***
Bdellovibrio	***Thermodesulfovibrio***	***Peredibacter***	***Thermofilum***	***Desulfobacula***	***Halobacteriovorax***	***Bacteriovorax***	***Vampirovibrio***	***Peredibacter***

d)

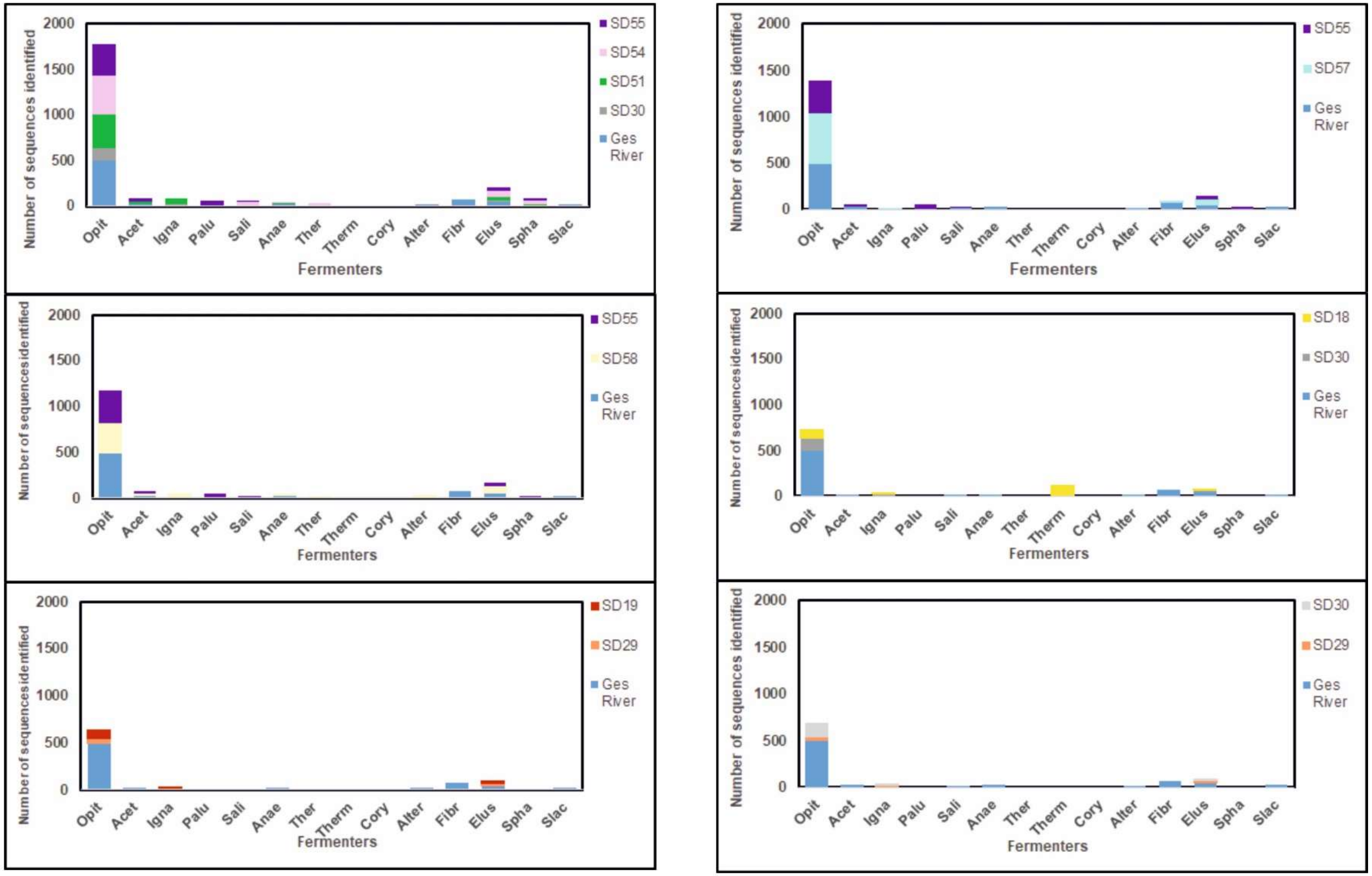

Opit	*Acet*	*Igna*	*Palu*	*Sali*	*Anae*	*Ther*
Opitutus	*Acetobacteroides*	*Ignavibacterium*	*Paludibacter*	*Salinispira*	*Anaerocella*	*Thermogutta*
Therm	*Cory*	*Alter*	*Fibr*	*Elus*	*Spha*	*Slac*
Thermofilum	*Corynebacterium*	*Alterococcus*	*Fibrobacter*	*Elusimicrobium*	*Sphaerochaeta*	*Slackia*

Figure SI–7. . Identified genus capable of sulfide oxidation (a), methanogenesis (b), sulfate reduction (c) and fermenting (d) in the study site. Each color within the bars represents a piezometer within the monitoring network of the Catalan Water Agency.. Spring 2022 sampling survey.
